# Biosynthetic pathway of prescription cucurbitacin IIa and high-level production of key triterpenoid intermediates in engineered yeast and tobacco

**DOI:** 10.1016/j.xplc.2024.100835

**Published:** 2024-02-29

**Authors:** Geng Chen, Zhaokuan Guo, Yanyu Shu, Yan Zhao, Lei Qiu, Shaofeng Duan, Yuan Lin, Simei He, Xiaobo Li, Xiaolin Feng, Guisheng Xiang, Bo Nian, Yina Wang, Zhiyuan Li, Chongkang Yang, Yang Shi, Yingchun Lu, Guanze Liu, Shengchao Yang, Guanghui Zhang, Bing Hao

**Affiliations:** 1 State Key Laboratory of Conservation and Utilization of Bio-resources in Yunnan, The Key Laboratory of Medicinal Plant Biology of Yunnan Province, National & Local Joint Engineering Research Center on Germplasms Innovation & Utilization of Chinese Medicinal Materials in Southwest China, Yunnan Agricultural University, Kunming 650201, China; 2 Yunnan Characteristic Plant Extraction Laboratory, Kunming, Yunnan 650106, China

**Keywords:** cucurbitacin IIa, oxidosqualene cyclase, OSC, 11-carbonyl-20β-hydroxy-Cuol, *Saccharomyces cerevisiae*, *Nicotiana benthamiana*, *Hemsleya chinensis*

## Abstract

Cucurbitacin IIa is a triterpenoid isolated exclusively from *Hemsleya* plants and a non-steroidal anti-inflammatory drug that functions as the main ingredient of prescription Hemslecin capsules and tablets in China. Synthetic biology provides new strategies for production of such valuable cucurbitacins at a large scale; however, the biosynthetic pathway of cucurbitacin IIa has been unknown, and the heterologous production of cucurbitacins in galactose medium has been expensive and low yielding. In this study, we characterized the functions of genes encoding two squalene epoxidases (*HcSE1–2*), six oxidosqualene cyclases (*HcOSC1–6*), two CYP450s (*HcCYP87D20* and *HcCYP81Q59*), and an acyltransferase (*HcAT1*) in cucurbitacin IIa biosynthesis by heterologous expression in *Saccharomyces cerevisiae* and *Nicotiana benthamiana*. We achieved high-level production of the key cucurbitacin precursor 11-carbonyl-20β-hydroxy-Cuol from glucose in yeast via modular engineering of the mevalonate pathway and optimization of P450 expression levels. The resulting yields of 46.41 mg/l 11-carbonyl-20β-hydroxy-Cuol and 126.47 mg/l total cucurbitacin triterpenoids in shake flasks are the highest yields yet reported from engineered microbes. Subsequently, production of 11-carbonyl-20β-hydroxy-Cuol by transient gene expression in tobacco resulted in yields of 1.28 mg/g dry weight in leaves. This work reveals the key genes involved in biosynthesis of prescription cucurbitacin IIa and demonstrates that engineered yeast cultivated with glucose can produce high yields of key triterpenoid intermediates. We describe a low-cost and highly efficient platform for rapid screening of candidate genes and high-yield production of pharmacological triterpenoids.

## Teaser

This work reveals the biosynthetic pathway of prescription cucurbitacin IIa and demonstrates the reconstruction of the cucurbitacin triterpenoid pathway in engineered yeast and tobacco for high-level production.

## Introduction

Cucurbitacins, a class of highly diverse and oxygenated triterpenoids primarily found in the Cucurbitaceae family, exhibit specific and potent bioactivities encompassing the treatment of cancer, inflammatory disease, and diabetes (Chen et al., 2005; Hussain et al., 2019; Ren and Kinghorn, 2019). Cucurbitacins are typically classified into 12 categories, cucurbitacins A–T, based on skeleton oxidation groups (Chen et al., 2005). The cucurbitacin F (CuF) class, which includes cucurbitacin IIa (CuIIa) and cucurbitacin IIb (CuIIb), is predominantly found in plants of the genus *Hemsleya*. Many of these plants are renowned traditional Chinese medicines with well-established pharmacological properties and therapeutic principles in China (Chen et al., 2005). Recently, CuIIa and CuIIb were found to be novel anti-cancer agents; however, the biosynthetic pathway of CuFs is largely unknown (Zhang et al., 2022).

Currently, cucurbitacin biosynthetic pathways are gradually being revealed in some cucurbitaceous plants (Shang et al., 2014; Zhou et al., 2016b; Takase et al., 2019b; Almeida et al., 2022). In *Cucurbita pepo*, the formation of 2,3-oxidosqualene and 2,3:22,23-diepoxysqualene is mediated by three squalene epoxidases (SEs) (Dong et al., 2018). Cucurbitadienol (Cuol) is the first committed precursor of cucurbitacins synthesized from 2,3-oxidosqualene by a specialized oxidosqualene cyclase (OSC) termed Cuol synthase (CBS) (Takase et al., 2019a). After initial construction of the triterpenoid skeleton by OSC, subsequent modifications, including oxidation and acylation, are facilitated by cytochrome P450 monooxygenases (CYPs) and BAHD-acetyltransferases (ATs) (Sawai and Saito, 2011; Seki et al., 2015; Choi et al., 2022).

In *Crocus sativus*, two CYP genes (CsCYP88L2 and CsCYP81Q58) were shown to be involved in cucurbitacin C (CuC) biosynthesis, catalyzing C19β-hydroxylation and C25 hydroxylation of the Cuol backbone (Shang et al., 2014). In *C. melo* and *C. lanatus*, Cm890 (CmCYP87D20) and Cl890 (ClCYP87D20) were identified as catalysts for C11 carboxylase and C20 hydroxylase, and Cm180 (CmCYP81Q59) and Cl180 (ClCYP81Q59) were found to catalyze C2 hydroxylation (Zhou et al., 2016b). CmACT and ClACT were found to catalyze the acetylation of cucurbitacin B (CuB) and cucurbitacin I from cucurbitacin D (CuD) and cucurbitacin E (CuE) in *C. melo* and *C. lanatus*, respectively (Zhou et al., 2016b). Three new CYP genes from *Momordica charantia* involved in cucurbitacin biosynthesis were recently reported: McCYP81AQ19 is responsible for Cuol C23 hydroxylation, McCYP88L7 catalyzes C19 hydroxylation and is involved in formation of C5–C19 ether-bridged products, and McCYP88L8 functions as a C7 hydroxylase (Takase et al., 2019b). In *Iberis amara*, a member of the Brassicaceae family, two species-specific CYPs (CYP708A16 and CYP708A15) were identified that catalyze the unique C16 for C16β-hydroxyl and C22 hydroxylation of the Cuol backbone (Dong et al., 2021). Despite these advances, the precise pathway of CuF biosynthesis remains to be clarified, particularly with regard to C3α-hydroxyl, C16α-hydroxyl, and C22-carbonyl functionalities.

Cucurbitacins, valued for their diverse biological activities, face challenges in pharmaceutical applications due to low plant content and complex extraction procedures. Although synthetic biology shows potential for production of high-value natural drugs (Hesami et al., 2022; Chu et al., 2023; Li et al., 2023), heterologous biosynthesis of cucurbitacins has been limited to the precursor Cuol (Shang et al., 2014; Almeida et al., 2022). Challenges include unknown enzymes for modification and scarce intermediate compounds for testing of enzyme activity. Heterologous synthesis and metabolic engineering have also emerged as effective strategies, providing ample substrates for validation and enabling high-throughput screening (Liu et al., 2018; Tu et al., 2020). Therefore, establishing a tobacco system and chassis for high-yield production of cucurbitacin intermediates is crucial for advancing metabolic engineering efforts.

A previous study reported the construction of two engineered yeast strains, EY10 and EGY48-CpCPQ (CBS in *C. pepo*), for production of Cuol (related output not reported) (Shang et al., 2014; Zhou et al., 2016b; Almeida et al., 2022). The construction strategy was to reuse the inducible pGAL promoter (GAL1 or GAL10), which has problems such as insufficient promoter strength, interference with galactose metabolism, and increased complexity of the *in vivo* expression system (Da Silva and Srikrishnan, 2012; Tang et al., 2020). Recently, strategies involving the use of constitutive promoters for production of triterpenoids have emerged (Wang et al., 2015, 2019; Chen et al., 2020); such strategies can maintain relatively stable transcript levels that are virtually unaffected by intracellular or extracellular stimuli (Tang et al., 2020). For instance, the yeast strain CS-021 achieved a promising titer of 63.00 mg/l through integration of the *SgCBS* gene and maintenance of a sufficient squalene precursor supply (Qiao et al., 2019). Collectively, these findings suggest that metabolic engineering with constitutive promoters holds promise for enhancing cucurbitacin synthesis. For heterologous expression in a plant system, *Agrobacterium-mediated* transient expression in *Nicotiana benthamiana* is an efficient synthetic biological platform for triterpene production (Reed et al., 2017; Reed and Osbourn, 2018). A previous study reported that transient co-expression of CpSE2 and CpCPQ in *N. benthamiana* resulted in Cuol production of 0.02 ng/g dry weight (dw), and such low yields limit cucurbitacin production (Dong et al., 2018).

**Figure 1:**
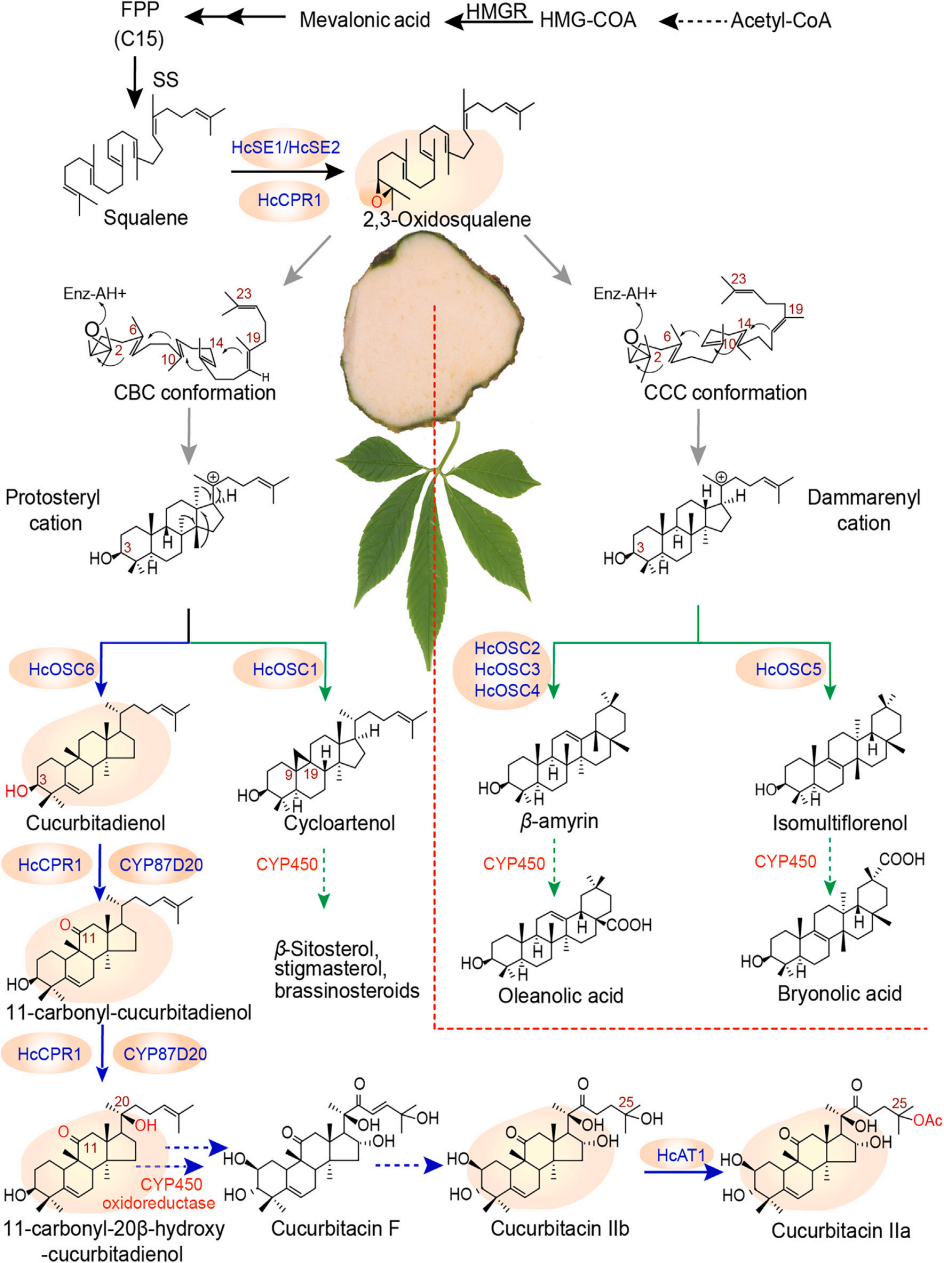
Proposed biosynthetic pathway of cucurbitacin F in *Hemsleya chinensis*. The blue arrows indicate the proposed cucurbitacin F biosynthetic pathway, and the green arrows indicate proposed phytosterol and pentacyclic triterpene biosynthetic pathways. Dotted arrows indicate one or multiple proposed reactions steps; solid arrows indicate identified reactions. Enzymes identified in this study are in blue, and unidentified enzymes are in red. Enzyme abbreviations: SE, squalene epoxidase; OSC, oxidosqualene cyclase; CYP450, cytochrome P450 monooxygenase; AT, BAHD-acetyltransferase. Other abbreviations: CBC, chair-boat-chair; CCC, chair-chair-chair.

In this study, we functionally characterized key *SEs*, *OSCs*, *CYPs*, and *ATs* from *H. chinensis* (Figure 1). A Cuol-producing yeast chassis (Cuol01-1) was constructed by overexpressing and optimizing pathway genes, producing 133.21 mg/l and a maximum titer of 794.7 mg/l Cuol from glucose in shake flasks and fed-batch fermentation, respectively. A series of chassis cells based on Cuol01-1 were constructed by optimizing the oxidation efficiency of C11=O and C20-OH. Efficient CYP87D20 catalytic elements from other species were screened to increase compatibility and elevate CYP87D20 expression levels. By combining all these engineering strategies, we constructed a yeast cell factory (DNCm87-03) that could produce 46.41 mg/l of 11-carbonyl-20β-hydroxy-Cuol and 126.47 mg/l of total cucurbitacin triterpenoids in shake flasks, the highest yields reported to date.

## Results

### CuIIa accumulation in tubers of *H. chinensis*

**Figure 2:**
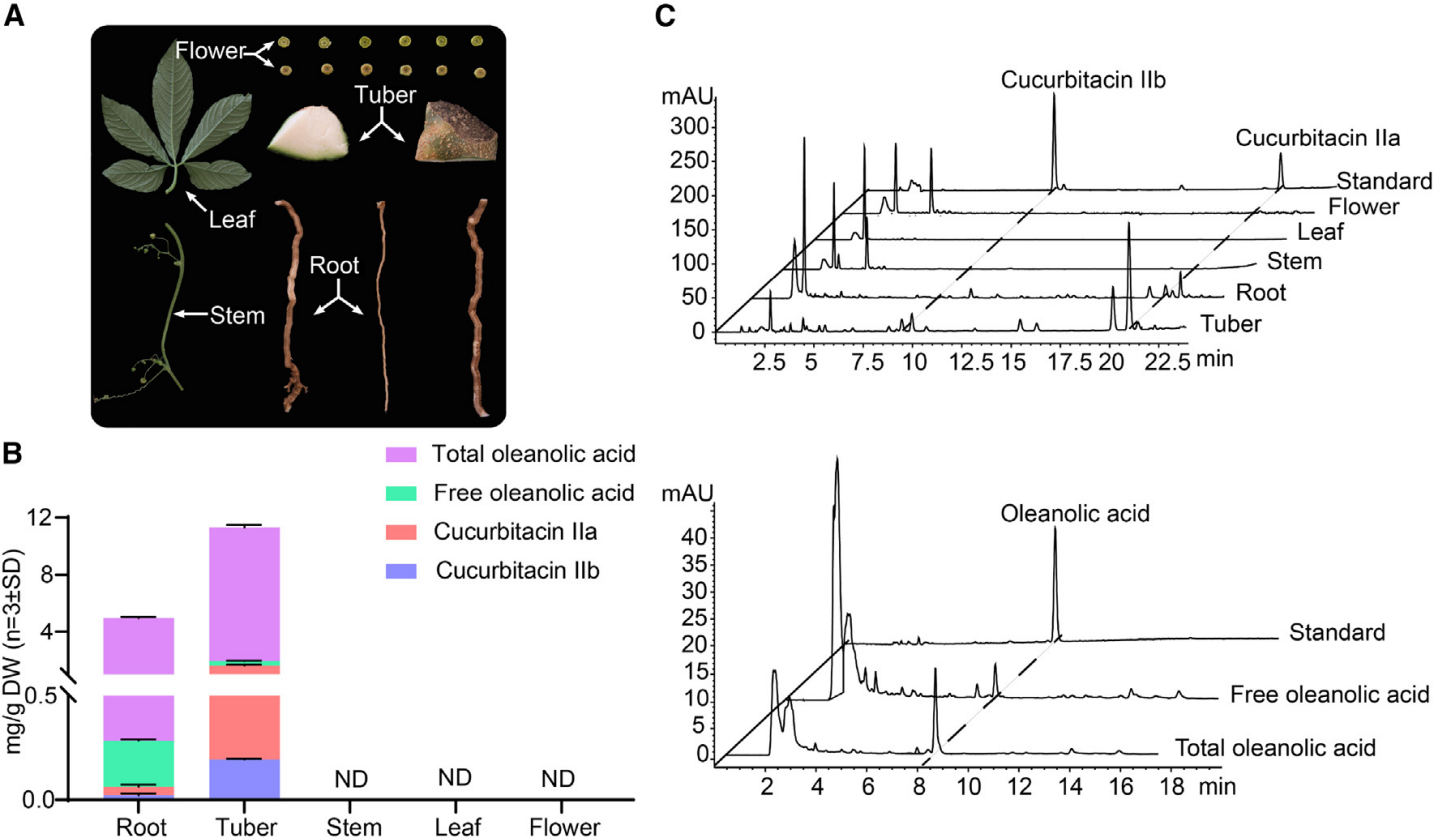
Content of main triterpenoids in *Hemsleya chinensis*. **(A)** Images of different tissues of *H*. *chinensis* and RNA samples for RNA-seq analysis. **(B)** Quantification of cucurbitacin IIa, cucurbitacin IIb, and oleanolic acid in extracts from different *H*. *chinensis* tissues. Means of triplicates and standard deviations are shown (mg/g dw, *n* = 3 ± SE). ND, not detected. **(C)** UHPLC analysis of cucurbitacin IIa, cucurbitacin IIb, and oleanolic acid contents in tissues of *H. chinensis*.

CuIIa and CuIIb are widely distributed in *Hemsleya* plants, particularly in their tubers (Chen et al., 2020). We found that contents of CuIIa and CuIIb were significantly higher in *H. chinensis* than in other species (Supplemental Table 1). Triterpenoid compounds, including CuIIa, CuIIb, and oleanolic acid, accumulated specifically in the tubers, followed by the roots, and were barely detectable in aboveground plant parts (Figure 2B–2D). Biosynthetic pathway genes in plants are often co-regulated and co-expressed in a tissue-specific manner, forming co-expressed functional units that can be characterized by metabolomic and transcriptomic analysis (Lau and Sattely, 2015; Hodgson et al., 2019; Nett et al., 2020). Several studies have shown that co-expression analysis is one of the most useful methods for screening candidate genes. To identify the genetic components involved in cucurbitacin biosynthesis, we obtained RNA sequencing (RNA-seq) data for multiple *H. chinensis* tissues and measured the distribution of CuIIa accumulation for subsequent identification of candidate genes (Figure 2A).

### Functional characterization of *HcSE*, *HcOSC*, and *HcAT* genes

A total of 50 061 733 *H. chinensis* RNA-seq reads were obtained and assembled into 52 923 contigs (Supplemental Table 2) (http://medicinalplants.ynau.edu.cn/transcriptomics/213); 32 742 of the contigs were annotated by BLAST searches of the National Center for Biotechnology Information (NCBI) non-redundant, Swiss-Prot, Kyoto Encyclopedia of Genes and Genomes, and Clusters of Orthologous Groups/Eukaryotic Orthologous Groups protein databases.

**Figure 3:**
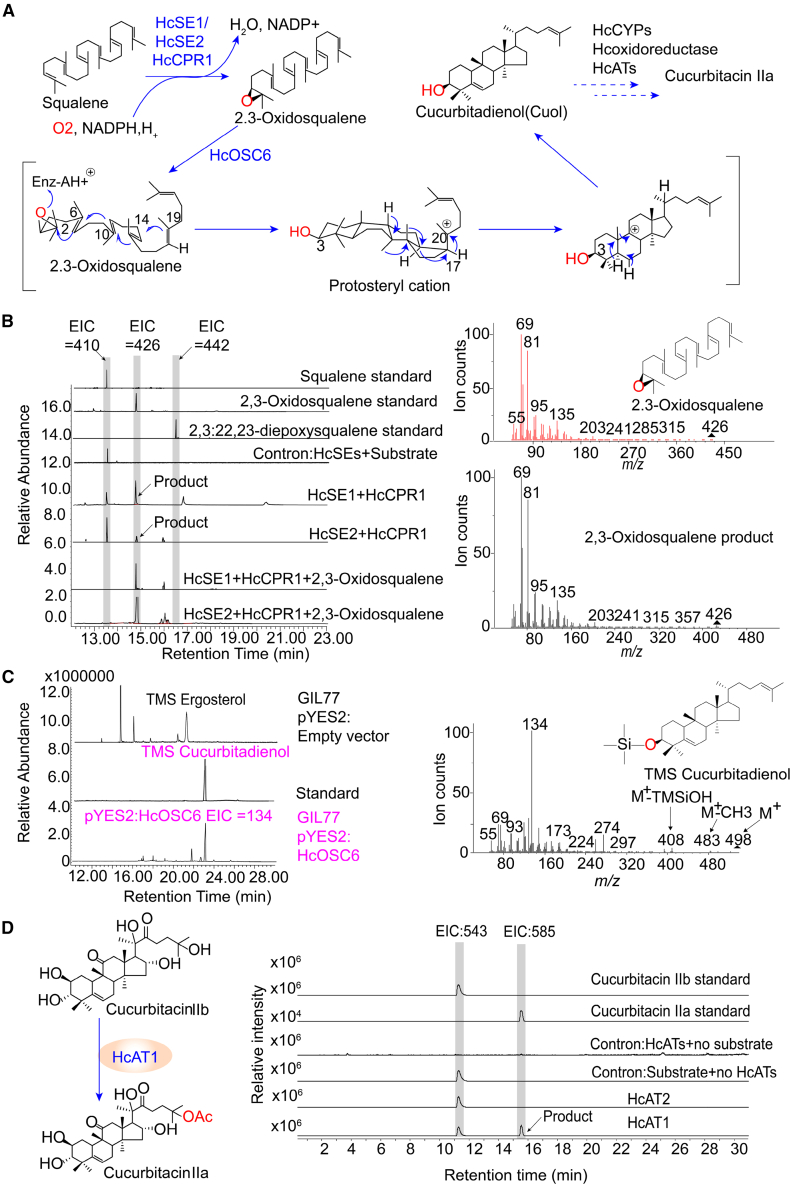
SDS–PAGE of HcSEs, HcCPR1, and HcATs and enzyme activity of recombinant HcSE, HcCPR1, HcOSC6, and HcATs. **(A)** Functional characterization of homologous genes in the biosynthetic pathway of cucurbitacin IIa. **(B)** GC–MS results of the HcSE1, HcSE2, and HcCPR1 enzymatic reactions and MS results revealed the primary ion peak of the 2,3-oxidosqualene standard and the enzymatic product. **(C)** GC–MS analysis of the products in yeast strains containing the HcOSC6 expression plasmids and the empty vector. EIC 134, extracted ion chromatograms of the characteristic fragment ion of cucurbitadienol at a mass/charge ratio (*m/z*) of 134. **(D)** HcAT1 catalyzes the final step of cucurbitacin IIa synthesis and LC–MS results of the HcAT enzymatic reactions. The sample without HcAT1 protein or substrate served as the negative control.

A BLAST search of the transcriptome data using previously reported sequences of SE, CPR, OSC, and AT proteins revealed three *SE* genes, two *CPR* (NADPH-cytochrome P450 reductase) genes, six *OSC* genes, and two *AT* genes (Supplemental Table 3). We used gene-specific primers (Supplemental Table 11) to clone the corresponding sequences from an *H. chinensis* cDNA library and performed additional bioinformatic and phylogenetic analyses of these candidate genes (Supplemental Figures 1–7). A prokaryotic system was used to express their proteins for enzyme activity (Supplemental Figure 8). Three SE candidate genes were ligated into a prokaryotic expression vector for enzyme production, and subsequent detection of the reaction products was performed by gas chromatography–mass spectrometry (GC–MS). The results revealed the emergence of a peak at 16.02 min, which was identified as 2,3-oxidosqualene on the basis of characteristics exhibited by the primary ion peaks. However, no signal for 2,3:22,23-diepoxysqualene was detected in any of the experimental groups, indicating that CuF is likely derived directly from 2,3-oxidosqualene rather than 2,3:22,23-diepoxysqualene in *H. chinensis* (Figure 3B). We failed to express HcSE3 protein in *Escherichia coli* (BL21 DE3), and no further enzyme activity tests were performed. These results indicate that the functions of the HcSEs differ from those of the CpSEs in *C. pepo*, and functional validation of SEs in other cucurbits have not been reported (Dong et al., 2018). 2,3-oxidosqualene was not detected in the absence of HcCPR1 when squalene was supplemented as a control (Figure 3B), as the epoxidation of squalene necessitates the involvement of an NADPH-dependent cytochrome P450 reductase. A previous study demonstrated that formation of 2,3-oxidosqualene occurred exclusively when squalene and NADPH were incubated with the recombinant proteins DzSE and DzCPR derived from *Dioscorea zingiberensis* (Song et al., 2019a).

Multiple sequence alignments revealed that the deduced amino acid sequences of the six *HcOSCs* showed 42.71%–68.93% similarity (Supplemental Figure 2). All six *HcOSCs* contained the DCTAE motif, which is involved in substrate binding (Ito et al., 2013), and four QW motifs characteristic of the OSC superfamily (Supplemental Figure 3). The QW motifs may be involved in stabilizing carbocations during cyclization (Srisawat et al., 2019). *HcOSC2*, *HcOSC3*, and *HcOSC4* had the MWCYCR motif, which is predicted to be a highly conserved motif of β-amyrin synthase (Zhou et al., 2019). To identify the cyclase that catalyzes the first step in formation of the cucurbitacin skeleton, we subcloned the complete open reading frames (ORFs) of six *HcOSCs* into the pYES2 vector and transformed the resulting constructs into lanosterol synthase-deficient yeast (GIL77). GC analysis revealed that extracts from GIL77 yeast expressing HcOSC1 exhibited a distinct peak at 22.10 min whose retention time and mass spectral characteristics were identical to those of the purified cycloartenol standard (Supplemental Figure 9A). Extracts from yeast harboring HcOSC2, HcOSC3, and HcOSC4 contained β-amyrin. The retention time of their product was consistently 24.80 min, and its mass spectral characteristics matched those of the authentic β-amyrin standard (Supplemental Figure 9B). The GIL77 strain expressing HcOSC5 produced a distinct product with a retention time of 24.20 min whose mass spectral characteristics were identical to those of the purified isomultiflorenol standard (Supplemental Figure 9C). Thin layer chromatography (TLC) showed that GIL77 strains expressing *HcOSC6* and *McCBS* (as a positive control) produced a product with the same R_f_ value as the purified Cuol standard (Supplemental Figure 10A). This product exhibited a retention time and mass spectral characteristics identical to those of the purified Cuol standard, with an elution peak at 22.60 min in the GC–MS analysis (Figure 3C). The products of HcOSC1, HcOSC5, and HcOSC6 were also subjected to nuclear magnetic resonance (NMR) spectroscopy, and their NMR data were consistent with previous reports (Supplemental Figures 11–13 and Supplemental Table 4) (DavidáNes, 1991; Isaev, 1995; Yoshida et al., 1989). The results obtained from analysis of GC–MS and NMR spectra thus provide compelling evidence that HcOSC6 functions as a Cuol synthase.

CuIIb was used as the substrate for *in vitro* enzymatic reactions, and high-performance liquid chromatography (HPLC) revealed that HcAT1 effectively facilitated the acetylation of CuIIb to generate CuIIa (Supplemental Figure 14A). We also examined the enzymatic reaction of HcAT1 using cucurbitacin I as the substrate. The HPLC results demonstrated that HcAT1 also catalyzed acetylation of the C25 hydroxyl group on cucurbitacin I, leading to formation of cucurbitacin E (Supplemental Figure 14B). This finding was confirmed by liquid chromatography–mass spectrometry (LC–MS) using a commercial CuIIa standard. The product peak matched that of the CuIIa standard at 20.02 min, and their characteristic peaks were consistent; CuIIa was not detected in the reaction catalyzed by HcAT2 (Figure 3D). The parent mass of cucurbitacin IIa was determined to be 562.35, and the fragment feature exhibited a mass value of 585.35, indicating sodium adduct formation [M + Na]^+^ (Supplemental Figure 14C). These results confirmed that HcAT1 catalyzes the final step in the CuIIa pathway.

### Identification of candidate genes involved in skeleton oxidation modification

**Figure 4:**
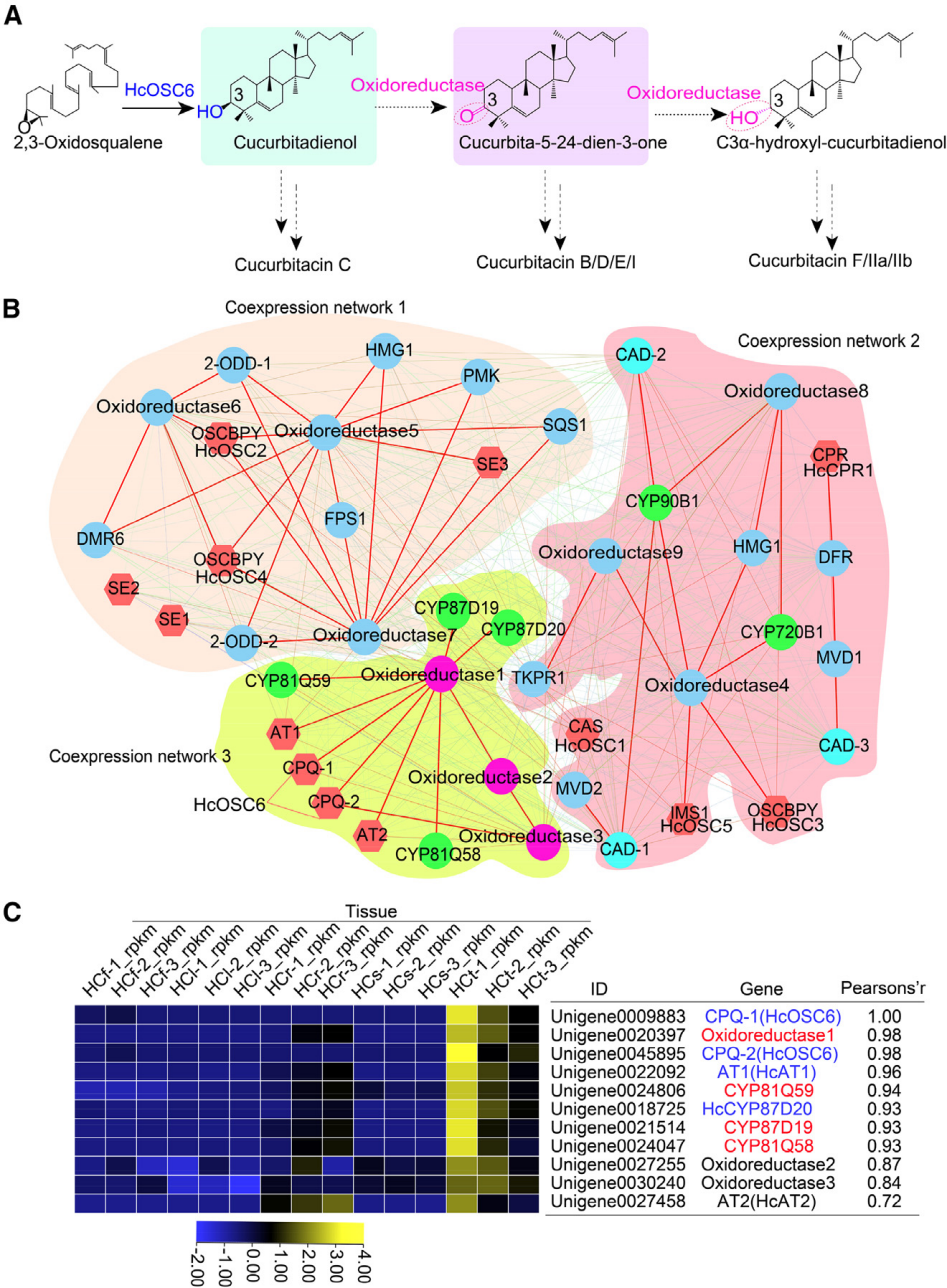
Identification of candidate genes involved in CuIIa biosynthesis by WGCNA and co-expression analysis and analysis of tissue expression of *HcSE*, *HcOSC*, and *HcAT* genes. **(A)** Cuol undergoes epimerization mediated by an oxidoreductase enzyme to yield cucurbita-5-24-dien-3-one and C3α-hydroxyl cucurbitadienol. **(B)** Identification of 36 candidate genes involved in triterpene and steroid biosynthesis by WGCNA. **(C)** Identification of 11 candidate genes involved in CuIIa biosynthesis by co-expression analysis and Pearson’s correlation coefficients of the *Oxidoreductase1* gene and *CYP87D19* with bait genes in the pathway.

To identify genes encoding oxidative-modification enzymes in CuIIa biosynthesis, we performed weighted gene co-expression network analysis (WGCNA) and gene co-expression correlation analysis. The transcriptome data from *H. chinensis* were divided into 17 modules based on their expression patterns, and *HcOSC6* and *HcAT1* with confirmed functions were used as bait genes to identify candidate genes. Thirty-six candidate genes were enriched in the terpenoid skeleton biosynthetic pathway (ko00900), sesquiterpene and triterpene biosynthesis pathway (ko00909), and steroid biosynthesis pathway (ko00100) (Supplemental Table 5) and were mainly assigned to the gray60, coral1, brow4, and other modules (Supplemental Figure 15). According to their correlation and connectivity values, the 36 candidate genes were displayed as three gene co-expression networks using Cytoscape software; genes involved in the oleanolic acid, saponin, and steroid pathways were found mainly in co-expression networks 1 and 2 (Figure 4B). One of the *CYP90B1*s was predicted to catalyze the hydroxylation of C22 and C16 on cholesterol (Christ et al., 2019). Eleven genes formed a strongly correlated co-expression network, network 3 (Figure 4B), which included the four *CYPs* *HcCYP87D20*, *HcCYP81Q58*, *HcCYP81Q59*, and *HcCYP87D19*. *HcCYP87D20* was predicted to be a homolog of *CmCYP87D20* and *ClCYP87D20*, which act as a C11 carboxylase and a C20 hydroxylase of Cuol; *HcCYP81Q58* was predicted to be the homolog of *CsCYP81Q58*, which acts as a C25 hydroxylase of Cuol; and *HcCYP81Q59* was predicted to act as a C2 hydroxylase, producing 11-carbonyl-20β-hydroxy-Cuol (Supplemental Table 5). A *CYP87D19* gene was also identified; its homolog *CsCYP87D19* was previously identified as a member of a gene cluster involved in CuC biosynthesis, but its function has not yet been determined (Shang et al., 2014). Phylogenetic analysis of the *CYP* sequences revealed that *HcCYP87D19* was located in a clade that contained *MlCYP87D16*, whose encoded enzyme catalyzes the C-16_α_ oxidation of β-amyrin in *Maesa lanceolata* (Supplemental Figure 7) (Moses et al., 2015)*. HcCYP87D19* was therefore predicted to be a hydroxylase for C16_β_-hydroxylation of Cuol.

Three oxidoreductase family genes were also identified, with *oxidoreductase1* emerging as the central gene (Supplemental Table 6). This gene was predicted to encode zerumbone synthase (ZSD1), an alcohol dehydrogenase responsible for converting 8-hydroxy-a-humulene into zerumbone by reducing hydroxyl groups to carbonyl groups (Okamoto et al., 2011). In *Arabidopsis thaliana*, *At3g29250* (*THAR1*) and *At1g66800* (*THAR2*) encode a pair of promiscuous oxidoreductases; THAR1 was reported to convert the C3_β_-hydroxys of thalianol and related compounds into C3 ketones, whereas THAR2 reduced C3-ketones to 3α alcohols (Huang et al., 2019). Notably, CuF possesses the distinctive molecular feature of a C3α-hydroxyl, which sets it apart from the C3β-hydroxyl of CuC and the C3-ketone of CuB. On the basis of this observation, we hypothesize that HcOSC6 catalyzes the formation of Cuol from 2,3-oxidosqualene and that Cuol subsequently undergoes epimerization mediated by an oxidoreductase enzyme to yield cucurbita-5-24-dien-3-one and C3α-hydroxyl-cucurbitadienol (Figure 4A) (Huang et al., 2019).

To further investigate the co-expression patterns and transcript expression profiles of these 11 genes, we performed hierarchical clustering and Pearson’s correlation analysis (Figure 4C). We observed high expression levels of candidate genes involved in cucurbitacin biosynthesis in tubers (Supplemental Figure 16), consistent with the distribution patterns of CuIIa and CuIIb in *H. chinensis* (Figure 2). Pearson’s correlation coefficients between the oxidoreductase1 gene and CYP87D19 and bait genes in the pathway were greater than 0.93 (Supplemental Table 6). Overall, our findings suggest that *oxidoreductase1* and *CYP87D19* are highly promising novel candidate genes implicated in oxidative modification of the cucurbitacin skeleton.

### Construction of a yeast strain with high Cuol production

**Figure 5:**
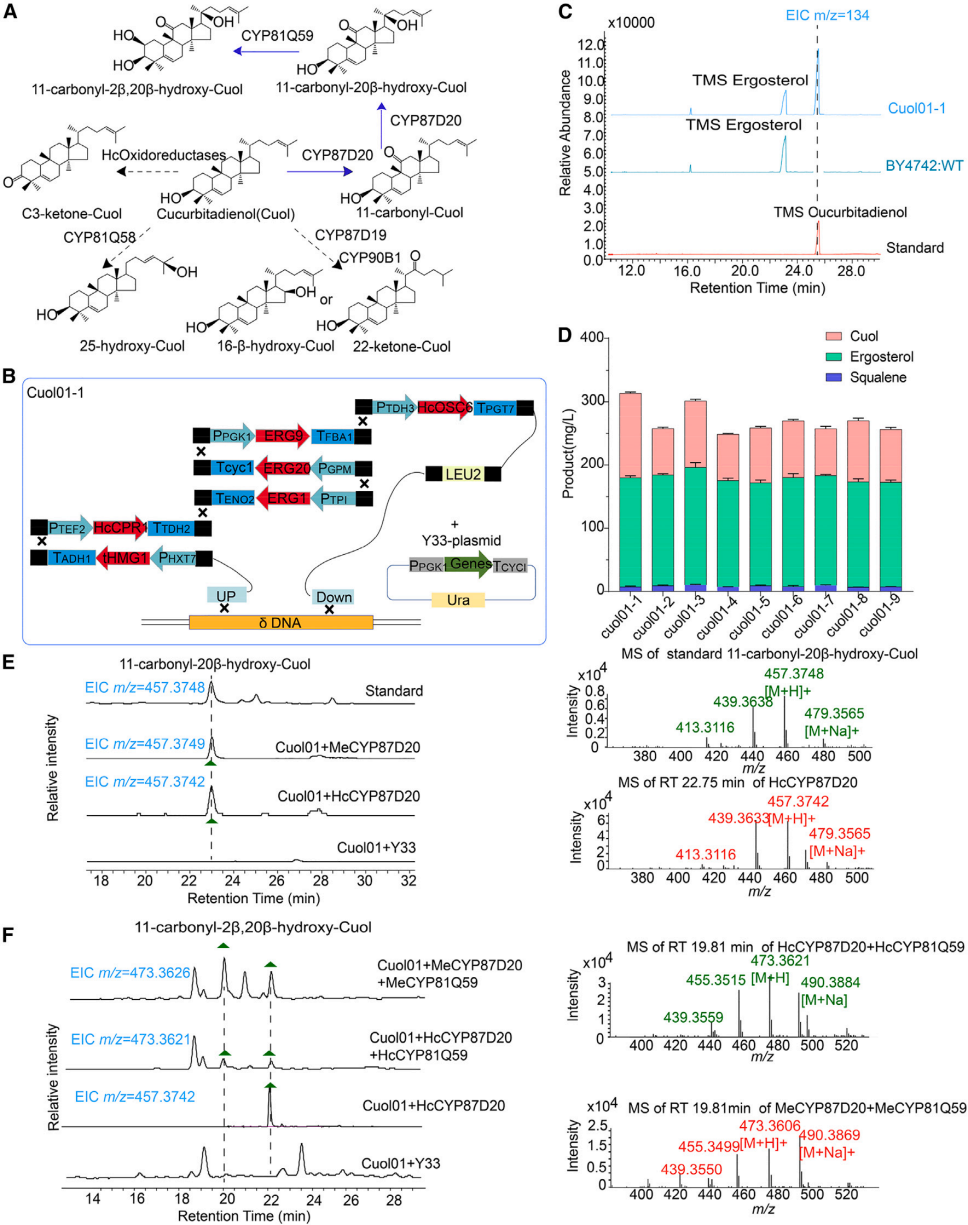
Construction of an engineered yeast platform with high-level production of Cuol to characterize the functions of candidate oxidation-modification enzyme genes. **(A)** Identified oxidative-modification enzyme genes related to CuIIa biosynthesis. **(B)** Design of the biosynthetic pathway in the engineered yeast platform (Cuo101-1); oxidative-modification enzyme genes were later verified by plasmid sequencing. **(C)** GC–MS detection of Cuol accumulation in the engineered yeast strain Cuol01-1. **(D)** Cuol production by different strains in shake flasks. **(E)** LC–MS reveals that HcCYP87D20 and MeCYP87D20 are functional in yeast. Overlaid ion chromatograms of extracts from strains expressing the target genes or the empty vector control (Cuol01-Y33). Peaks potentially corresponding to saponins are labeled with the *m/z* value of 11-carbonyl-20β-hydroxy-Cuol (*m/z* 456). **(F)** LC–MS reveals that HcCYP81Q59 and MeCYP81Q59 are functional in yeast. Overlaid ion chromatograms of extracts from strains expressing the target genes or the empty vector control (Cuol01-EV). Peaks potentially corresponding to saponins are labeled with the *m/z* value of 11-carbonyl-2β,20β-hydroxy-Cuol (*m/z* 472). New peaks in the engineered strains are marked by green triangles, and the MS/MS spectra are shown.

We next aimed to construct an engineered yeast platform for high-level Cuol production in order to characterize the functions of candidate genes encoding oxidation-modification enzymes (Figure 5A and 5B). Because 2,3-oxidosqualene is a key precursor of Cuol, we used previously reported methods to overexpress truncated 3-hydroxy-3-methyl glutaryl coenzyme A reductase (*tHMG1*), farnesyl diphosphate synthase (*ERG20*), squalene synthase (*ERG9*), and 2,3-oxidosqualene synthase (*ERG1*) genes in the engineered yeast strain EY10 (Shang et al., 2014; Wang et al., 2019). HcOSC6 and HcCPR1 were also characterized and overexpressed to ensure sufficient Cuol production (Supplemental Figure 17A and 17B). We used a different constitutive promoter strategy to integrate seven expression-cassette DNA fragments into the delta DNA site of the yeast BY4742 genome via homologous recombination (Wang et al., 2019) (Supplemental Figure 17A). Cuol in the engineered yeast was analyzed and identified using TLC and GC–MS (Figure 5C and Supplemental Figure 10B) and quantified using purified Cuol as an external standard. This analysis revealed a yield of 133.21 mg/l Cuol from glucose in shake flasks (Supplemental Table 7). We thus successfully developed an engineered yeast platform (Cuol01-1) for production of Cuol, the direct precursor of cucurbitacins (Figure 5E).

To further investigate the yield of our engineered yeast Cuol01-1, we performed a batch fermentation culture of Cuol01-1 with YPAD medium. When the initial medium was depleted of glucose, we started feeding to dynamically adjust the fermentation to maintain the pH at 5.8. Glucose was continuously fed throughout the fermentation process, ensuring that its content did not exceed 5 g/l (Supplemental Figure 17C) to avoid accumulation of ethanol and high osmotic pressure that would affect the growth of cells. After a 12-h lag period, the dynamic fermentation entered the logarithmic growth phase until the dry cell weight of the engineered cells reached 81.6 g/l at 120 h. As the chassis cells entered the logarithmic growth phase, ethanol began to accumulate. The DO (dissolved oxygen) value was maintained at 40% by adjusting the speed and aeration to continuously ferment and accumulate products. The resulting yield of Cuol reached 794.7 mg/l at 120 h (Supplemental Figure 17C), the highest yield yet reported. The Cuol products were prepared by separation and purification (Supplemental Figure 17C). The yield of cucurbitacin could be further optimized by modulation of fermentation conditions in future work.

### Functional characterization of candidate oxidation-modification genes

The ORF sequences of five *CYPs* (*HcCYP87D20*, *HcCYP81Q58*, *HcCYP81Q59*, *HcCYP87D19*, and *HcCYP90B*) and three *oxidoreductases* were cloned and ligated into the expression vector YCplac33-PE (Y33) (Liu et al., 2018). The transformed plasmid expressed each *CYP* and each *oxidoreductase* in Cuol-producing yeast Cuol01-1. An empty Y33 vector was also transformed into Cuol01-1 as a negative control (Supplemental Tables 8–9).

Initially, the expected trimethylsilylated cucurbitacin was not detected in the yeast extract through GC–MS analysis. In EI-MS fragmentation mode, the parent ion (M^+^) of trimethylsilylated Cuol in the control group is 498 (426 + TMSi); CYPs catalyze the hydroxylation modification of Cuol, resulting in a product with an M^+^ of 586 (498 + 16 + TMSi). However, GC–MS analysis of the metabolites resulting from each CYP-catalyzed Cuol did not reveal the presence of a new peak with an M^+^ value of 586. Similarly, in the control group, Cuol without trimethylsilylation treatment exhibited an M^+^ value of 426. Oxidoreductase catalyzes the C3β-hydroxylation of Cuol to form a C3-ketone, resulting in an M^+^ value loss of two H^+^, which corresponds to 424. GC–MS analysis of yeast extract subjected to the oxidoreductase-catalyzed Cuol reaction did not reveal any new peak with an M^+^ value of 424 either. Furthermore, TLC analysis revealed that, compared with the negative control yeast extract, extracts derived from strain Cuol01-1-PCm87 expressing *CmCYP87D20* and strain Cuol01-1-PHc87 expressing *HcCYP87D20* both displayed two distinct bluish-purple spots; however, no additional blue-purple spots were observed in yeast extracts expressing *HcCYP81Q58*, *HcCYP81Q59*, or any oxidoreductase (Supplemental Figure 10C).

We next identified the two products by comparative TLC and LC–MS analysis using 11-carbonyl-Cuol and 11-carbonyl-20β-hydroxy-Cuol standards provided by Prof. Yi Shang (Figure 5E and Supplemental Figure 10C). After fermenting 10 l of yeast strain Cuol01-1-PHc87, we separated and purified the extracted products to obtain the two target compounds, named 87D20-1 and 87D20-2. Subsequent NMR analysis revealed that both compounds exhibited two olefin signals in the low-field region, ^δ^H 5.65 (^δ^C120.8, C-6) and ^δ^H 5.08 (^δ^C124.3–125.1, C-24), and a carbonyl carbon signal, ^δ^C214.7–216.5 (C11-carbonyl). The hydrogen atom on carbon-20 of 87D20-2 was substituted with a hydroxyl group, resulting in a down-field shift of the carbon-20 signal to 75.0 parts per million (ppm) (^δ^C 39.35), indicating that 87D20-1 corresponded to 11-carbonyl-Cuol and 87D20-2 corresponded to 11-carbonyl-20β-hydroxy-Cuol (Supplemental Figures 18 and 19 and Supplemental Table 4). The obtained NMR data were consistent with previous literature reports (Zhou et al., 2016b).

We fermented the Cuol01-1-PHc87 strain in shake flasks and quantitatively analyzed the yeast extract using HPLC. The yields of Cuol, 11-carbonyl-Cuol, and 11-carbonyl-20β-hydroxy-Cuol were 123.56 , 4.27, and 1.52 mg/l, respectively (Supplemental Table 7). However, the conversion efficiency of HcCYP87D20 in converting Cuol to 11-carbonyl-Cuol and 11-carbonyl-20β-hydroxy-Cuol was only 4.5% (calculated as [11-carbonyl-Cuol + 11-carbonyl-20β-hydroxy-Cuol]/[Cuol + 11-carbonyl-Cuol + 11-carbonyl-20β-hydroxy-Cuol]). Compared with other candidate oxidation-modification enzymes that lack the ability to catalyze synthesis of novel products from Cuol, HcCYP87D20 exhibits dominant catalytic activity by using Cuol as a substrate to generate downstream products. This finding is reminiscent of our previous study in which co-expression of *Cs890* (*CsCYP87D20*) and *Cs540* (*CsCYP88L2*) in Cuol-producing yeast resulted in detection of both 19-hydroxycucurbitadienol and 11-carbonyl-20-hydroxycucurbitadienol, highlighting the superior catalytic efficiency of CYP87D20 relative to CYP88L2 (Zhou et al., 2016b). We therefore speculate that HcCYP87D20 may serve as the initial catalyst for oxidative modification subsequent to skeleton formation, whereas 11-carbonyl-20β-hydroxy-Cuol could potentially function as the primary intermediate in CuIIa biosynthesis.

We also explored the oxidative modification step after the formation of 11-carbonyl-20β-hydroxy-Cuol. A double-gene recombinant vector carrying *HcCYP87D20* and *HcCYP81Q59* was constructed by Gibson assembly (Gibson et al., 2009), and a recombinant vector carrying *CmCYP87D20* and *CmCYP81Q59* (C2 hydroxylase) was constructed as a positive control. Transfer of the combined vectors into the Cuol-producing yeast strain Cuol01-1 produced the novel yeast strains Cuol01-1-PHc87+HcQ59 and Cuol01-1-PCm87+CmQ59 (Supplemental Tables 8–9). LC–MS analysis revealed the expected peak (mass/charge [*m*/*z*] 473.3620 [M + H]^+^) in the yeast extract of Cuol01-1-PHc87+HcQ59, and an identical peak was also observed at 19.81 min in the positive control strain Cuol01-1-PCm87+CmQ59. We analyzed the fragment ion mass spectrum of this peak (positive ion mode, collision voltage 20 V) and found that it was consistent with the reported fragment ion mass spectrum of 11-carbonyl-2β,20β-dihydroxy-Cuol (Figure 5F). Specifically, the fragment peaks at *m/z* 455 and 437 are the quasi-molecular ion peaks of the compound, with 1 H_2_O and 2 H_2_O removed in sequence, and the important fragment ion at *m/z* 179 produced by cleavage of its side chain with the loss of molecular weight of 126 (C_8_H_14_O) revealed additional oxygen on the side chain (Supplemental Figure 20) (Zhou et al., 2016a). Unfortunately, despite performing shake flask fermentation and cultivating 20 l of yeast, we were unsuccessful in isolating the target compound owing to its exceptionally low yield. Only milligram-level 11-carbonyl-2β,20β-dihydroxy-Cuol was obtained by culturing yeast harboring *CmBi* (*CBS* in *C. sativus*), *CPR*, *Cm890* (*CmCYP87D20*), and *Cm180* (*CmCYP81Q59*) in large-scale shake flasks in a previous study (Zhou et al., 2016b). These previous results led us to speculate that 11-carbonyl-20β-hydroxy-Cuol may not serve as a substrate for CYP81Q59, and further investigations of the mechanisms and genes underlying oxidative modification are warranted.

### High-level production of 11-carbonyl-20β-hydroxy-Cuol in engineered yeast

**Figure 6:**
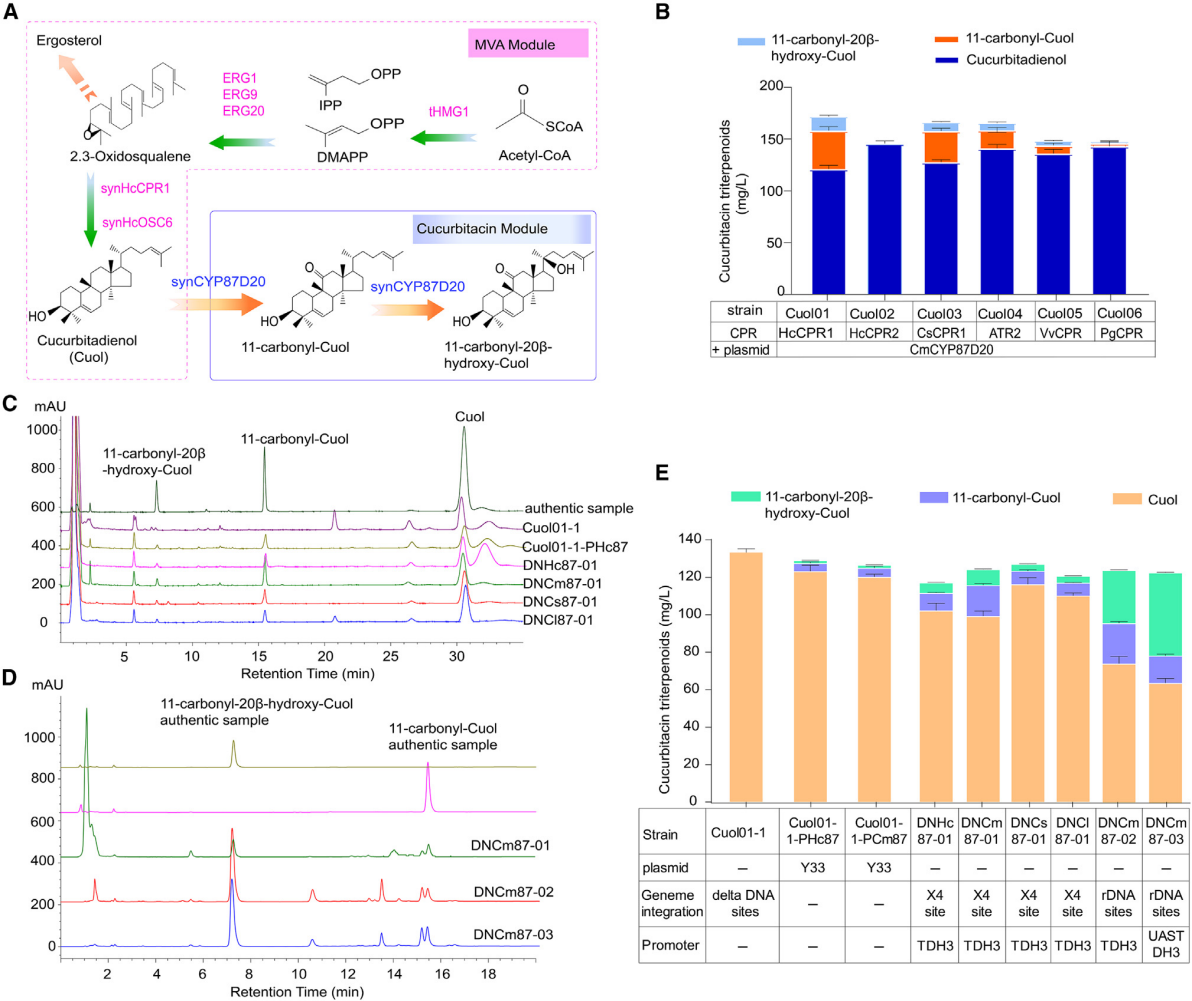
Construction of a platform for production of 11-carbonyl-20β-hydroxy-Cuol in engineered yeast. **(A)** Design of the biosynthetic pathway for production of 11-carbonyl-20β-hydroxy-Cuol in engineered yeast. **(B)** Quantification of triterpenoids in engineered strains harboring various CPRs. **(C)** HPLC analysis of cucurbitadienol (Cuol), 11-carbonyl-Cuol, and 11-carbonyl-20β-hydroxy-Cuol produced by Cuol01-1, Cuol01-1-PHc87, DNHc87-01, DNCm87-01, DNCs87-01, and DNCl87-01. **(D)** HPLC analysis of cucurbitadienol (Cuol), 11-carbonyl-Cuol, and 11-carbonyl-20β-hydroxy-Cuol produced by DNCm87-01, DNCm87-02, and DNCm87-03. **(E)** Quantification of Cuol, 11-carbonyl-Cuol, and 11-carbonyl-20β-hydroxy-Cuol produced by different strains. The error bars indicate the SEM of three biological replicates.

Currently, one of the more effective strategies for enhancing the expression level of *CYPs* involves optimizing the ratio of CYP oxidase and reductase, improving electron transport chain efficiency, and enhancing electron transfer efficiency (Zhu et al., 2018). To optimize the expression of *CYP87D20* in yeast for increased cucurbitacin production (Figure 6A), we synthesized six codon-optimized CPR genes, including two from *H. chinensis* (*HcCPR1* and *HcCPR2*), one from *A. thaliana* (*ATR2*), one from *C. sativus* (*CsCPR1*), one from *Vitis vinifera* (*VvCPR1*), and one from *Panax ginseng* (*PgCPR1*) (Supplemental Figure 18A). We selected HcCPR1 (87.72%) and HcCPR2 (45.55%) on the basis of comparison with previously reported CsCPR homologs. To assess coupling efficiency between different *CPRs* and *CYPs*, we used a similar construction method in the engineered yeast strain Cuo01; specifically, candidate *CPRs* were used to replace the *HcCPR1* gene element in Cuo01, resulting in different engineered strains named Cuol02 (*HcCPR2*), Cuol03 (*CsCPR1*), Cuol04 (*ATR1*), Cuol05 (*VvCPR1*), and Cuol06 (*PgCPR1*). All engineered strains produced Cuol with yields that did not differ significantly from that of Cuol01 (*HcCPR1*).

Under the control of the PTDH3 promoter, *CmCYP87D20* was inserted into the single-copy X4 site of the engineered strains constructed above. The coupling efficiency was evaluated by monitoring the process of 11-carbonyl-Cuol and 11-carbonyl-20β-hydroxy-Cuol production. HPLC analysis showed that the engineered strain containing Cuol01 (*HcCPR1*) produced the highest titer of total triterpenes. No fermentation products were detected in the engineered yeast containing *HcCPR2*, but the other five CPRs were able to transfer electrons to the CYP. The relative amounts of fermentation products produced by engineered yeast with different CPRs were ranked *HcCPR1* > *CsCPR1* > *ATR2* > *VvCPR1* > *PgCPR1* > *HcCPR2* (Figure 6B). These results indicated that CPRs had varying degrees of influence on the catalytic activity of CYP87D20, which in turn affected the production of cucurbitacin triterpenes.

To enhance the yield of 11-carbonyl-Cuol and 11-carbonyl-20β-hydroxy-Cuol, we screened alternative CYP87D20 enzymes from four species to identify an efficient biosynthetic pathway for 11-carbonyl-20β-hydroxy-Cuol. Among the screened enzymes, only *Cs890*, *Cm890*, *Cl890A*, and *Cl890B* were found to participate in this catalytic reaction. Their amino acid sequences showed 83.01%–84.21% homology (Supplemental Figure 21C and Supplemental Table 3), which greatly limited the development of the current optimization strategy. To assess the biosynthetic efficiency of different CYP87D20s under the control of the strong structural promoter *P*_*TDH3*_, four CYP87D20 genes were synthesized by codon optimization (*synHcCYP87D20*, *synCmCYP87D20*, *synCsCYP87D20*, and *synClCYP87D20*) and introduced into the single-copy X4 site of Cuol01-1 (Figure 6E) (Dai et al., 2014). The 11-carbonyl-Cuol and 11-carbonyl-20β-hydroxy-Cuol yields of the four resulting strains (DNHc87-01, DNCm87-01, DNCs87-01, and DNCl87-01) were higher than those of the plasmid-expressing strain Cuol01-1-PHc87. The 11-carbonyl-Cuol and 11-carbonyl-20β-hydroxy-Cuol yields were 1.9- and 1.5-fold higher in DNCm87-01 than in the control strain DNHc87-01. We therefore speculated that the homolog CmCYP87D20 from *C. melo* has the highest catalysis activity for 11-carbonyl-20β-hydroxy-Cuol production (Figure 6C–6E and Supplemental Table 7); even so, a large amount of Cuol (99.42 mg/l) still accumulated in DNCm87-01, indicating that only a small amount of Cuol was converted into 11-carbonyl-Cuol and 11-carbonyl-20β-hydroxy-Cuol. The oxidation conversion rate was only 20.2%, and the yield of the intermediate 11-carbonyl-Cuol was doubled compared with 11-carbonyl-20β-hydroxy-Cuol (Figure 6E and Supplemental Table 7).

We attempted to increase *CmCYP87D20* expression by introducing multiple copies of *CmCYP87D20* into the rDNA sites to obtain the strain DNCm87-02, which has hundreds of copies in the yeast chromosome (Nomura et al., 2013) (Supplemental Figure 21C). The 11-carbonyl-Cuol and 11-carbonyl-20β-hydroxy-Cuol yields of DNCm87-02 were increased to 21.7 and 28.45 mg/l, respectively, and the yield of 11-carbonyl-20β-hydroxy-Cuol was greater than that of 11-carbonyl-Cuol (Figure 6E and Supplemental Table 7). The strong artificial promoter (Blazeck et al., 2012) *UAS*_*TEF1*_*-UAS*_*CIT1*_*-UAS*_*CLB2*_*-P*_*TDH3*_ (hereafter referred to as *UAS*-*TDH3*) replaced the *P*_*TDH3*_ promoter in the strain DNCm87-03 (Supplemental Figure 21C). As a result, production of 11-carbonyl-20β-hydroxy-Cuol increased to 46.41 mg/l, and production of 11-carbonyl-Cuol decreased to 15.85 mg/l (Figure 6E and Supplemental Table 7). The production of 11-carbonyl-20β-hydroxy-Cuol was three-fold higher than that of 11-carbonyl-Cuol. Cuol content was reduced to 64.21 mg/l, and the conversion rate of the oxidation product rose to 49.23%. Overall, production of 11-carbonyl-20β-hydroxy-Cuol was increased five-fold by increasing the expression level of *CmCYP87D20* in the Cuol chassis Cuol01-1 (Supplemental Figure 10E). These results suggest that increasing the expression of selected CYP genes can enhance the efficient conversion of Cuol into 11-carbonyl-20β-hydroxy-Cuol. The 11-carbonyl-20β-hydroxy-Cuol-accumulating strain DNCm87-03 can be used as a platform to further explore the functions of candidate enzymes and as a cell factory for production of cucurbitacin intermediates.

### Reconstitution of 11-carbonyl-20β-hydroxy-Cuol biosynthesis in *N. benthamiana* leaves

Plants offer several advantages as heterologous hosts for metabolic engineering, including their safety, cost-effectiveness, and potential for unlimited production of therapeutics in a rapid and flexible manner (Marsian and Lomonossoff, 2016). Compared with yeast systems, plant systems may serve as better hosts for terpenoid production (Zhang et al., 2011; Nett and Sattely, 2021). To enhance the efficiency of leaf infiltration, we used a modified *Agrobacterium* infiltration process based on a previous report (Reed et al., 2017). In brief, *A. tumefaciens* strains containing each gene construct were cultured individually and mixed in equal volumes prior to co-infiltration into *N. benthamiana* leaves; six to eight plant leaves were vacuum infiltrated simultaneously to combine expressed genes. To establish a high-efficiency *Agrobacterium-mediated* transient expression system in tobacco leaves, the expression efficiency of *GFP* in different *Agrobacterium* strains was screened by OD_600_ values and vacuum osmotic pressure. Three *Agrobacterium* strains (GV3101, EHA105, and LBA4404) carrying the *35Spro*:*GFP* gene vector were infiltrated into tobacco, and GFP expression reached the highest level at 4 days after infiltration. Under conditions of OD_600_ = 0.8 and a vacuum pressure of 80 kPa, EHA105 exhibited the highest GFP fluorescence signal in transformed tobacco (Supplemental Figure 22A–22D). EHA105 was therefore considered to be the optimal strain for construction of a transient expression system, consistent with previous findings (Li et al., 2021).

**Figure 7:**
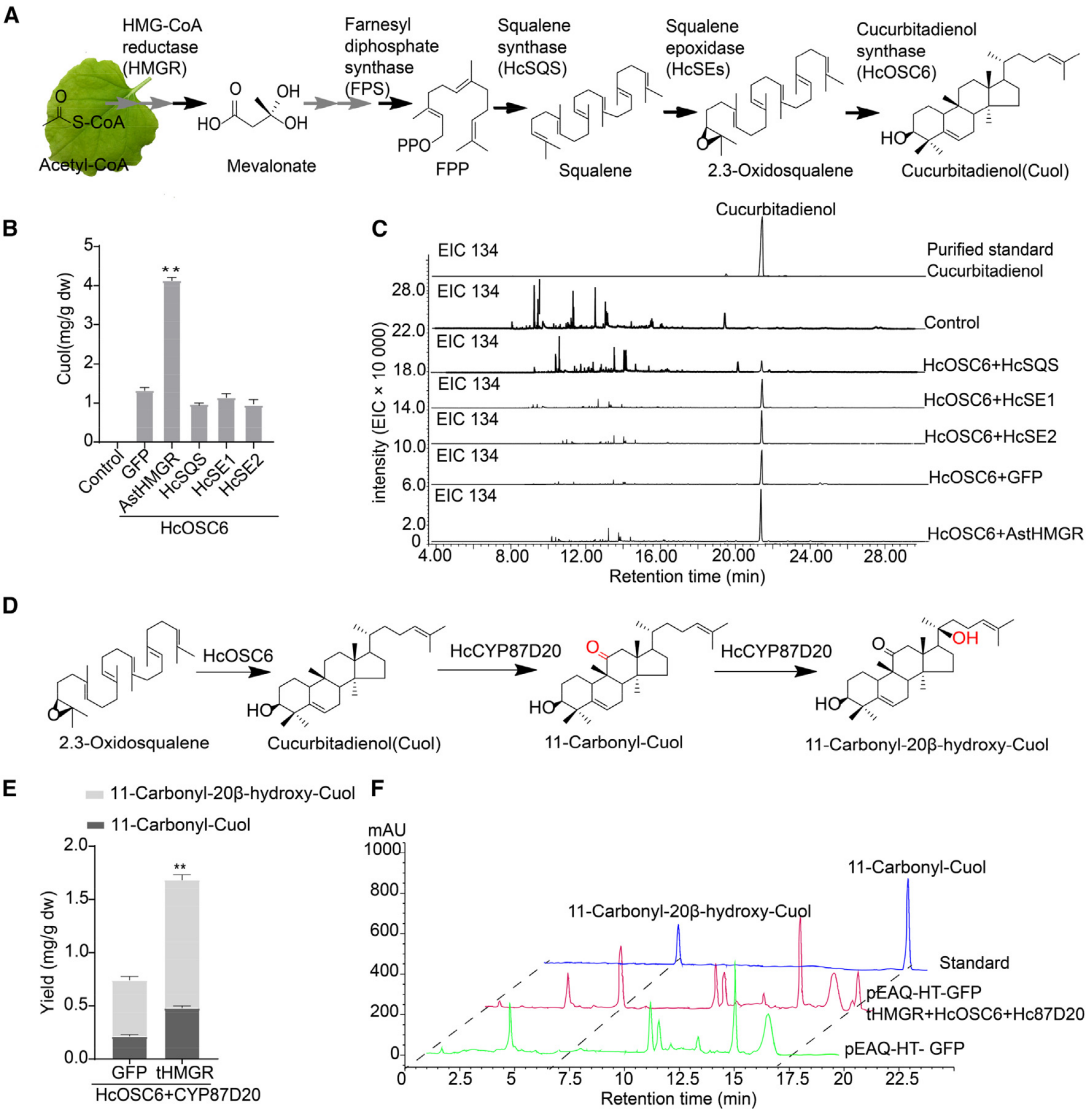
Co-expression with AstHMGR enhances levels of the intermediate 11-carbonyl-20β-hydroxy-Cuol in *N. benthamiana*. **(A)** Biosynthesis of the triterpene cucurbitadienol (Cuol) occurs via the mevalonate pathway. **(B)** Cuol content of tobacco leaves co-expressing *HcOSC6* with *AstHMGR* (mean of three biological replicates ± SE; control, empty vector [EV] or pEAQ-HT-DEST with *GFP* gene). ∗*P* < 0.05. **(C)** Total extracted ion chromatograms (EICs) of extracts from leaves expressing *HcOSC6* with *AstHMGR*. EIC 134, extracted ion chromatograms of the characteristic fragment ion of cucurbitadienol at a mass/charge ratio (*m/z*) of 134. **(D)** Oxygenation of the Cuol scaffold by HcCYP87D20 to produce 11-carbonyl-Cuol and 11-carbonyl-20β-hydroxy-Cuol. **(E)** 11-Carbonyl-Cuol and 11-carbonyl-20β-hydroxy-Cuol contents of leaves expressing *HcOSC6* and *HcCYP87D20* with *GFP* or *tHMGR* (mean of three biological replicates ± SE). ∗*P* < 0.05. **(F)** HPLC analysis of 11-carbonyl-Cuol and 11-carbonyl-20β-hydroxy-Cuol in tobacco leaf extract.

Because precursor availability might be limited, we co-expressed *HcOSC6* with different upstream mevalonate pathway genes to determine their effects on Cuol production. GC–MS analysis of the *N. benthamiana* leaf extract revealed a single peak at 20.02 min that exhibited a mass fragment pattern and retention time consistent with the purified Cuol standard (Figure 7C). The product was quantified using Cuol as the external standard (Supplemental Table 10). The results demonstrated that expression of squalene synthase (*HcSQS*) and *HcSE* genes had little impact on Cuol content, whereas the AstHMG-CoA reductase (*tHMGR*) gene produced a significant, three-fold increase in Cuol yield (Figure 7B). The total Cuol content was 1.32 mg/g (dw) when *HcOSC6* was expressed (*35S*_*pro*_:*HcOSC6* + *35S*_*pro*_:*GFP*) and 4.18 mg/g (dw) when *HcOSC6* and *AstHMGR1* were co-infiltrated (*35S*_*pro*_:*HcOSC6* + *35S*_*pro*_:*AstHMGR*). Co-expression of *AstHMGR* can thus significantly increase triterpene production in a transient plant expression system (Figure 7B).

We also transiently co-expressed *HcCYP87D20* in combination with *HcOSC6* and *GFP* in tobacco (35*Spro*:*HcCYP87D20* + *35Spro*:*HcOSC6* + *35Spro*:*GFP*), and *HcCYP87D20* converted Cuol into the expected products, 0.32 mg/g of 11-carbonyl-Cuol and 0.58 mg/g of 11-carbonyl-20β-hydroxy-Cuol (Figure 7E). LC–MS analysis of co-infiltrated tobacco leaf extract revealed an ion peak with *m/z* 457.3746 [M + H]^+^ that showed the same retention time (6.75 min) as the 11-carbonyl-20β-hydroxyl-Cuol standard. No product was found in extract of tobacco infiltrated with the blank control pEAQ-HT-DEST1-GFP (Figure 7F). Transient expression of *HcCYP87D20*, *HcOSC6*, and *AstHMGR* (*35Spro*:*HcCYP87D20* + *35Spro*:*HcOSC6* + *35Spro: AstHMGR*) in tobacco yielded 0.63 mg/g of 11-carbonyl-Cuol and 1.28 mg/g of 11-carbonyl-20β-hydroxy-Cuol. Yields of both 11-carbonyl-Cuol and 11-carbonyl-20β-hydroxy-Cuol were thus increased two-fold when *AstHMGR was included* in the plant transient expression system (Figure 7E). 11-carbonyl-20β-hydroxy-Cuol may be easier to obtain in tobacco leaves than in the engineered yeast strain DNcm87-03. We also tried to transiently co-express *AstHMGR*, *HcOSC6*, *HcCYP87D20*, and *HcCYP81Q59* in tobacco and obtained a specific peak with fragment ions of *m/z* 473.3625, *m/z* 455.3498, and *m/z* 437.3625, indicating the products of quasi-molecular ion peaks that removed 1 H_2_O and 2 H_2_O, similar to the reported 11-carbonyl-2β,20β-dihydroxy-Cuol fragment mass spectra (Zhou et al., 2016b) (Supplemental Figure 22E).

## Discussion

Cucurbitacins have been used extensively in recent decades owing to their diverse biological activities, with CuIIa and CuIIb being utilized as prescriptions. Identifying the enzymes involved in plant cucurbitacin metabolism would potentially enable the synthesis of cucurbitacins through a synthetic approach.

Here, through analysis of the *H. chinensis* transcriptome, we identified and functionally characterized two *SEs* (*HcSE1* and *HcSE2*), *HcOSC6*, *HcCYP87D20*, *HcCPR1*, and *HcAT1* involved in 11-carbonyl-20β-hydroxy-Cuol and CuIIa biosynthesis. WGCNA and co-expression cluster analysis were performed to identify additional genes potentially involved in CuIIa biosynthesis. *HcOSC6*, *HcCYP87D20*, *HcCYP81Q58*, *HcCYP81Q59*, *HcCYP87D19*, *HcOxidoreductase1*, and *HcAT1* emerged as the most significant candidate genes. It is presumed that *HcCYP87D19* catalyzes formation of the C16β-hydroxyl of Cuol and that HcOxidoreductase1 potentially converts the C3β-hydroxy of Cuol into C3 ketones, leading to formation of cucurbita-5-24-dien-3-one.

Functional characterization of six HcOSCs in a lanosterol-synthase-deficient yeast strain revealed their distinct substrate specificity. HcOSC2/3/4 exhibited monofunctional activity, catalyzing the cyclization of 2,3-oxidosqualene to β-amyrin, which suggested their involvement in formation of the oleanolic acid skeleton. Surprisingly, HcOSC5 was identified as an isomultiflorenol synthase, converting 2,3-oxidosqualene to isomultiflorenol, an intermediate in pentacyclic triterpene biosynthesis that is subsequently converted into bryonolic acid (Hayashi et al., 2001). Previous studies have demonstrated accumulation of bryosic acid in roots and cultured cells of diverse Cucurbitaceae species (Cho et al., 1992). Although bryonolic acid has not previously been reported in the *Hemsleya* genus, this study reports for the first time that HcOSC5 (isomultiflorenol synthase) functions as an OSC controlling the biosynthesis of the bryonolic acid skeleton in *H. chinensis*. This finding will contribute to future research on the biosynthetic pathways of bryonolic acid. Furthermore, our study highlights the essential role of multiple OSCs in generating triterpenoid diversity within the *Hemsleya* genus.

To identify the oxidative-modification enzymes involved in CuIIa biosynthesis, we focused specifically on CYPs and oxidoreductases, which have previously been documented to catalyze redox reactions of the triterpene skeleton (Mitsuguchi et al., 2009; Lv et al., 2017; Cao et al., 2019; Huang et al., 2019). We functionally characterized the CYP and oxidoreductase candidate genes by expressing them in the Cuol-producing engineered yeast Cuol01-1. Expression of *HcCYP81Q58*, *HcCYP87D19*, and *HcOxidoreductase1–3* did not yield any new products, indicating that these enzymes were not suitable for catalyzing formation of Cuol. However, HcCYP87D20 and CmCYP87D20 were capable of continuously catalyzing production of 11-carbonyl-Cuol and 11-carbonyl-20β-hydroxy Cuol from Cuol, consistent with the observed catalytic functions of CsCYP87D20, CmCYP87D20, and ClCYP87D20 (Zhou et al., 2016b). The catalysis of C-2 hydroxylation in 11-carbonyl-20β-hydroxy-Cuol to generate 11-carbonyl-2β,20β-dihydroxy-Cuol resulted in an extremely low yield, suggesting that 11-carbonyl-20β-hydroxy-Cuol may not be a substrate for HcCYP81Q59. Consequently, it is plausible to consider HcCYP87D20 the initial enzyme involved in the oxidative modification of Cuol. Further investigations are necessary to explore different combinations of *CYP* and *Oxidoreductase* expression to identify the functional gene and its significance. Furthermore, a high-quality *H. chinensis* genome is necessary for mining candidate genes by gene cluster analysis (Zhou et al., 2016b).

The limited presence of cucurbitacins in Cucurbitaceae plants has hindered the exploration of their pharmaceutical activities and applications. There have also been few reports on the isolation of cucurbitacin intermediates, which exacerbates the challenge of speculating on the cucurbitacin pathway and screening enzyme gene activity (Glotter et al., 1971; Yuan et al., 2020; Zhou et al., 2016). The present study presents a modular metabolic engineering approach in *Saccharomyces cerevisiae* that ensures sufficient precursor supply for the production of three cucurbitacin intermediate compounds. Under shake-flask conditions without process optimization, the chassis strain Cuol01-1 achieved a Cuol production of 133.21 mg/l. By optimizing fed-batch fermentation conditions, the dry cell weight of engineered cells reached 81.6 g/l, and the yield of Cuol approached gram-scale levels at 794.7 mg/l. In addition, through optimization of *CYP* expression levels, high yields of *de novo*–synthesized 11-carbonyl-20β-hydroxy-Cuol (46.41 mg/l) and 11-carbonyl-Cuol (15.85 mg/l) were also obtained from glucose in shake flasks.

Nonetheless, the precursor concentration of Cuol in engineered yeast DNCm87-03 remained at 64.21 mg/l, perhaps because of the metabolic burden resulting from overexpression of multiple genes or the inhibitory effects of triterpenoids on cell growth (Zhu et al., 2018). The strategy used here for construction of engineered yeast involved the use of various constitutive promoters, irrespective of their inducibility or repressibility, ensuring stable expression levels (Da Silva and Srikrishnan, 2012; Tang et al., 2020). This approach has been successfully implemented for production of numerous high-value compounds, including artemisinic acid, ginsenosides, breviscapine, and medicinal tropane alkaloids in yeast (Li et al., 2021; Liu et al., 2018; Ro et al., 2006; Srinivasan and Smolke, 2020; Wang et al., 2021; Wang et al., 2019). High expression of recombinant *CYPs* in the host microorganism is essential for efficient, high-yield biosynthesis of natural products. To achieve high yield through enhanced expression and activity of *CYPs* in the future, we will explore additional metabolic strategies, including N-terminal modification, co-expression of chaperones, supplementation with prosthetic groups, and engineering of the endoplasmic reticulum (Hu et al., 2023). Recently, two multidrug and T-oxygen complex extrusion (MATE) proteins involved in the transport of cucurbitacin have been reported in plants. These proteins facilitate the directional extracellular transport of cucurbitacin, providing an alternative approach to enhancing yield by alleviating the effect of cucurbitacin on cell growth or toxicity, as well as simplifying the process of cucurbitacin purification from the medium (Zhong et al., 2022).

In the tobacco transient expression system, we observed that co-expression with AstHMGR was sufficient to significantly enhance the synthesis of cucurbitacin intermediates. Co-expression of *AstHMGR1* and *HcOSC6* resulted in production of 4.18 mg/g (dw) of Cuol, which was 3.16-fold higher than that achieved by expressing *HcOSC6*, consistent with previous findings (Reed et al., 2017). Similarly, co-expression of *HcOSC6* and *HcCYP87D20* with *AstHMGR* resulted in a two-fold increase in production of 11-carbonyl-20β-hydroxy-Cuol compared with that observed in the absence of *AstHMGR*. Whereas the engineered yeast DNCm87-03 accumulated a substantial amount of the cucurbitacin intermediate 11-carbonyl-Cuol, conversion of the substrate Cuol to 11-carbonyl-20β-hydroxy-Cuol was enhanced in *N. benthamiana*, as plant-derived genes exhibit higher expression efficiency in tobacco than in microorganisms. This is attributed to the ability of tobacco plants to support accurate mRNA and protein processing, precise protein localization, and metabolic compartmentalization, as well as the fact that they possess a rich pool of essential metabolic precursors and co-enzymes (Reed et al., 2017).

Cucurbitacins are triterpenes that undergo extensive oxidative modifications, indicating their derivation from a complex and intricate biosynthetic pathway. Consequently, it is imperative to perform exhaustive screening of numerous genes encoding oxidative-modification enzymes in order to unravel the precise steps involved in the complete cucurbitacin pathway. Combinatorial expression of biosynthetic enzyme-encoding genes in *N. benthamiana* provides a robust platform for characterizing gene functions. For instance, this approach facilitated characterization of the biosynthetic pathway responsible for the distinctive 5,6-spiroketal moiety in diosgenin through simultaneous expression of 29 full-length *Paris polyphylla* CYPs and 33 full-length *Trigonella foenumgraecum* CYPs (Christ et al., 2019). We reasoned that this approach would significantly enhance progress toward elucidating the complete cucurbitacin pathway and enable subsequent identification of specific *CYPs* and *oxidoreductases*. Co-expression of identified genes encoding pathway enzymes together with unknown *CYPs* and *oxidoreductases* in *N. benthamiana* can generate a plethora of cucurbitacin intermediates and monomers, thereby facilitating pathway analysis and heterologous synthesis of cucurbitacin.

## Materials and methods

### Plant materials and transcriptome sequencing

Mature (6-year-old) *H. chinensis* plant tissues were collected from Kunming City in Yunnan Province, China. Roots, tubers, stems, leaves, and flowers were harvested from three individuals, frozen with liquid nitrogen, and sent to Gene Denovo Biotechnology (Guangzhou, China) for library construction. Other *Hemsleya* plants were also used for analysis of CuIIa and CuIIb content (Supplemental Table 1). Plant materials were identified by researcher Yunheng Ji (Kunming Institute of Botany), and the plant specimens are preserved in the herbarium of Kunming Institute of Botany, Chinese Academy of Sciences. Total RNA was extracted from 15 tissues of *H. chinensis* using the HiPure HP Plant Total RNA Kit (Magen, China). The isolated RNA was used for cDNA synthesis according to the manufacturer’s instructions. The PrimeScript II 1st Strand cDNA Synthesis Kit (TaKaRa, Japan) was used for qPCR experiments, and the HiScriptII 1st Strand cDNA Synthesis Kit (Vazyme Biotech) was used for gene cloning experiments. Sequencing was performed on an Illumina HiSeq 4000 platform by Gene Denovo Biotechnology. After purification of cDNA fragments, end repair and poly(A) tailing were performed before ligation to Illumina sequencing adapters. Pre-sequencing assessment of RNA quality was performed using an Agilent 2100 Bioanalyzer (Agilent Technologies, Palo Alto, CA).

### UPLC analysis of CuIIa, CuIIb, and oleanolic acid content in plant tissues

Samples of five tissues (tubers, roots, leaves, stems, and flowers) collected from *H. chinensis* and other species were vacuum freeze-dried (Christ ALPHA 1-2, Germany) and ground to a fine powder. Three replicate samples of each tissue (0.5 g) were collected. The samples were accurately weighed and placed in stoppered conical flasks, and 25 ml of 70% methanol was added. After a second weighing, the samples were ultrasonicated (180 W power, 40 kHz frequency) for 40 min. They were allowed to cool and re-weighed before compensating for any weight loss using 70% methanol and thorough shaking. The resulting mixture was extracted three times with ethyl acetate, and the extracts were combined and concentrated. The concentrate was dissolved in methanol and diluted to a final volume of 10 ml. The supernatant was filtered through a 0.22-μm microporous membrane and subjected to ultra-HPLC (Agilent) to determine the content of CuIIa, CuIIb, and free oleanolic acid. An extract obtained using 70% methanol as described above was concentrated after centrifugation. Reflux was performed with 25 ml of 10% sulfuric acid in a water bath, maintaining slight boiling for 2 h. After cooling overnight, the mixture was filtered and the precipitate washed with water until neutral drying occurred. The dried precipitate was dissolved in methanol and transferred to a 10-ml volumetric flask for dilution up to the mark. An appropriate amount of solution was filtered through a 0.22-μm microporous membrane, and the resulting filtrate was used as the test solution for determining total oleanolic acid.

Content analysis was performed using an Agilent 1290 Infinity II High Performance UHPLC System with a Phenomenex Kinetex C18 analytical column (4.6 × 100 mm, 2.6 μm) and a temperature maintained at 30°C. For detection of CuIIa and CuIIb, we used a gradient elution of 0.2% phosphoric acid aqueous solution (A) and acetonitrile (B): 0–15 min, 25%–33% B; 15–20 min, 33%–40% B; 20–24 min, 40%–60% B; 24–28 min, 60%–90% B; the detection wavelength was set to 212 nm. To determine oleanolic acid content, we used a gradient elution of 0.2% phosphoric acid aqueous solution (A) and acetonitrile (B): 0–10 min, 40%–69% B; 10–15 min, 69% B; 15–20 min, 69%–80% B; the detection wavelength was set to 201 nm. The flow rate was 0.8 ml/min, and the injection volume was 5 μl. CuIIa, CuIIb, cucurbitacin I, cucurbitacin E, and oleanolic acid standards (purchased from Desite Biotechnology, Chengdu, China) were used for quantification and qualitative analysis (Supplemental Table 10).

### Cloning of *HcSE*, *HcOSC*, *HcCYP450*, *HcOxidoreductase*, and *HcAT* coding sequences

*SE*, *OSC*, *CYP450*, *Oxidoreductase*, and *AT* candidate genes were identified from the *H. chinensis* transcriptome database. The protein-coding gene sequences of candidate genes were obtained from the transcriptome data of *H. chinensis*, and primers were designed for amplification of the *HcSE*, *HcOSC*, *HcCYP450*, *HcOxidoreductase*, and *HcAT* gene sequences (Supplemental Table 11). Coding sequences were amplified with the Phanta Max Super-Fidelity DNA Polymerase Kit (Vazyme Biotech), recovered from the gel, and ligated into expression vectors with the ClonExpress II One Step Cloning Kit (Vazyme Biotech). Finally, the gene sequences were cloned into the vector and transferred to *E. coli* DH5α by thermal excitation for sequencing and verification (Supplemental Table 12).

### Sequence and phylogenetic analyses

The online tool ORF Finder (http://www.ncbi.nlm.nih.gov/gorf) was used to acquire the ORFs and amino acid sequences of *HcSEs*, *HcOSCs*, *HcCYP450s*, and *HcATs*. Protein functional domains were identified through alignment. Multiple sequence alignment was performed using DNAMAN software, and Interpro (www.ebi.ac.uk/Tools/InterProScan) was used for domain identification. Amino acid sequences of OSCs from other species were obtained from the NCBI database and aligned using ClustalW. A phylogenetic tree was constructed in IQ-tree software using the maximum-likelihood method with 1000 bootstrap replicates (Nguyen et al., 2015). To construct a phylogenetic tree of ATs, we downloaded reported ATs of different species from NCBI (Supplemental Table 13).

### Tissue expression patterns and real-time qPCR

The total RNA from five tissues of *H. chinensis* was reverse transcribed for real-time qPCR following the manufacturer’s protocols and principles outlined in the HiScript III RT SuperMix for qPCR (+gDNAwiper) Kit (Vazyme Biotech). Specific primers for real-time qPCR (Supplemental Table 11) were designed using online software (http://www.primer3plus.com/), and gene expression was analyzed on the Applied Biosystems QuantStudio 5 real-time PCR system (Applied Biosystems, New York). The PCR conditions consisted of an initial incubation at 95°C for 3 min, followed by 45 cycles of denaturation at 95°C for 3 s and annealing/extension at 60°C for 30 s, with subsequent melting curve analysis. The 18S rRNA gene was used as the reference for quantification of relative gene expression using the 2^–ΔΔCt^ method; triplicate measurements were obtained from three biological replicates (Livak and Schmittgen, 2001).

### Functional identification in *E. coli*

The complete coding regions of HcSEs were cloned into the maltoprotein-tagged pMal-c2x vector through homologous recombination using the ClonExpress II One Step Cloning Kit (Vazyme Biotech). Following the instructions provided, the N-terminal transmembrane domain of HcCPR1 was truncated at 66 amino acid residues (M1-V66) and ligated into the pET-32a vector as described above. Protein purification and enzymatic detection of HcSE and HcCPR1 were performed following previously established protocols (Song et al., 2019b). HcSE was purified using an MBPTrap HP affinity column (Abbkine, China) and eluted with 10 mM maltose, whereas HcCPR1 was purified using a Ni-NTA agarose affinity column and 80 mM imidazole. All buffer systems used Tris–HCl. Recombinant proteins were quantified by 10% (w/v) sodium dodecyl sulfate–polyacrylamide gel electrophoresis (SDS–PAGE) with BSA as the standard for determination of protein concentration. The enzymatic activity of the HcSEs was determined by adding HcCPR1 to the reaction system to provide hydrogen peroxide for oxygenation of the substrate squalene, resulting in production of either 2,3-oxidosqualene or 2,3:22,23-diepoxysqualene (Laden et al., 2000). The 1-ml enzyme reaction mixture comprised the following components: 40 mM squalene substrate (Sigma-Aldrich), 50 μg recombinant HcSE protein, 50 μg recombinant HcCPR1 protein, 1 mM FAD, 1 mM NADPH, Triton X-100 (1%), and 50 mM Tris–HCl (pH 7.5). A negative control was included without the addition of 50 μg recombinant HcCPR1. After incubation overnight at 25°C, extracts from the enzyme reaction mixtures were subjected to GC–MS analysis.

HcAT protein expression, purification, and enzymatic activity detection were performed following previously established protocols (Zhou et al., 2016b). The HcATs were ligated into the pET32a vector. Positive colonies were cultured in LB medium, and cells were harvested after induction of protein expression with 0.1 mM IPTG at 16°C for 18 h. After cell disruption, recombinant His-tagged HcAT was purified by Ni affinity chromatography in a buffer system (50 mM sodium phosphate [pH 8.0], 500 mM NaCl, 80 mM imidazole). Purified proteins were quantified by SDS–PAGE using BSA as a standard for quantification. The enzyme activity of the HcATs was determined by performing HPLC and LC–MS to analyze the presence of the CuIIa product in the enzyme reaction solution, which contained 40 μg purified HcATs, 400 μM CuIIb substrate, and a buffer consisting of 40 mM acetyl-CoA and 50 mM sodium phosphate (pH 7.5). A negative control without recombinant HcCPR1 was also used (Zhou et al., 2016b). Standards of the substrate CuIIa and the product CuIIb were purchased from BioBioPha.

### Immunoblotting

Immunoblotting was used to detect purified HcCPR1 and HcAT proteins (Xue et al., 2018). The proteins were prepared in loading buffer containing SDS and loaded onto LK202 Omni-PAGE 10%–12% Bis-Tris gels (EpiZyme, China) for electrophoresis. A high-quality wet protein transfer machine (genscript, eBlot-L1, China) was used to transfer the proteins from the gel to the membrane, with parameters set to 17 min and a voltage of 50 V. The membranes were then blocked in PBST-buffered saline supplemented with 0.05% (v/v) Tween 20 and incubated at room temperature with mouse monoclonal His-tag monoclonal antibody (GenStar, China) diluted in a solution containing 5% w/v skimmed milk. After 1 h of incubation in PBST buffer and thorough washing steps, the membranes were incubated with IgG-HRP-conjugated secondary antibody (GenStar). The results were visualized using the StarSignal Plus Chemiluminescence Detection Kit (GenStar) (Tanon 5200, China).

### Functional identification in yeast and metabolite extraction

*HcOSCs* obtained by cloning were ligated into the yeast expression vector pYES2 with the ClonExpress II One Step Cloning Kit. Plasmid extraction was performed following identification of positive colonies via sequencing (Vazyme Biotech). The plasmid was introduced into yeast cells via electroporation, and pYES2 without HcOSC was used as a negative control strain. The transformation procedure followed previously described methods. GIL77 yeast cells were cultured in YPD medium supplemented with ergosterol (20 μg/ml), heme (13 μg/ml), and Tween 80 (5 mg/ml) under shaking conditions (Kushiro et al., 1998). The yeast cells were harvested by centrifugation. After a washing treatment with water, 1 M sorbitol, and 0.1 M lithium acetate dissolved in 1 M sorbitol, 100 μl of cells were transferred to a 0.2-cm electroporation cuvette and supplemented with 1 μg of plasmid DNA. Electroporation was performed using a GenePulser Electroporation System (Bio-Rad) with a voltage of 1.5 kV, resistance of 600 Ω, and capacitance of 25 μF. After the electroporated cells recovered in YPD medium for 1 h, the transformants were screened on solid medium without uracil (SC-Ura) for 2–3 days, and positive yeast strains were identified by sequencing (Dong et al., 2018). Culture of the positive transformants and detection of the HcOSC products were performed as described previously (Zhou et al., 2019). The yeast cell culture was initially shaken in SC-Ura medium containing 2% glucose for 2 days, then switched to SC-Ura medium with 2% galactose for 1 day. The cells were then incubated overnight at pH 7.0 with 2% glucose in a potassium phosphate buffer (0.1 M). Finally, the extracted products from the yeast cells were analyzed by GC–MS. Products were extracted from the yeast cells either by ultrasonic extraction with methanol or by refluxing with 10 ml of 20% KOH and 50% EtOH for 5 min. The supernatant was then extracted three times with petroleum ether. Candidate genes responsible for oxidative modification were cloned into the YCplac33 expression vector (Supplemental Table 9). The expression plasmid was transformed into yeast Cuol01-1 using the lithium acetate method, and the YCplac33 plasmid served as a negative control. For each candidate gene, three single colonies were used and inoculated into their respective screening SC medium for cultivation purposes. After 6 days of growth in a shaker, the products were analyzed by HPLC and LC–MS.

### Purification and structural characterization of HcOSC1, HcOSC5, HcOSC6, 87D20-1, and 87D20-2 products

The HcOSC-expressing GIL77 cells and Cuol01-1-PHc87 cells were cultured and harvested in 10 l each. HcOSCs and Cuol01-1-PHc87 were then obtained through saponification cleavage following previously established protocols (Zhou et al., 2019). The extract was dissolved and separated by silica gel column chromatography (zcx. II, particle size 200–300; Haiyang, Qingdao, China) using a hexane:ethyl acetate solvent system (15:1–1:3, v/v). Compounds were eluted through a step gradient system, and the combined target products were analyzed via TLC. Further purification of the target products was performed using a reverse-phase HPLC semi-preparative column (Agilent Zorbax SB C18, 250 × 9.4 mm, 5 μm) for manual collection of the desired compounds. Cycloartenol (20 mg), isomultiflorenol (15 mg), and Cuol (55 mg), as well as 87D20-1 (48 mg) and 87D20-2 (16 mg) were purified from *S. cerevisiae* cultures expressing HcOSC1, HcOSC5, HcOSC6, and HcCYP87D20, respectively. The purified compounds were analyzed by NMR spectroscopy.

Recombinant yeast strains capable of producing Cuol and 11-carbonyl-20β-hydroxy-Cuol were constructed using the previously described method of yeast engineering through homologous recombination (Wang et al., 2015, 2019). The specific step involved PCR amplification to generate each gene expression cassette in the reconstituted pathway, with recombinant or fusion PCR using homologous sequences shared by these cassettes (Supplemental Figure 17A and 17B and Supplemental Table 11). Fusion fragments obtained through fusion PCR were then co-transformed into yeast strains using the lithium acetate method. Integration into chromosomes was achieved by sharing 40–75 bp of homologous sequences, followed by corresponding nutritional screening to generate yeast transformants.

### Yeast cultivation and metabolite extraction

YPD medium was used for yeast growth culture following established protocols. Transformants were subsequently cultivated on SC solid medium supplemented with the corresponding auxotrophic marker to facilitate selection (Wang et al., 2019). The positive yeast transformants were shaken and cultured, after which seed liquid was acquired and inoculated in YPD medium for 96 h. The resulting fermentation broth was extracted using a 1:1 mixture of methanol and acetone to determine the titers of Cuol, 11-carbonyl-Cuol, and 11-carbonyl-20β-hydroxy-Cuol. Fed-batch fermentation, strain cultivation, batch fermentation cultivation, and feeding procedures were performed according to previously published protocols (Wang et al., 2019). After culturing the engineered yeast in 4 ml of YPD liquid for 24 h, seed medium was obtained and expanded to a volume of 100 ml. The seed medium was aseptically inoculated into a 1 l reactor medium to initiate fermentation. Throughout the fermentation process, culture media were collected at various time intervals for analysis of dry cell weight and cucurbitacin triterpene content.

### Transient expression in *N. benthamiana* leaves

The genes sequences of *HcSE1-2*, *HcSQS*, *HcOSC6*, and *HcCYP87D20* from *H. chinensis* were cloned, along with the *GFP* gene from the pAN580 plasmid and a truncated 417-nucleotide portion of the HMGR gene from *Avena strigosa* (*AstHMGR;* GenBank accession number KY284573). These genes were subcloned into the pEAQ-HT-DEST1 binary vector using the ClonExpress II One Step Cloning Kit (Vazyme Biotech) and then transformed into an *A. tumefaciens* strain. The positive strains were confirmed by sequencing in EHA105 (Shanghai Weidi Biotechnology). *AstHMGR* has previously been reported to promote triterpenoid production (Reed et al., 2017). Following previously described methods, we co-expressed *HcOSC6* with *AstHMGR1* and introduced various strain combinations to evaluate gene interactions (Reed et al., 2017). In brief, the transformed *A. tumefaciens* were cultured in LB medium at 28°C for 24 h, followed by cell harvesting using a solution containing 10 mM MES (2-(N-morpholino)ethanesulfonic acid), 10 mM MgCl_2_, and 100 mM acetosyringone (4′-hydroxy-3′,5′-dimethoxyacetophenone). The harvested cells were then suspended in the same solution to adjust the OD_600_ to 0.8 and incubated in darkness with gentle shaking for 2 h. *A. tumefaciens* carrying *AstHMGR1* and *HcOSC6* genes were infiltrated into *N. benthamian*a leaves at week 5 using a modified vacuum infiltration method, and leaf samples were collected after 5 days for triterpenoid analysis (Reed et al., 2017).

### Co-expression network analysis

Fifteen transcriptome datasets from *H. chinensis* were analyzed using the WGCNA package (v.1.47) in R (v.3.2.2). After filtering genes, 19 271 genes were used for WGCNA, and their expression values were imported to construct co-expression networks. The automatic network build function block with default settings was used to obtain modules, which were visualized using Cytoscape_3.3.7 for analysis of gene co-expression relationships (Otasek et al., 2019).

### TLC and GC–MS analysis of extracts

For TLC analysis of target triterpenoids, yeast extract or tobacco extract was dissolved in 1 ml of ethyl acetate. The sample was loaded onto a TLC silica gel plate (Qingdao Haixiang, China) using a capillary tube. The developing solution consisted of n-hexane:ethyl acetate or petroleum ether:ethyl acetate at ratios ranging from 15:1 to 1:3. After development, the plate was dyed with a solution containing 10% sulfuric acid and ethanol, then heated to observe the color development of cucurbitadienol, ergosterol, 11-carbonyl-Cuol, and 11-carbonyl-20β-hydroxy-Cuol. Extracted samples were resuspended in 200 μl extraction solvent, and aliquots of 50 μl were dried under nitrogen gas. Dried aliquots were derivatized with 50 μl of trimethylsilyl (Sigma-Aldrich) and transferred to glass inserts within glass autosampler vials for GC–MS analysis using an Agilent 7890B GC system as described previously (Hodgson et al., 2019). In brief, a 1-μl sample (inlet temperature of 250°C) was injected in pulsed no-spigot mode (pulse pressure of 30 psi), using a program with an initial oven temperature of 170°C for 2 min, followed by a ramp to 300°C at a rate of 20°C/min and a hold at 300°C for an additional 11.5 min. Detection was performed in scan mode (60–800 mass units) with a solvent delay set to 8 min and adjusted to precisely match the retention time. Data acquisition and analysis were performed using MassHunter Workstation software from Agilent Technologies. Quantitative and qualitative assessments were performed using squalene, 2,3-squalene oxide, 2,3:22,23-dioxasqualene, and ergosterol standards purchased from Sigma-Aldrich for qualitative purposes, as well as Cuol standards obtained by purification (Supplemental Table 10).

### HPLC and HPLC/ESIMS analysis of extracts

The analytical method used to detect the target product in the HcAT reactions involved quantifying the contents of CuIIa and CuIIb in the plant sample as described above, with 0.01% formic acid replacing 0.2% phosphoric acid. To detect 11-carbonyl-Cuol, 11-carbonyl-20β-hydroxy-Cuol, and 11-carbonyl-2β,20β-dihydroxy-Cuol, we used a gradient elution of 0.01% formic acid aqueous solution (A) and acetonitrile (B): 0–12 min, 70%–73% B; 12–16 min, 73%–94% B; 16–26 min, 94% B. The detection wavelength was set to 210 nm. In addition, the extracts were analyzed using an Agilent 1290 UPLC/6540 Q-TOF system (Agilent Technologies) equipped with an Agilent Poroshell 120 EC C18 analytical column (4.6 × 100 mm, 2.7 μm). The ion source operated in positive ion mode with voltage settings as follows: 3500 V voltage setting; 135 V fragmentation voltage; 60 V cone voltage; 750 V RF voltage. The scanning range spanned from 100 to 1000 *m/z*. For quantification and qualitative purposes, we used a cucurbitadienol standard along with purified confirmed samples of 11-carbonyl-Cuol and 11-carbonyl-20β-hydroxy-Cuol (Supplemental Table 10).

### NMR analysis

The purified compounds were analyzed using ^1^H-NMR and ^13^C-NMR spectroscopy on Bruker AV-600 MHz and 800 MHz spectrometers in CDCl_3_ or C_5_D_5_N solutions at the respective frequencies. Chemical shifts were recorded in ppm and referenced to the residual solvent peak. Multiplicities were denoted as follows: s, singlet; d, doublet; dd, doublet of doublets; dt, doublet of triplets; t, triplet; q, quartet; m, multiplet; br, broad; appt, apparent. Coupling constants are reported in Hertz.

## Data and code availability

The transcriptome reported in this paper has been deposited in the Medicinal Plants multi-Omics Database, http://medicinalplants.ynau.edu.cn/transcriptomics/213. The raw transcriptome sequencing data have been deposited in the NCBI database under project number PRJNA879990. The sequences functionally characterized in this paper have been deposited in the GenBank database, and their accession numbers are listed in Supplemental Table 12.

## Funding

This work was supported by the 10.13039/501100018531Major Science and Technology Projects in Yunnan Province (2019ZF011-1), the Fundamental Research Project of Yunnan (202101AS070037), the Science and Technology Innovation team of Yunnan (202105AE160011), the Major Science and Technique Programs in Yunnan Province (202102AE090042), the Yunnan Characteristic Plant Extraction Laboratory (2022YKZY001), the First Projects of Science and Technology Plan in the Biomedical field in 2021 (202102AA310048), and the 10.13039/501100001809National Natural Science Foundation of China (grant nos. 81960691 and 82160727).

## Author contributions

B.H., G.Z., and S.Y. conceived the study. G.C., Z.G., and Y.S. contributed equally to this work. G.C., Z.G., Y.S., L.Q., S.D., Y.L., S.H., B.L., and X.F. performed the experiments. G.C., Z.G., Y.S., B.H., and G.Z. designed the experiments. C.Y. and Z.L. were responsible for purification of the compounds and NMR data analysis. Y.S. photographed the plants. G.L., Y.Z., B.N., and G.X. analyzed the data. G.C., Y.W., B.H., and G.Z. drafted the manuscript. B.H., G.Z., and S.Y. reviewed and edited the manuscript. All authors contributed to the article and approved the submitted version.

## Supplemental information

Document S1. Supplemental Figures 1–23 and supplemental Tables 1–13

Document S2. Article plus supplemental information

## References

[bib1] Almeida A., Dong L., Thorsen T.H., Raadam M.H., Khakimov B., Carreno-Quintero N., Kampranis S.C., Bak S. (2022). Metabolic engineering of cucurbitacins in Cucurbita pepo hairy roots. Front. Plant Sci..

[bib2] Blazeck J., Garg R., Reed B., Alper H.S. (2012). Controlling promoter strength and regulation in Saccharomyces cerevisiae using synthetic hybrid promoters. Biotechnol. Bioeng..

[bib3] Cao Z., Li S., Lv J., Gao H., Chen G., Awakawa T., Abe I., Yao X., Hu D. (2019). Biosynthesis of clinically used antibiotic fusidic acid and identification of two short-chain dehydrogenase/reductases with converse stereoselectivity. Acta Pharm. Sin. B.

[bib4] Chen J.C., Chiu M.H., Nie R.L., Cordell G.A., Qiu S.X. (2005). Cucurbitacins and cucurbitane glycosides: structures and biological activities. Nat. Prod. Rep..

[bib5] Chen R., Yang S., Zhang L., Zhou Y.J. (2020). Advanced Strategies for Production of Natural Products in Yeast. iScience.

[bib6] Cho H.J., Tanaka S., Fukui H., Tabata M. (1992). Formation of bryonolic acid in cucurbitaceous plants and their cell cultures. Phytochemistry.

[bib7] Choi H.S., Han J.Y., Cheong E.J., Choi Y.E. (2022). Characterization of a pentacyclic triterpene acetyltransferase involved in the biosynthesis of taraxasterol and ψ-taraxasterol acetates in lettuce. Front. Plant Sci..

[bib8] Christ B., Xu C., Xu M., Li F.S., Wada N., Mitchell A.J., Han X.L., Wen M.L., Fujita M., Weng J.K. (2019). Repeated evolution of cytochrome P450-mediated spiroketal steroid biosynthesis in plants. Nat. Commun..

[bib9] Chu L.L., Huy N.Q., Tung N.H. (2023). Microorganisms for Ginsenosides Biosynthesis: Recent Progress, Challenges, and Perspectives. Molecules.

[bib10] Da Silva N.A., Srikrishnan S. (2012). Introduction and expression of genes for metabolic engineering applications in Saccharomyces cerevisiae. FEMS Yeast Res..

[bib11] Dai Z., Wang B., Liu Y., Shi M., Wang D., Zhang X., Liu T., Huang L., Zhang X. (2014). Producing aglycons of ginsenosides in bakers' yeast. Sci. Rep..

[bib12] Dong L., Almeida A., Pollier J., Khakimov B., Bassard J.E., Miettinen K., Stærk D., Mehran R., Olsen C.E., Motawia M.S. (2021). An Independent Evolutionary Origin for Insect Deterrent Cucurbitacins in Iberis amara. Mol. Biol. Evol..

[bib13] Dong L., Pollier J., Bassard J.E., Ntallas G., Almeida A., Lazaridi E., Khakimov B., Arendt P., de Oliveira L.S., Lota F. (2018). Co-expression of squalene epoxidases with triterpene cyclases boosts production of triterpenoids in plants and yeast. Metab. Eng..

[bib14] Gibson D.G., Young L., Chuang R.-Y., Venter J.C., Hutchison C.A., Smith H.O. (2009). Enzymatic assembly of DNA molecules up to several hundred kilobases. Nat. Methods.

[bib15] Hayashi H., Huang P., Inoue K., Hiraoka N., Ikeshiro Y., Yazaki K., Tanaka S., Kushiro T., Shibuya M., Ebizuka Y. (2001). Molecular cloning and characterization of isomultiflorenol synthase, a new triterpene synthase from Luffa cylindrica, involved in biosynthesis of bryonolic acid. Eur. J. Biochem..

[bib16] Hesami M., Pepe M., Baiton A., Jones A.M.P. (2022). Current status and future prospects in cannabinoid production through in vitro culture and synthetic biology. Biotechnol. Adv..

[bib17] Hodgson H., De La Peña R., Stephenson M.J., Thimmappa R., Vincent J.L., Sattely E.S., Osbourn A. (2019). Identification of key enzymes responsible for protolimonoid biosynthesis in plants: Opening the door to azadirachtin production. Proc. Natl. Acad. Sci. USA.

[bib18] Hu B., Zhao X., Wang E., Zhou J., Li J., Chen J., Du G. (2023). Efficient heterologous expression of cytochrome P450 enzymes in microorganisms for the biosynthesis of natural products. Crit. Rev. Biotechnol..

[bib19] Huang A.C., Jiang T., Liu Y.X., Bai Y.C., Reed J., Qu B., Goossens A., Nützmann H.W., Bai Y., Osbourn A. (2019). A specialized metabolic network selectively modulates Arabidopsis root microbiota. Science.

[bib20] Hussain H., Green I.R., Saleem M., Khattak K.F., Irshad M., Ali M. (2019). Cucurbitacins as Anticancer Agents: A Patent Review. Recent Pat. Anti-Cancer Drug Discov..

[bib21] Kushiro T., Shibuya M., Ebizuka Y. (1998). β-Amyrin synthase: cloning of oxidosqualene cyclase that catalyzes the formation of the most popular triterpene among higher plants. Eur. J. Biochem..

[bib22] Laden B.P., Tang Y., Porter T.D. (2000). Cloning, heterologous expression, and enzymological characterization of human squalene monooxygenase. Arch. Biochem. Biophys..

[bib23] Lau W., Sattely E.S. (2015). Six enzymes from mayapple that complete the biosynthetic pathway to the etoposide aglycone. Science.

[bib24] Li M., Ma M., Wu Z., Liang X., Zheng Q., Li D., An T., Wang G. (2023). Advances in the biosynthesis and metabolic engineering of rare ginsenosides. Appl. Microbiol. Biotechnol..

[bib25] Li Y., Chen T., Wang W., Liu H., Yan X., Wu-Zhang K., Qin W., Xie L., Zhang Y., Peng B. (2021). A high-efficiency Agrobacterium-mediated transient expression system in the leaves of Artemisia annua L. Plant Methods.

[bib26] Liu X., Cheng J., Zhang G., Ding W., Duan L., Yang J., Kui L., Cheng X., Ruan J., Fan W. (2018). Engineering yeast for the production of breviscapine by genomic analysis and synthetic biology approaches. Nat. Commun..

[bib27] Livak K.J., Schmittgen T.D. (2001). Analysis of relative gene expression data using real-time quantitative PCR and the 2− ΔΔCT method. methods.

[bib28] Lv J.M., Hu D., Gao H., Kushiro T., Awakawa T., Chen G.D., Wang C.X., Abe I., Yao X.S. (2017). Biosynthesis of helvolic acid and identification of an unusual C-4-demethylation process distinct from sterol biosynthesis. Nat. Commun..

[bib29] Marsian J., Lomonossoff G.P. (2016). Molecular pharming - VLPs made in plants. Curr. Opin. Biotechnol..

[bib30] Mitsuguchi H., Seshime Y., Fujii I., Shibuya M., Ebizuka Y., Kushiro T. (2009). Biosynthesis of steroidal antibiotic fusidanes: functional analysis of oxidosqualene cyclase and subsequent tailoring enzymes from Aspergillus fumigatus. J. Am. Chem. Soc..

[bib31] Moses T., Pollier J., Faizal A., Apers S., Pieters L., Thevelein J.M., Geelen D., Goossens A. (2015). Unraveling the triterpenoid saponin biosynthesis of the African shrub Maesa lanceolata. Mol. Plant.

[bib32] Nett R.S., Sattely E.S. (2021). Total Biosynthesis of the Tubulin-Binding Alkaloid Colchicine. J. Am. Chem. Soc..

[bib33] Nett R.S., Lau W., Sattely E.S. (2020). Discovery and engineering of colchicine alkaloid biosynthesis. Nature.

[bib34] Nguyen L.-T., Schmidt H.A., Von Haeseler A., Minh B.Q. (2015). IQ-TREE: a fast and effective stochastic algorithm for estimating maximum-likelihood phylogenies. Mol. Biol. Evol..

[bib35] Nomura M., Nogi Y., Oakes M. (2013).

[bib36] Okamoto S., Yu F., Harada H., Okajima T., Hattan J.i., Misawa N., Utsumi R. (2011). A short-chain dehydrogenase involved in terpene metabolism from Zingiber zerumbet. FEBS J..

[bib37] Otasek D., Morris J.H., Bouças J., Pico A.R., Demchak B. (2019). Cytoscape automation: empowering workflow-based network analysis. Genome Biol..

[bib38] Qiao J., Luo Z., Cui S., Zhao H., Tang Q., Mo C., Ma X., Ding Z. (2019). Modification of isoprene synthesis to enable production of curcurbitadienol synthesis in Saccharomyces cerevisiae. J. Ind. Microbiol. Biotechnol..

[bib39] Reed J., Osbourn A. (2018). Engineering terpenoid production through transient expression in Nicotiana benthamiana. Plant Cell Rep..

[bib40] Reed J., Stephenson M.J., Miettinen K., Brouwer B., Leveau A., Brett P., Goss R.J.M., Goossens A., O'Connell M.A., Osbourn A. (2017). A translational synthetic biology platform for rapid access to gram-scale quantities of novel drug-like molecules. Metab. Eng..

[bib41] Ren Y., Kinghorn A.D. (2019). Natural Product Triterpenoids and Their Semi-Synthetic Derivatives with Potential Anticancer Activity. Planta Med..

[bib42] Sawai S., Saito K. (2011). Triterpenoid biosynthesis and engineering in plants. Front. Plant Sci..

[bib43] Seki H., Tamura K., Muranaka T. (2015). P450s and UGTs: key players in the structural diversity of triterpenoid saponins. Plant Cell Physiol..

[bib44] Shang Y., Ma Y., Zhou Y., Zhang H., Duan L., Chen H., Zeng J., Zhou Q., Wang S., Gu W. (2014). Plant science. Biosynthesis, regulation, and domestication of bitterness in cucumber. Science.

[bib45] Song W., Yan S., Li Y., Feng S., Zhang J.J., Li J.R. (2019). Functional characterization of squalene epoxidase and NADPH-cytochrome P450 reductase in Dioscorea zingiberensis. Biochem. Biophys. Res. Commun..

[bib46] Song W., Yan S., Li Y., Feng S., Zhang J.-j., Li J.-r. (2019). Functional characterization of squalene epoxidase and NADPH-cytochrome P450 reductase in Dioscorea zingiberensis. Biochem. Biophys. Res. Commun..

[bib47] Takase S., Kera K., Hirao Y., Hosouchi T., Kotake Y., Nagashima Y., Mannen K., Suzuki H., Kushiro T. (2019). Identification of triterpene biosynthetic genes from Momordica charantia using RNA-seq analysis. Biosci. Biotechnol. Biochem..

[bib48] Takase S., Kera K., Nagashima Y., Mannen K., Hosouchi T., Shinpo S., Kawashima M., Kotake Y., Yamada H., Saga Y. (2019). Allylic hydroxylation of triterpenoids by a plant cytochrome P450 triggers key chemical transformations that produce a variety of bitter compounds. J. Biol. Chem..

[bib49] Tang H., Wu Y., Deng J., Chen N., Zheng Z., Wei Y., Luo X., Keasling J.D. (2020). Promoter Architecture and Promoter Engineering in Saccharomyces cerevisiae. Metabolites.

[bib50] Tu L., Su P., Zhang Z., Gao L., Wang J., Hu T., Zhou J., Zhang Y., Zhao Y., Liu Y. (2020). Genome of Tripterygium wilfordii and identification of cytochrome P450 involved in triptolide biosynthesis. Nat. Commun..

[bib51] Wang P., Wei W., Ye W., Li X., Zhao W., Yang C., Li C., Yan X., Zhou Z. (2019). Synthesizing ginsenoside Rh2 in Saccharomyces cerevisiae cell factory at high-efficiency. Cell Discov..

[bib52] Wang P., Wei Y., Fan Y., Liu Q., Wei W., Yang C., Zhang L., Zhao G., Yue J., Yan X., Zhou Z. (2015). Production of bioactive ginsenosides Rh2 and Rg3 by metabolically engineered yeasts. Metab. Eng..

[bib53] Xue Z., Tan Z., Huang A., Zhou Y., Sun J., Wang X., Thimmappa R.B., Stephenson M.J., Osbourn A., Qi X. (2018). Identification of key amino acid residues determining product specificity of 2,3-oxidosqualene cyclase in Oryza species. New Phytol..

[bib54] Zhang Y., Nowak G., Reed D.W., Covello P.S. (2011). The production of artemisinin precursors in tobacco. Plant Biotechnol. J..

[bib55] Zhang Y., Zeng Y., An Z., Lian D., Xiao H., Wang R., Zhang R., Zhai F., Liu H. (2022). Comparative transcriptome analysis and identification of candidate genes involved in cucurbitacin IIa biosynthesis in Hemsleya macrosperma. Plant Physiol. Biochem..

[bib56] Zhong Y., Xun W., Wang X., Tian S., Zhang Y., Li D., Zhou Y., Qin Y., Zhang B., Zhao G. (2022). Root-secreted bitter triterpene modulates the rhizosphere microbiota to improve plant fitness. Nat. Plants.

[bib57] Zhou J., Hu T., Gao L., Su P., Zhang Y., Zhao Y., Chen S., Tu L., Song Y., Wang X. (2019). Friedelane-type triterpene cyclase in celastrol biosynthesis from Tripterygium wilfordii and its application for triterpenes biosynthesis in yeast. New Phytol..

[bib58] Zhou Y., Ma Y., Zeng J., Duan L., Xue X., Wang H., Lin T., Liu Z., Zeng K., Zhong Y. (2016). Convergence and divergence of bitterness biosynthesis and regulation in Cucurbitaceae. Nat. Plants.

[bib59] Zhou Y., Ma Y., Zeng J., Duan L., Xue X., Wang H., Lin T., Liu Z., Zeng K., Zhong Y. (2016). Convergence and divergence of bitterness biosynthesis and regulation in Cucurbitaceae. Nat. Plants.

[bib60] Zhu M., Wang C., Sun W., Zhou A., Wang Y., Zhang G., Zhou X., Huo Y., Li C. (2018). Boosting 11-oxo-beta-amyrin and glycyrrhetinic acid synthesis in Saccharomyces cerevisiae via pairing novel oxidation and reduction system from legume plants. Metab. Eng..

