# Supplementary material for: Biosynthetic pathway of prescription cucurbitacin IIa and high-level production of key triterpenoid intermediates in engineered yeast and tobacco
Source: Plant Commun. 2024 Feb 29;5(6):100835. doi: 10.1016/j.xplc.2024.100835 (PMC11211238; doi:10.1016/j.xplc.2024.100835)
Supplement: Document S1. Supplemental Figures 1–23 and supplemental Tables 1–13 [file mmc1.pdf]

Plant Communications, Volume *5*

Supplemental information

Biosynthetic pathway of prescription cucurbitacin IIa and high-level production of key triterpenoid intermediates in engineered yeast and tobacco

Geng Chen, Zhaokuan Guo, Yanyu Shu, Yan Zhao, Lei Qiu, Shaofeng Duan, Yuan Lin, Simei He, Xiaobo Li, Xiaolin Feng, Guisheng Xiang, Bo Nian, Yina Wang, Zhiyuan Li, Chongkang Yang, Yang Shi, Yingchun Lu, Guanze Liu, Shengchao Yang, Guanghui Zhang, and Bing Hao

Supplemental information

Biosynthetic pathway of prescription cucurbitacin IIa and high-level production of key triterpenoid intermediates in engineered yeast and tobacco

Geng Chen, Zhaokuan Guo, Yanyu Shu, Yan Zhao, Lei Qiu, Shaofeng Duan, Yuan Lin, Simei He, Xiaobo Li, Xiaolin Feng, Guisheng Xiang, Bo Nian, Yina Wang, Zhiyuan Li, Chongkang Yang, Yang Shi, Yingchun Lu, Guanze Liu, Shengchao Yang, Guanghui Zhang, and Bing Hao

Biosynthetic pathway of prescription cucurbitacin IIa and high-level production of key triterpenoid intermediates in engineered yeast and tobacco

Geng Chen [1,2,3], Zhaokuan Guo [1,2,3], Yanyu Shu [1,2,3], Yan Zhao [1,2], Lei Qiu [1,2], Shaofeng Duan [1,2], Yuan Lin [1,2], Simei He [1,2], Xiaobo Li [1,2], Xiaolin Feng [1,2], Guisheng Xiang [1,2], Bo Nian [1], Yina Wang [1,2], Zhiyuan Li [1,2], Chongkang yang [1], Yang Shi [1], Yingchun Lu[1,2], Guanze Liu[1,2], Shengchao Yang [1,2], Guanghui Zhang* [1,2], Bing Hao* [1,2]

1. State Key Laboratory of Conservation and Utilization of Bio-resources in Yunnan, The Key Laboratory of Medicinal Plant Biology of Yunnan Province, National & Local Joint Engineering Research Center on Germplasms Innovation & Utilization of Chinese Medicinal Materials in Southwest China, Yunnan Agricultural University, Kunming, 650201, China.

2. Yunnan Characteristic Plant Extraction Laboratory, Kunming, Yunnan 650106, China.

3. These authors contributed equally to this article.

*Corresponding author: Bing Hao, E-mail: Bing.Hao@hotmail.com

Guang-hui Zhang, E-mail: zgh73107310@163.com

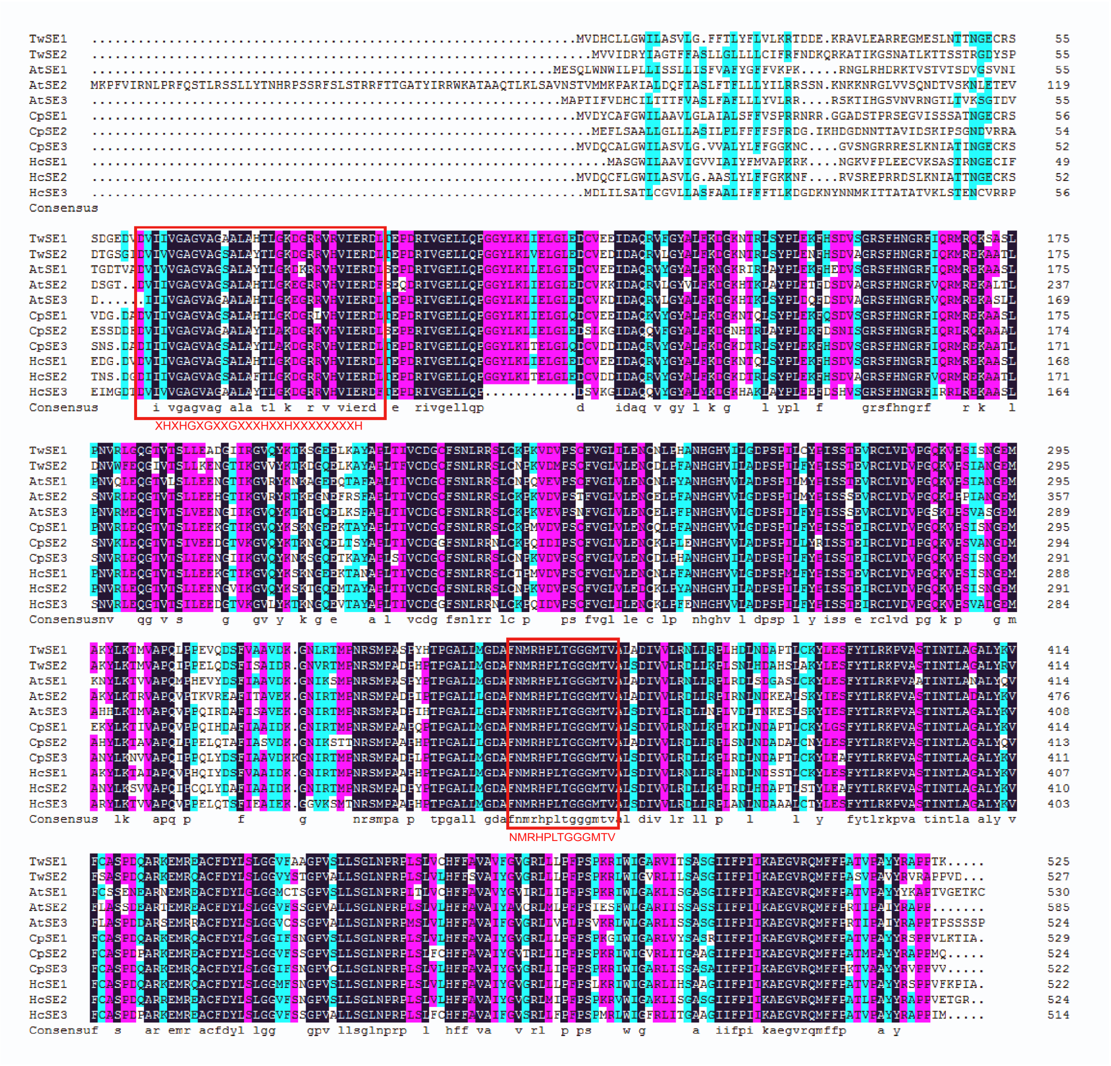

Supplemental Figure 1 Deduced amino-acid sequence alignment of three SEs from *H.chinensis*. Conserved an NAD (P)-binding site and NMRHPLTGGGMTV regions are in the red box.

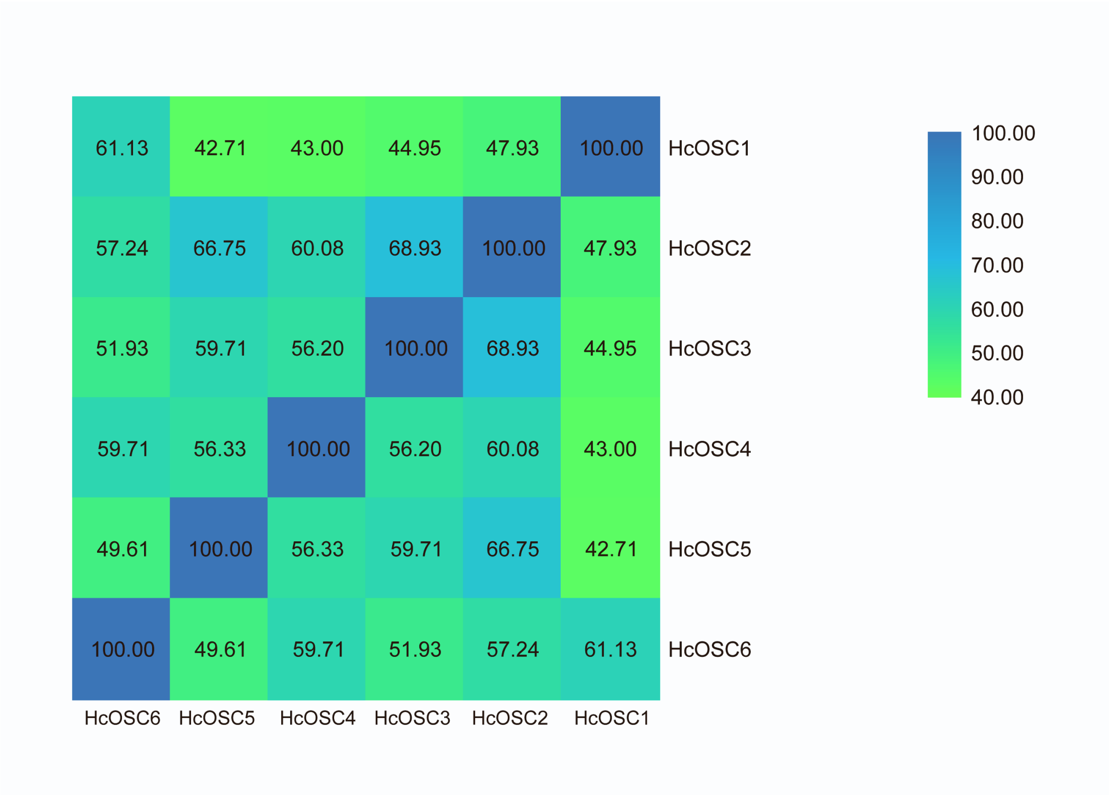

Supplemental Figure 2 Amino acid sequence identity: comparison among six OSCs cloned from *H.chinensis*.

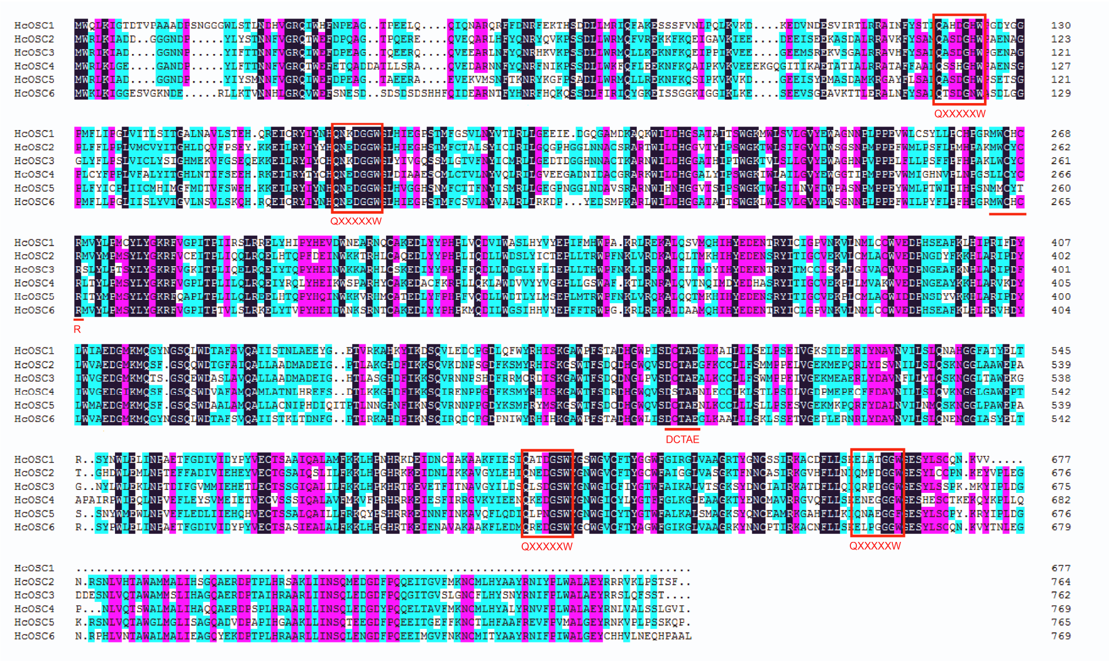

Supplemental Figure 3 Deduced amino-acid sequence alignment of six OSCs from *H.chinensis*. Putative DCTAE substrate- binding motif, conserved QXXXXXW and MWCYCR regions are in the red box.

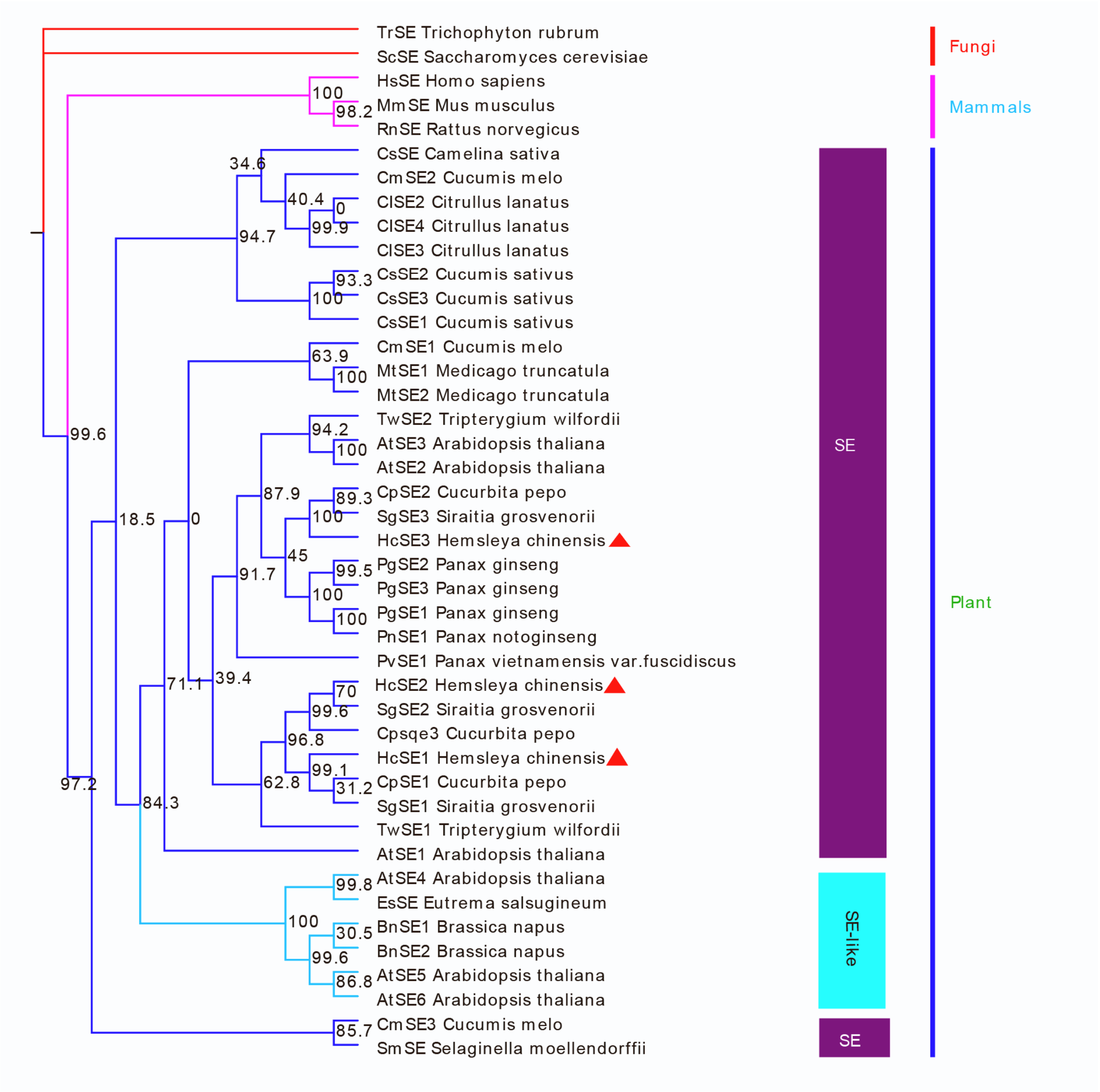

Supplemental Figure 4 Phylogenetic analysis of selected SEs from different species. The maximum-likelihood (ML) phylogenetic analysis was built by IQ-tree software, and the bootstrap confidence values were obtained based on 1000 replicates. Two fungi SE sequences used as an out group. The HcSE1–3 was classified into the SE group, which indicated that these SEs could catalyze squalene to produce 2,3-oxidosqualene.

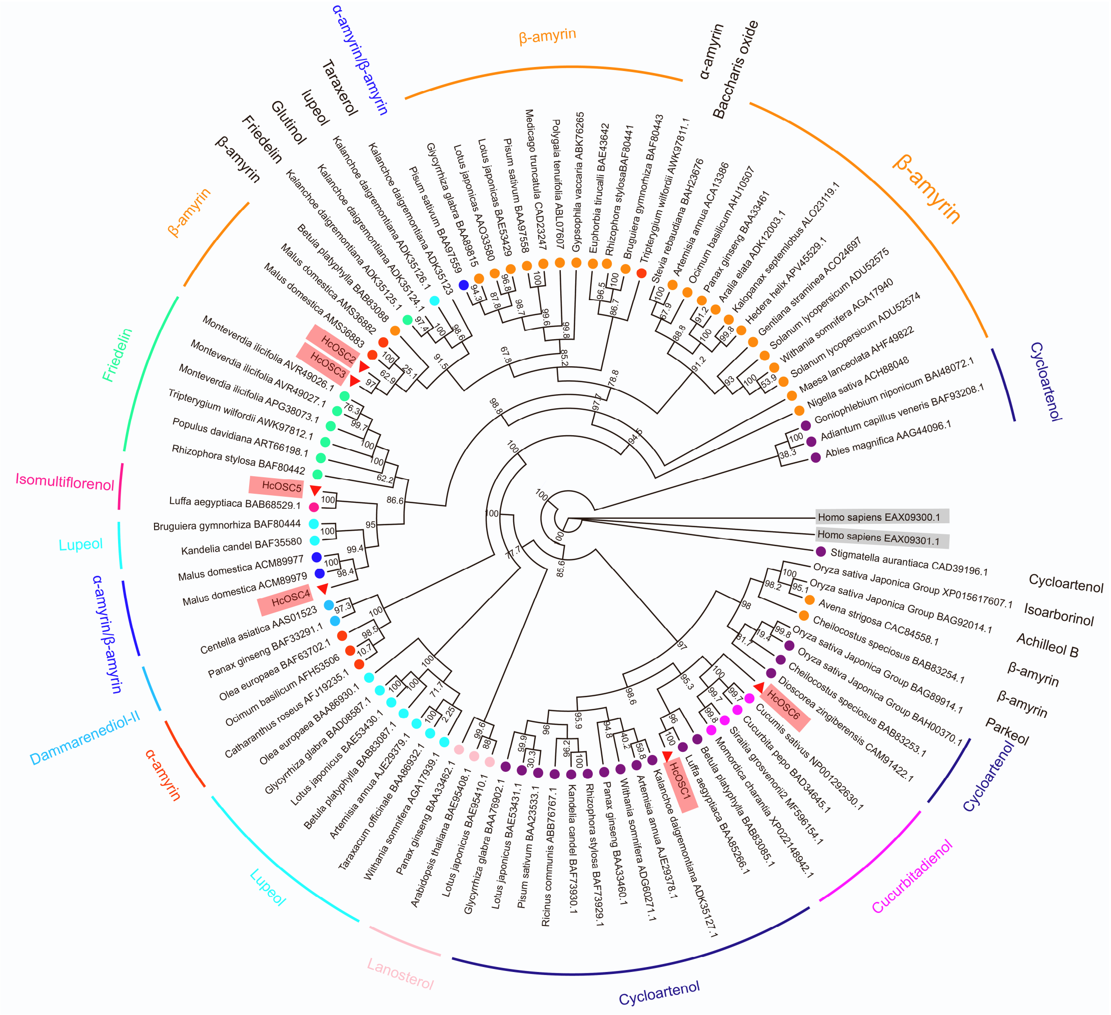

Supplemental Figure 5 Phylogenetic analysis of OSCs identified in transcriptome of *H.chinensis*. The maximum-likelihood (ML) phylogenetic analysis was built by IQ-tree software, and the bootstrap confidence values were obtained based on 1000 replicates. HcOSC1 clustered within the clade of previously characterized cycloartenol synthases; HcOSC2, HcOSC3 and HcOSC4 clustered in the known characterized β-amyrin synthases; HcOSC5 clustered with previously characterized isomultiflorenol synthases from Luffa aegyptiaca; and *HcOSC6* clustered with previously characterized cuol synthases from Cucurbitaceae.

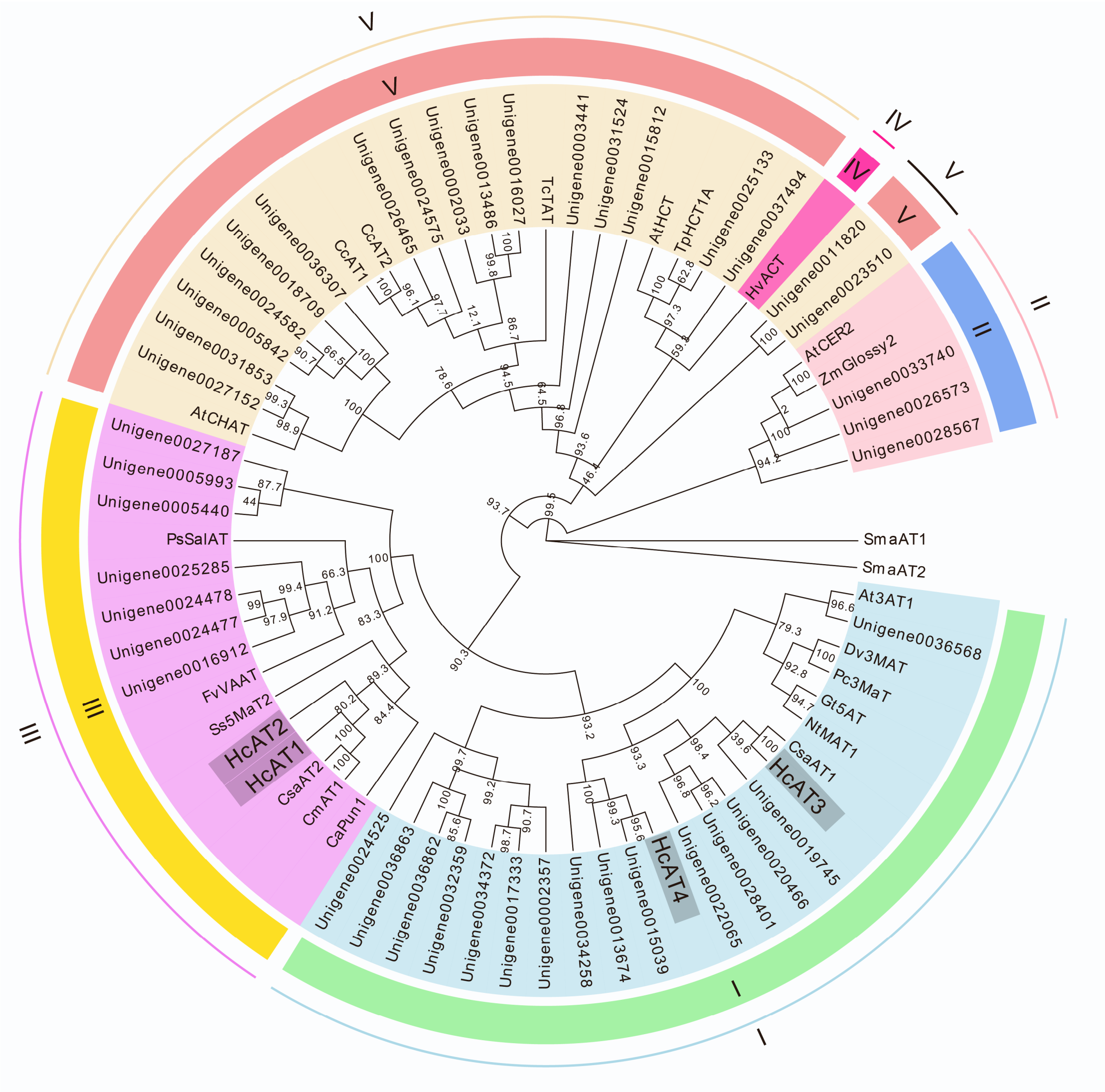

Supplemental Figure 6 Phylogenetic analysis of BAHD-ATs identified in transcriptome of *H.chinensis*. The maximum-likelihood (ML) phylogenies tree was built by IQ-tree software use the 47 montbretia BAHD-AT sequences identified in the *H. chinensis* transcriptome together with selected BAHD-ATs from other plant species, and the bootstrap confidence values were obtained based on 1000 replicates. Alignments and phylogeny were used to cluster Hemsleya chinensis BAHD-ATs with known BAHD-AT clades. Hemsleya chinensis BAHD-ATs characterized in this work are in bold; all other Hemsleya chinensis BAHD-ATs are labelled with their unigene numbers. Clades are labelled with numbers outside the circle. Bootstrap values are shown beside each node. Accession numbers are provided in Supplemental Table 5. With the majority falling into clade I and V. HcAT3 and HcAT4 were clustered in clade I with specific AT (Csa5G639480), which was previously a negative control in cucumber leaves (Shang et al., 2014). HcAT1 and HcAT2 were clustered in clade III with previously characterized CsaAT2 (Csa6G088700) from C. sativus and CmAT1 (Melo3C022373) from *C. melo*.

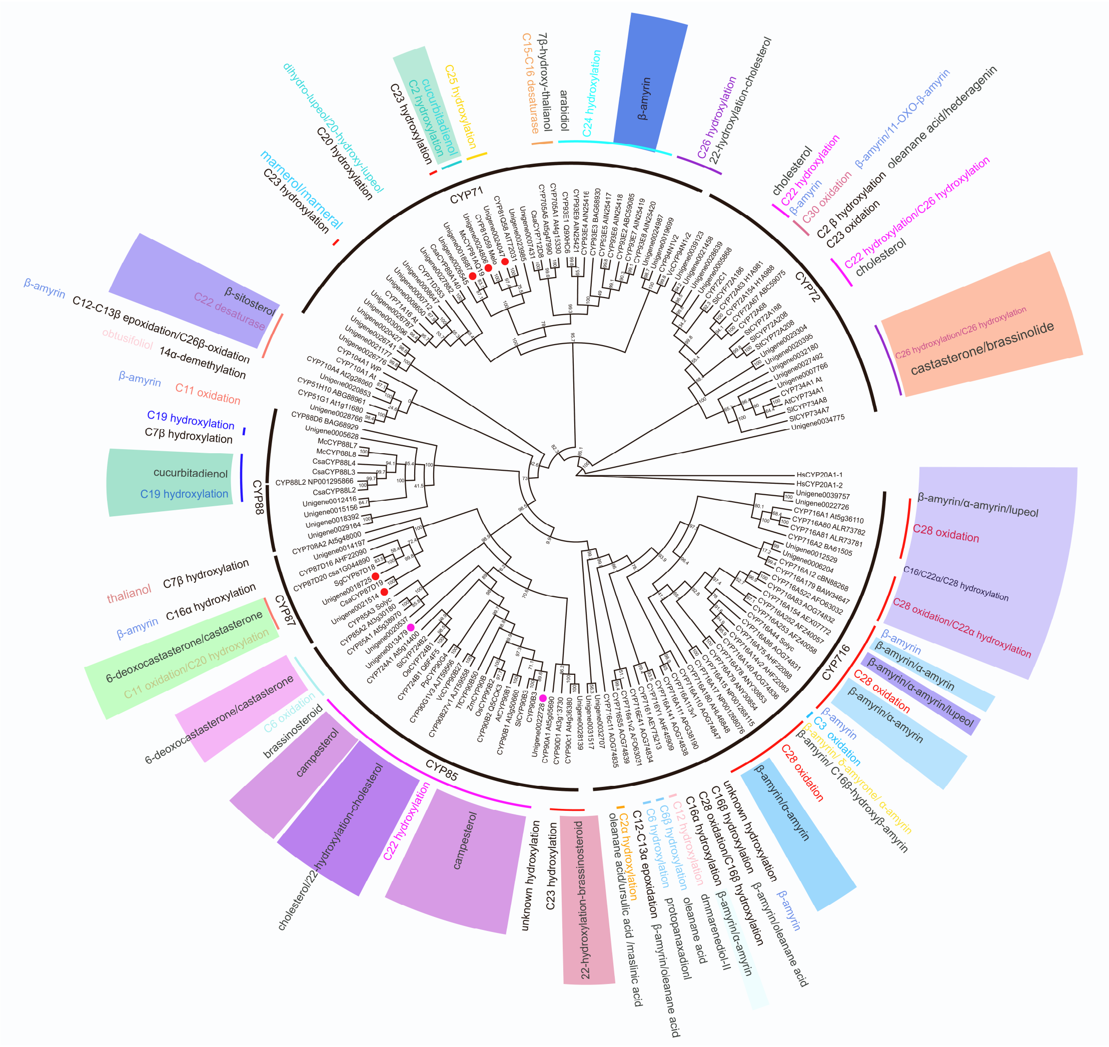

Supplemental Figure 7 phylogenetic analysis of putative CYP450s involved in the triterpenoid biosynthesis. The maximum-likelihood (ML) phylogenetic analysis was built by IQ-tree software, and the bootstrap confidence values were obtained based on 1000 replicates. phylogenetic analysis of putative CYP450s with CYP450s from other cucurbitaceae species. Putative unigenes with red circular in the tree were annotated as important CYP450s related to oxidation of curcurbitacin backbones. 4 candidate genes belonged to P450 families, including CYP71, CYP85, CYP88 and CYP87, clustered on different clades with known P450s were involved in cucurbitacin biosynthesis.

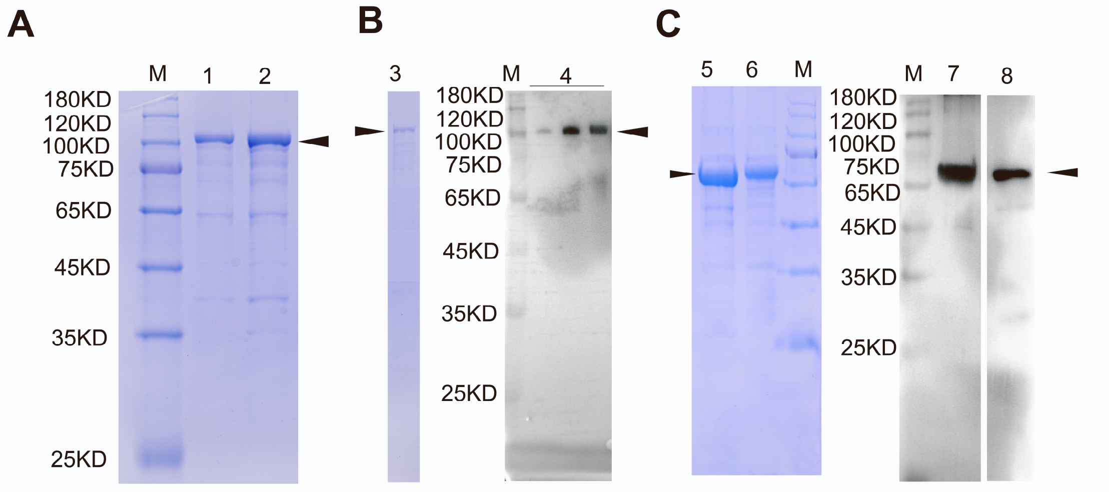

Supplemental Figure 8 SDS–PAGE of the HcSEs, HcCPR and HcATs.

(A) lane M, Precision Protein Standard (Bio-Rad) with molecular weights given in kilodalton; Lane 1-2, purified *Hc*SE1, HcSE2 after the gel filtration step, respectively, (B) Lane 3, purified HcCPR1 after the gel filtration step; Lane 4, Corresponding Western blot of the same protein sample with antibody directed against the his-tag, (C) Lane 5-6, purified HcAT1, HcAT2 after the gel filtration step, respectively; Lane 7-8, Corresponding Western blot of the same protein sample with antibody directed against the his-tag.

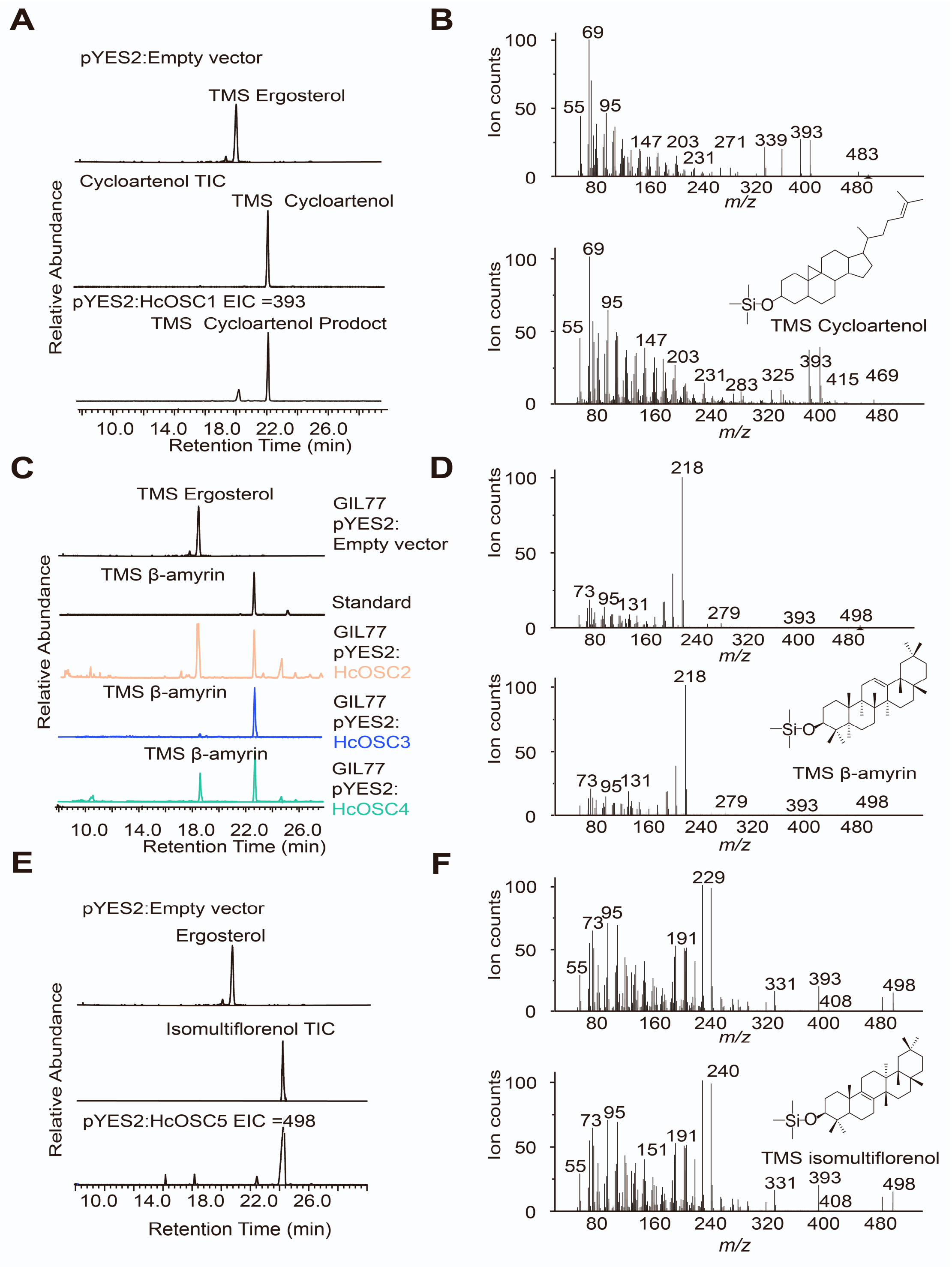

Supplemental Figure 9 Identification and characterization of HcOSCs from *Hemsleya chinensis*.

(A) GC-MS analysis of the products in yeast strains containing the HcOSC1 expression plasmids and empty vector, (B) GC-MS analysis of the products in yeast strains containing the HcOSC2-4 expression plasmids and empty vector, (C) GC-MS analysis of the products in yeast strains containing the HcOSC5 expression plasmids and empty vector.

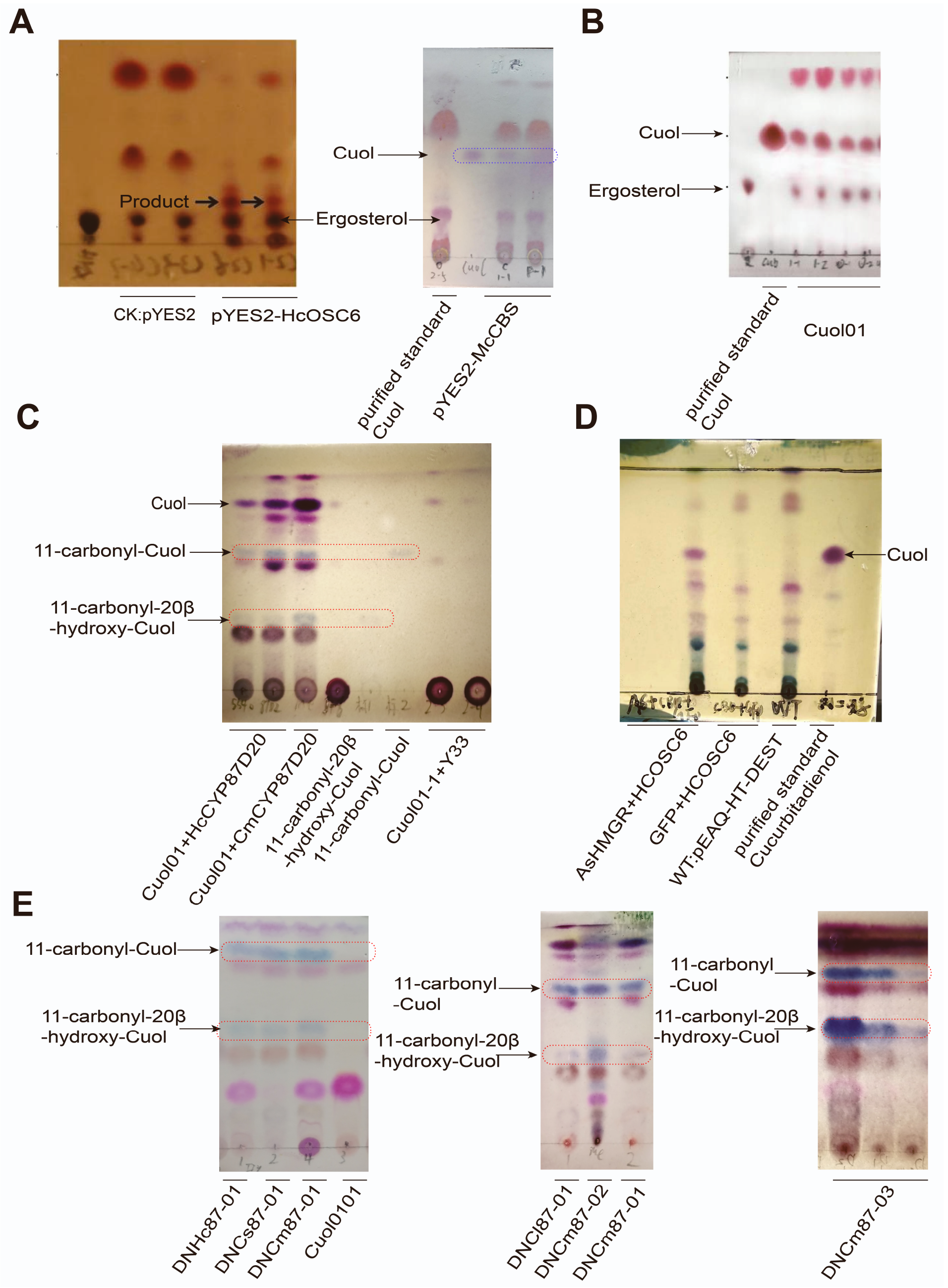

Supplemental Figure 10 Analysis of Triterpenes in Yeast and *N. benthamiana* Extracts by Thin-Layer Chromatography (TLC).

(A) TLC analysis of catalytic products in yeast GIL77 by pYES2-HcOSC6 and pYES2-McCBS. **(B)** TLC analysis of catalytic products in yeast cells by constructing HcOSC6-overexpressing chassis(Cuol01-1). **(C)** TLC analysis catalytic products in yeast Cuol01-1 by Y33-HcCYP87D20 and Y33-CmCYP87D20, **(D)** TLC analysis of catalytic products transiently co-expressed in *N. benthamiana* by HcOSC6 and AstHMGR. **(E)** TLC analysis of catalytic products in High-level production of 11-carbonyl-20β-hydroxy-Cuol in engineering yeast cells.

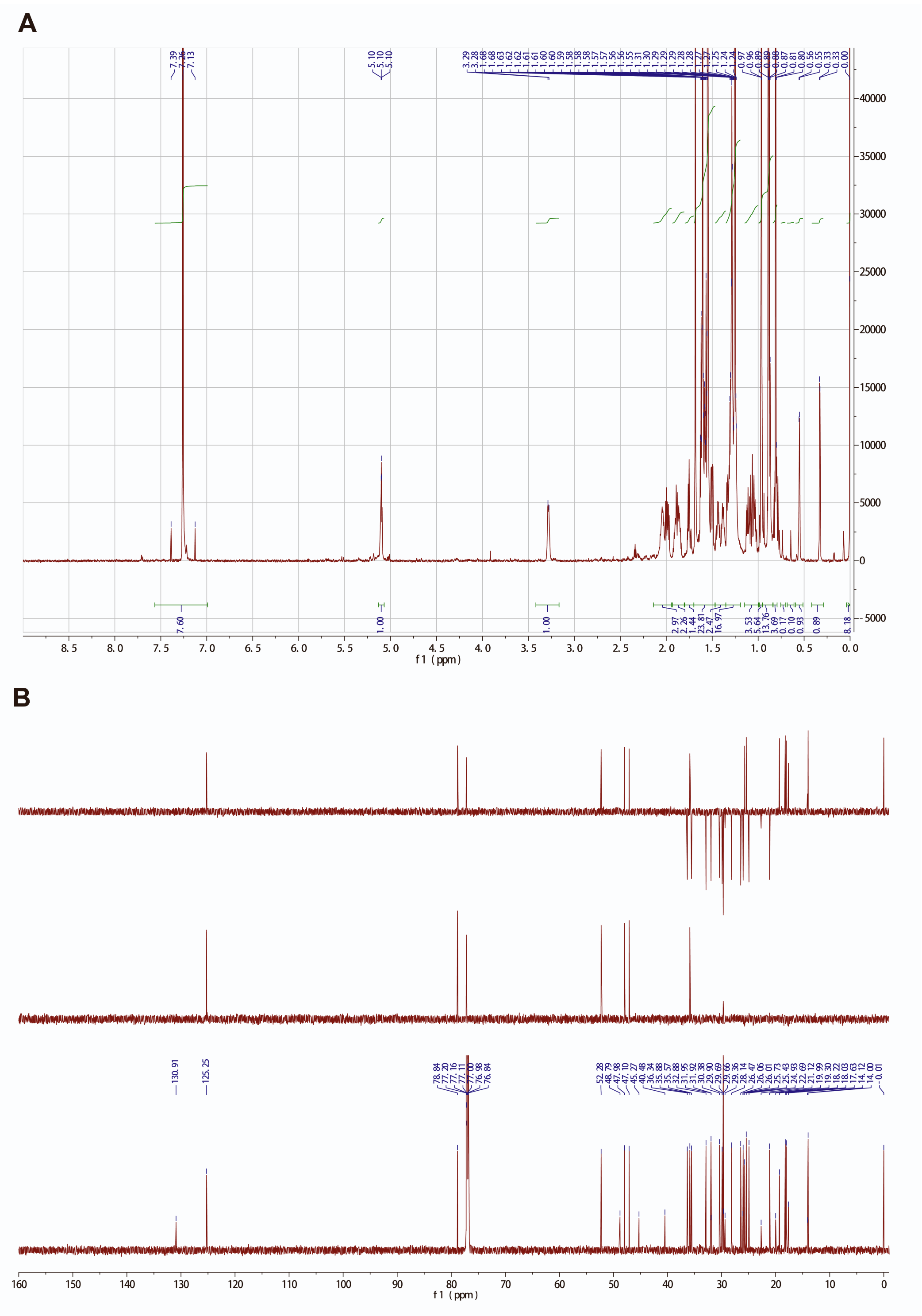
A
f1 (ppm)
B
f1 (ppm)

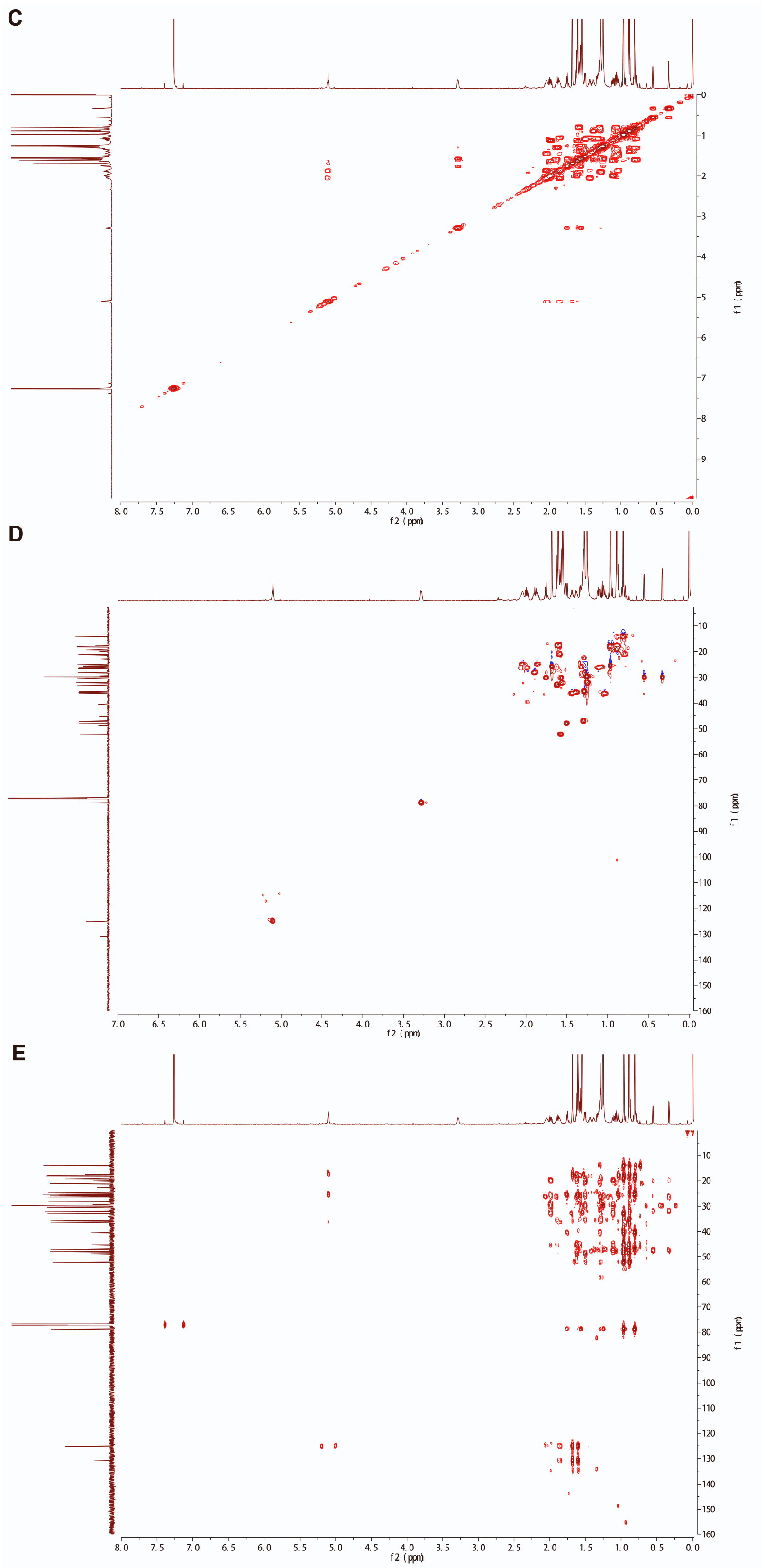

Supplemental Figure 11 NMR spectra of HcOSC1 product. (A) ^{1}H NMR spectrum in $CDCl_3$ at 600 MHz. **(B)** ^{13}C NMR spectrum in $CDCl_3$ at 600 MHz. **(C)** ^{1}H-^{1}H COSY spectrum in $CDCl_3$ at 600 MHz. **(D)** HSQC spectrum in $CDCl_3$ at 600 MHz. (E). HMBC spectrum in $CDCl_3$ at 600 MHz.

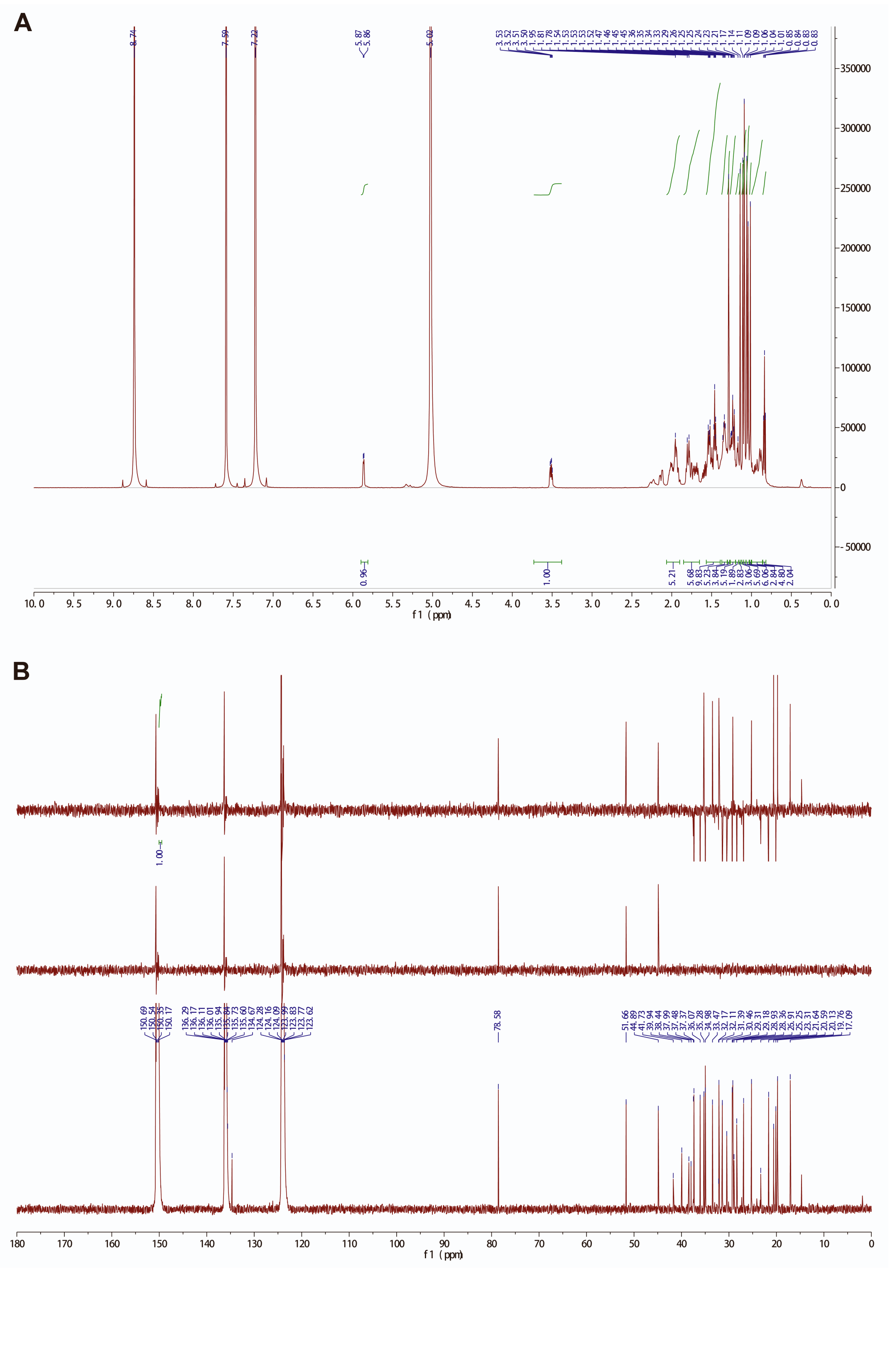
A
B
f1 (ppm)
f1 (ppm)

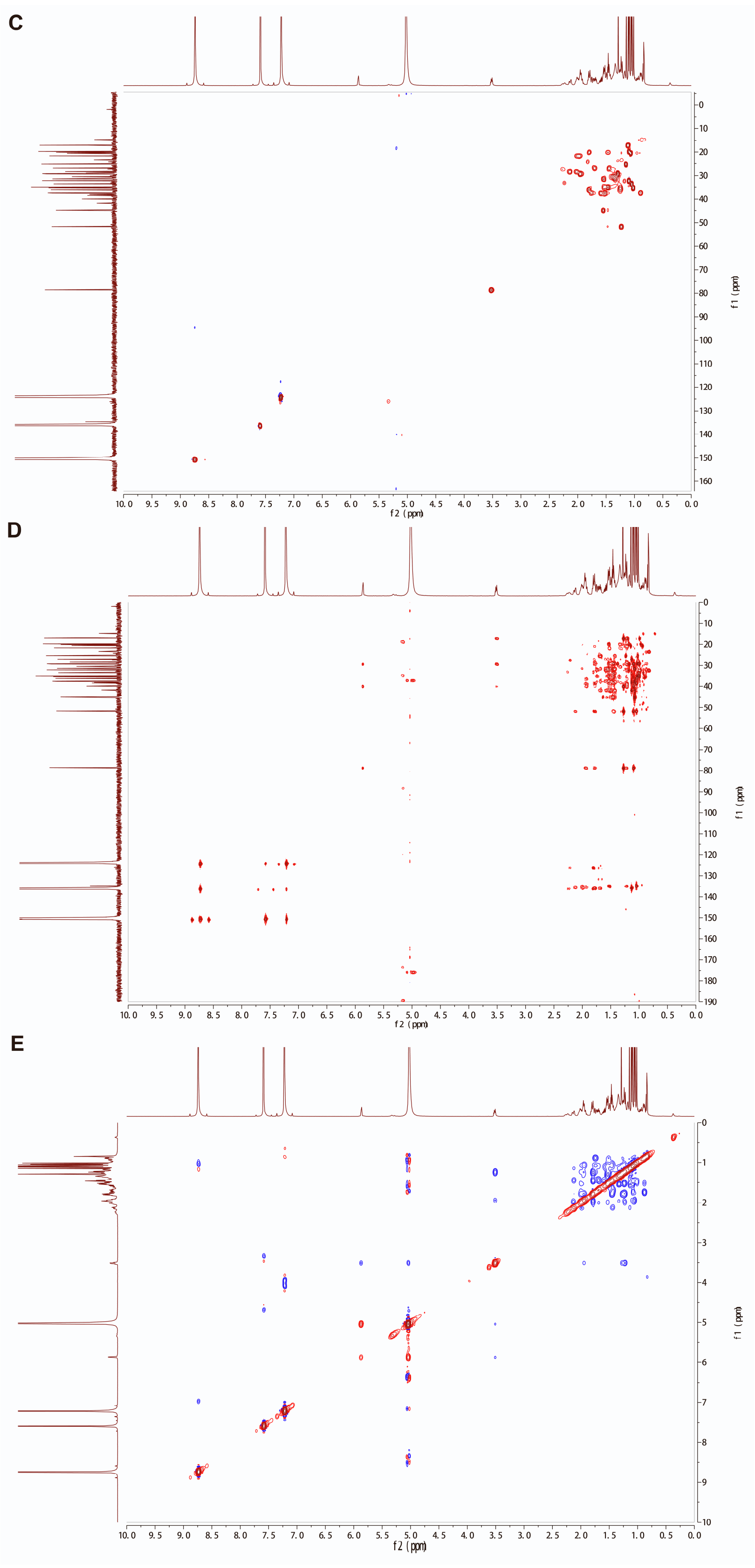

Supplemental Figure 12 NMR spectra of HcOSC5 product. (A) ^{1}H NMR spectrum in C_5D_5N at 600 MHz. **(B)** ^{13}C NMR spectrum in C_5D_5N at 600 MHz. **(C)** ^{1}H-^{1}H HSQC spectrum in C_5D_5N at 600 MHz. **(D)** HMBC spectrum in C_5D_5N at 600 MHz. **(E)** ROESY spectrum in C_5D_5N at 600 MHz.

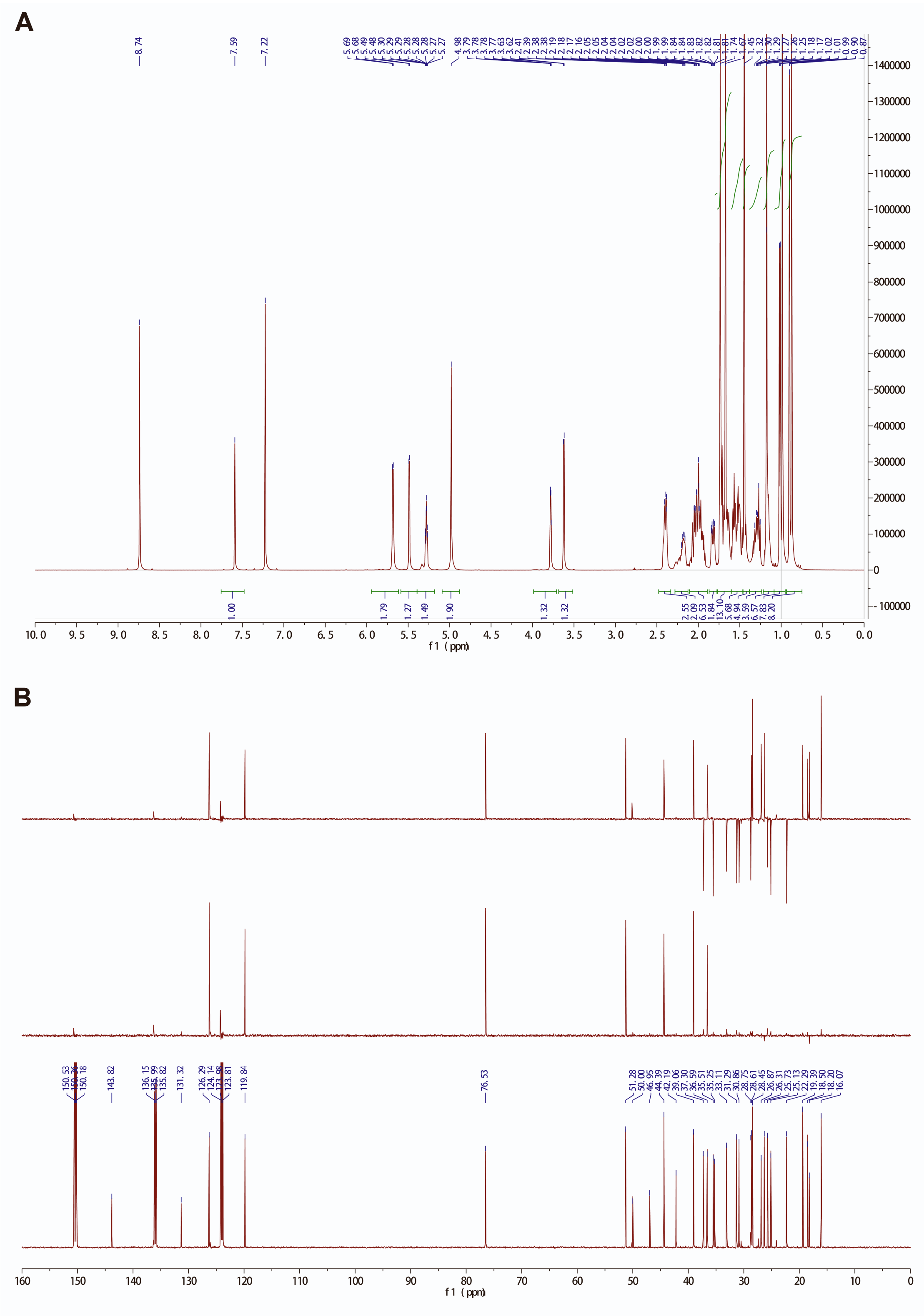
A
8.74
7.59
7.22
4.98
f1 (ppm)
B
143.82
131.32
119.84
76.53
f1 (ppm)

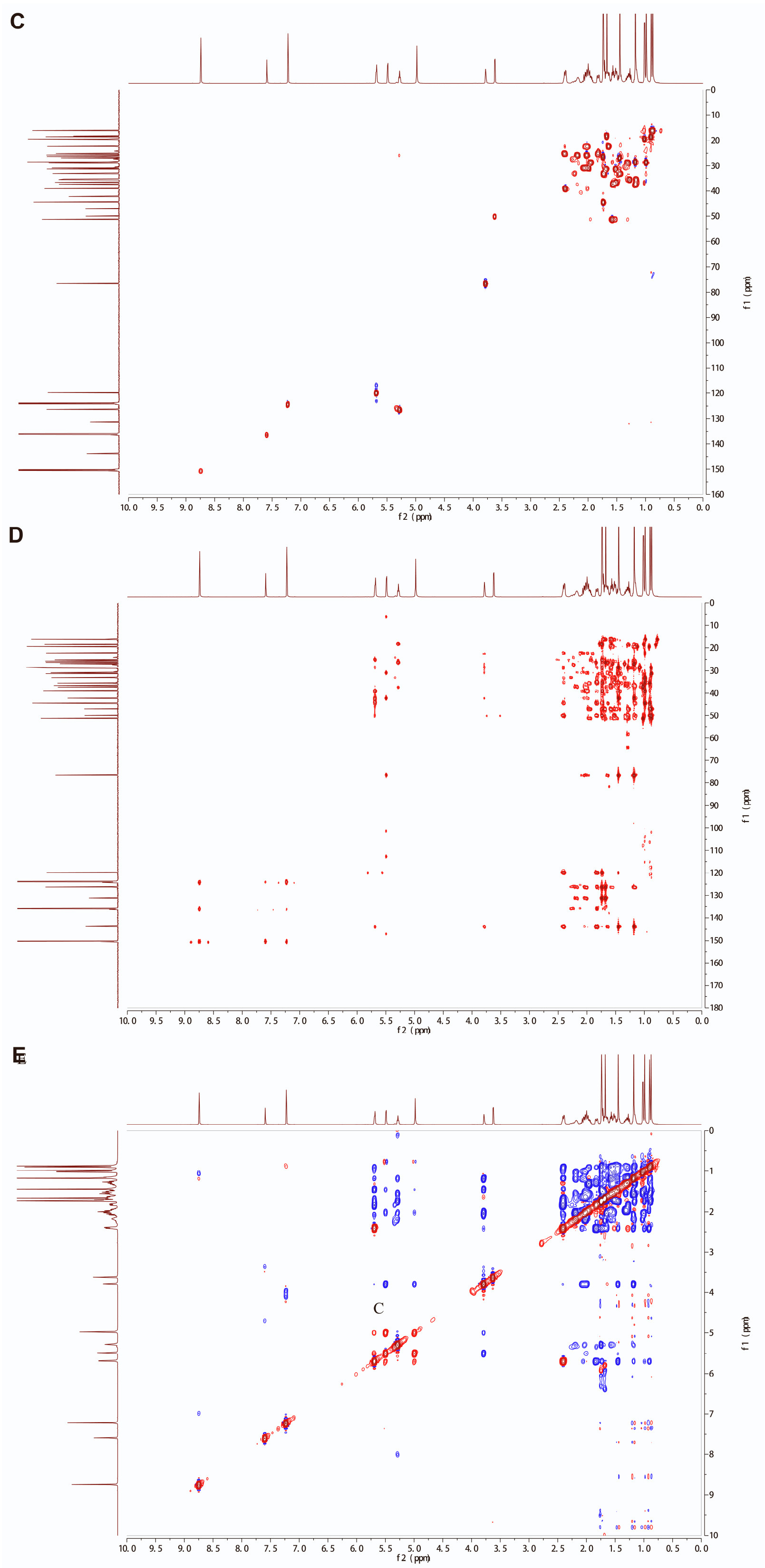

Supplemental Figure 13 NMR spectra of HcOSC6 product. (A). ^{1}H NMR spectrum in C_5D_5N at 600 MHz. **(B)**. ^{13}C NMR spectrum in C_5D_5N at 600 MHz. **(C)**. ^{1}H-^{1}H HSQC spectrum in C_5D_5N at 600 MHz. **(D)**. HMBC spectrum in C_5D_5N at 600 MHz. **(E)** ROESY spectrum in C_5D_5N at 600 MHz.

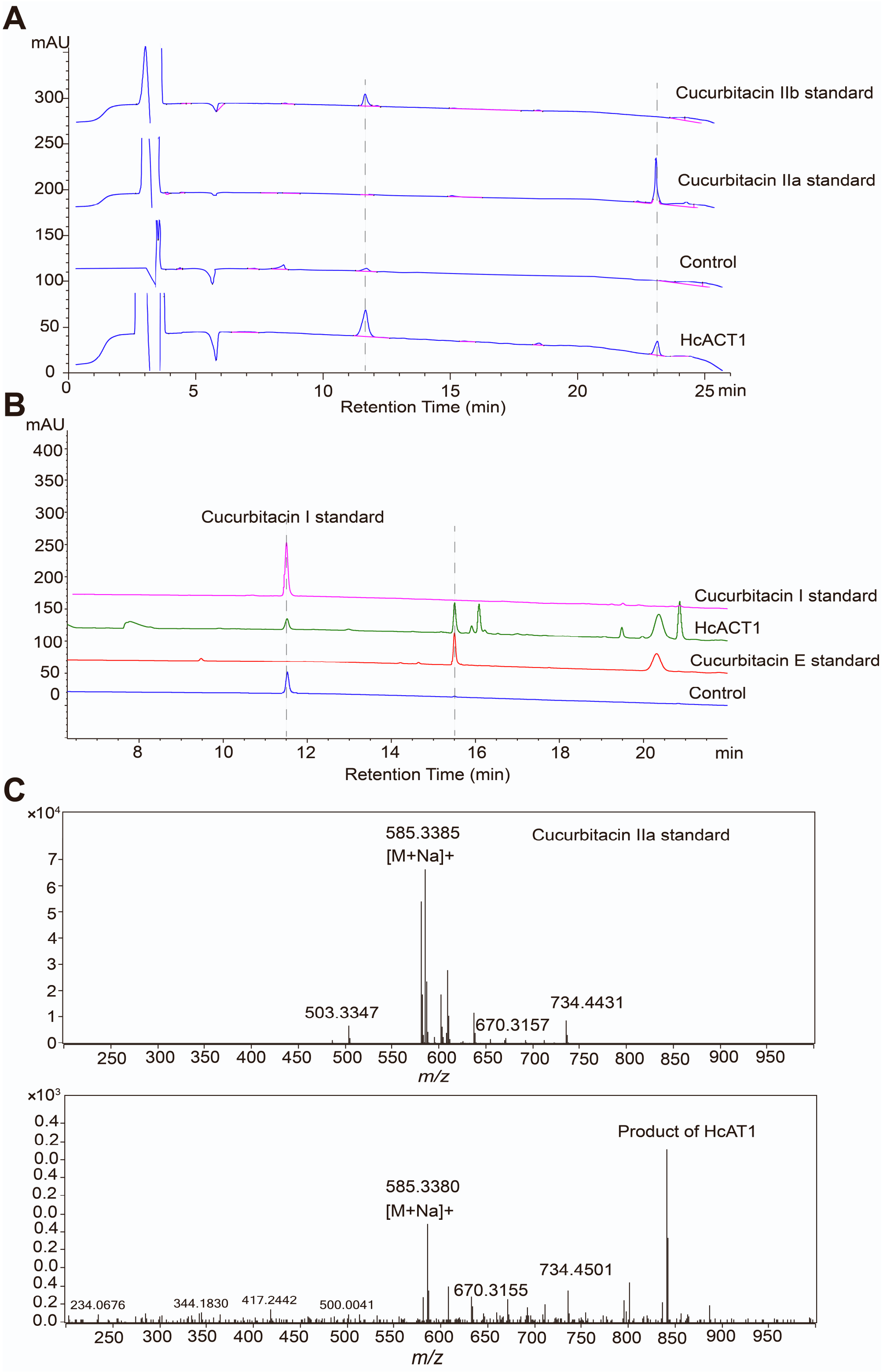

Supplemental Figure 14 HPLC and MS analysis of HcAT1 enzyme catalyzed products. (A) HPLC analysis of HcAT1 enzyme catalyzed CuIIb to produce CuIIa, Contron is a reaction with no enzyme or no substrate added as a negative control. **(B)** HPLC analysis of HcAT1 enzyme catalyzed Cucurbitacin I to produce Cucurbitacin E, Contron is a reaction with no enzyme or no substrate added as a negative control. **(C)** Ion characterization of standard cucurbitacin IIa and products.

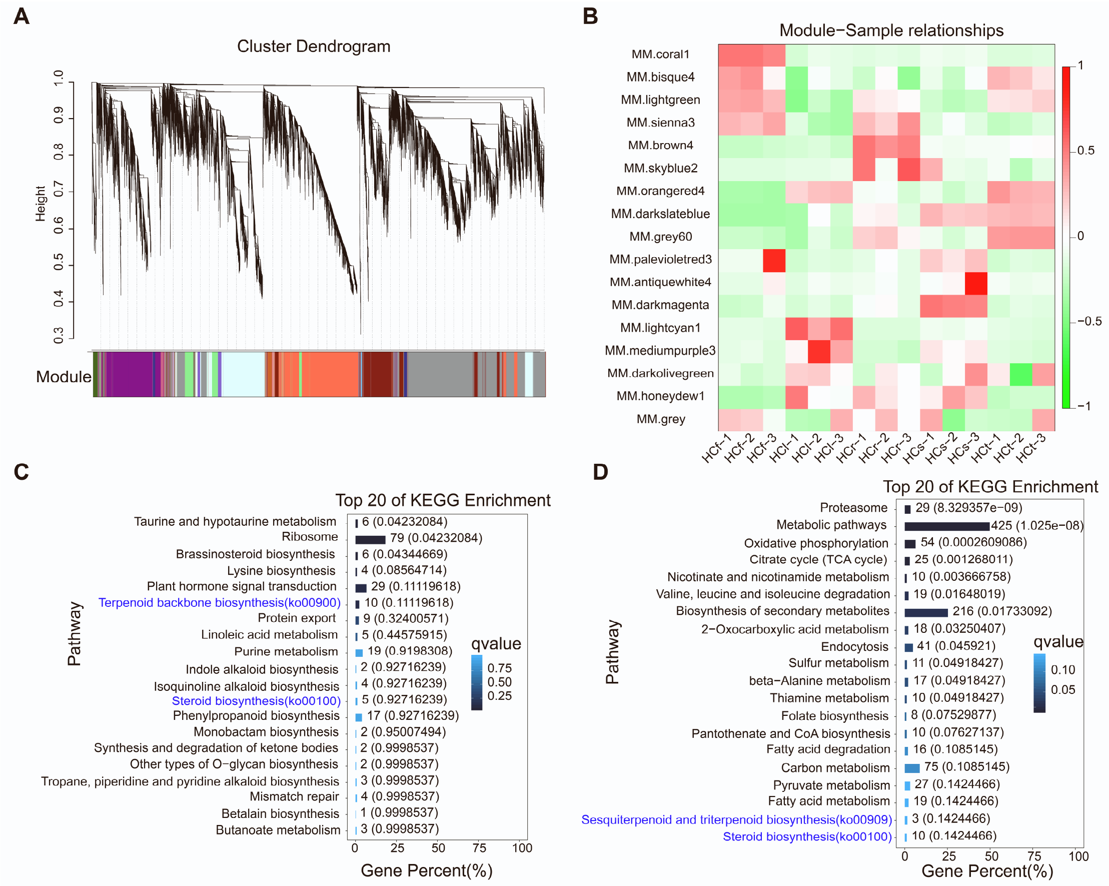

Supplemental Figure 15 WGCNA of differentially expressed genes. (A) Hierarchical cluster tree showing co-expression modules identified by WGCNA. Each leaf in the tree is one gene. The major tree branches constitute 17 modules labelled by different colours. **(B)** Module–sample association. Each row corresponds to a module. The name of modules is indicated on the left. Each column corresponds to a specific sample. The colour of each cell at the row–column intersection indicates the correlation coefficient between the module and sample. A high degree of correlation between a specific module and sample is indicated by red. **(C)** Terpenoid backbone biosynthesis (ko00900) and steroid biosynthesis (ko00100) enriched in module brow4. (D) Sesquiterpenoid and triterpenoid biosynthesis (ko00909) and steroid biosynthesis (ko00100) enriched in module coral1.

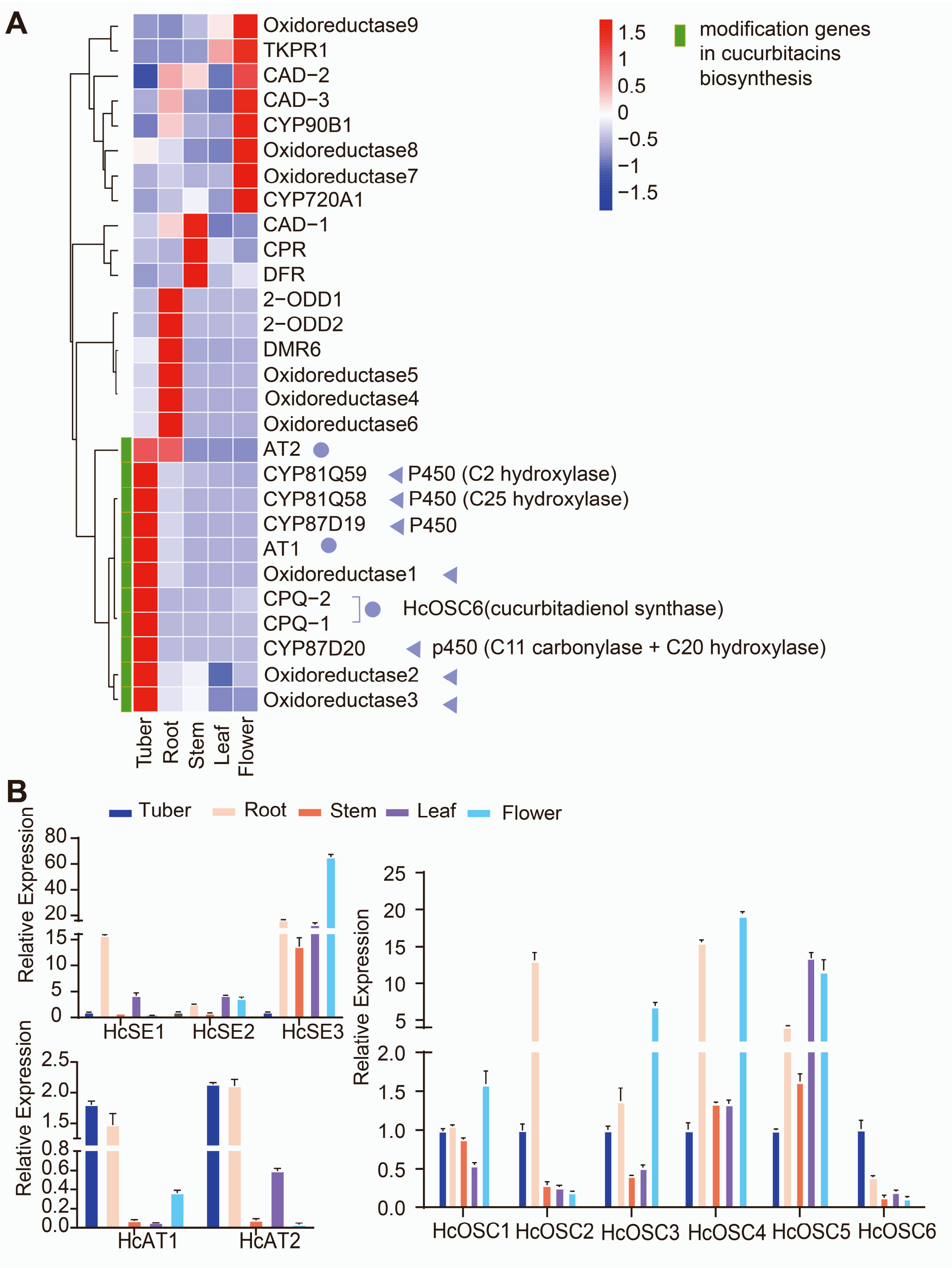

Supplemental Figure 16 Analysis of hierarchical clusterin candidate genes involved in cucurbitacin biosynthesis and expression of *HcSEs*, *HcOSCs*, and *HcATs* in *H.chinensis.* (A) The heatmap highlights the patterns of coexpression of P450 genes with HcOSC6 in the tuber, root, stem, flower and leaf tissues of *H.chinensis*, *CYPs* and o*xidoreductases* genes (blue triangles), are proposed as a candidate gene for modification in cucurbitacins biosynthesis. **(B)** Normalized expression of *HcSEs*, *HcOSCs*, and *HcATs* relative to Hc18s in RNA from steam, tuber, leaf, root, and flower of *H.chinensis* by qRT-PCR. Relative expression levels were calculated using the ΔΔCq method Error bars represent SDs based on triplicate measurements of at least three biological replicates.

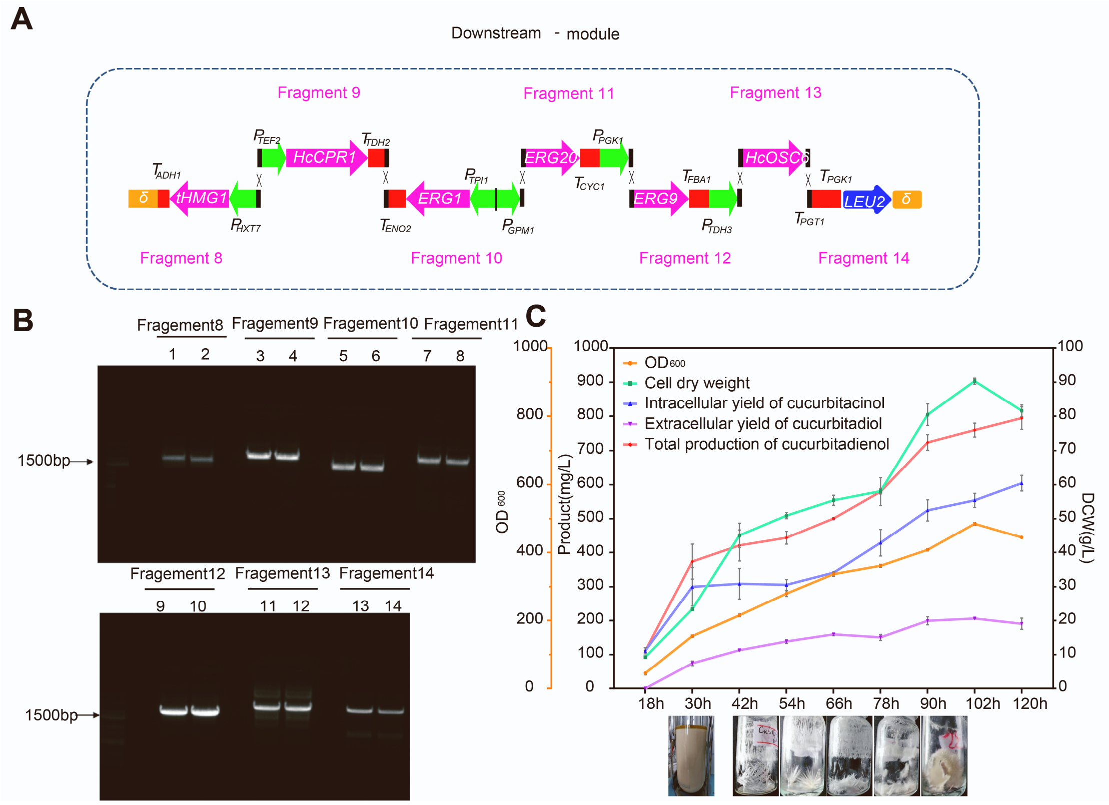

Supplemental Figure 17 Construction of Engineering Yeast Strain Cuol01-1 and strain Cuol01-1 produces Cuol via fed-batch fermentation. (A) Schematic representation the modular construction of strain Cuol01-1. **(B)** Amplification of gene expression cassette fusion fragments in downstream modules. **(C)** Production of cucurbitadienol by strain Cuol01-1 in fed-batch fermentation and purified product of cucurbitadienol after fermentation.

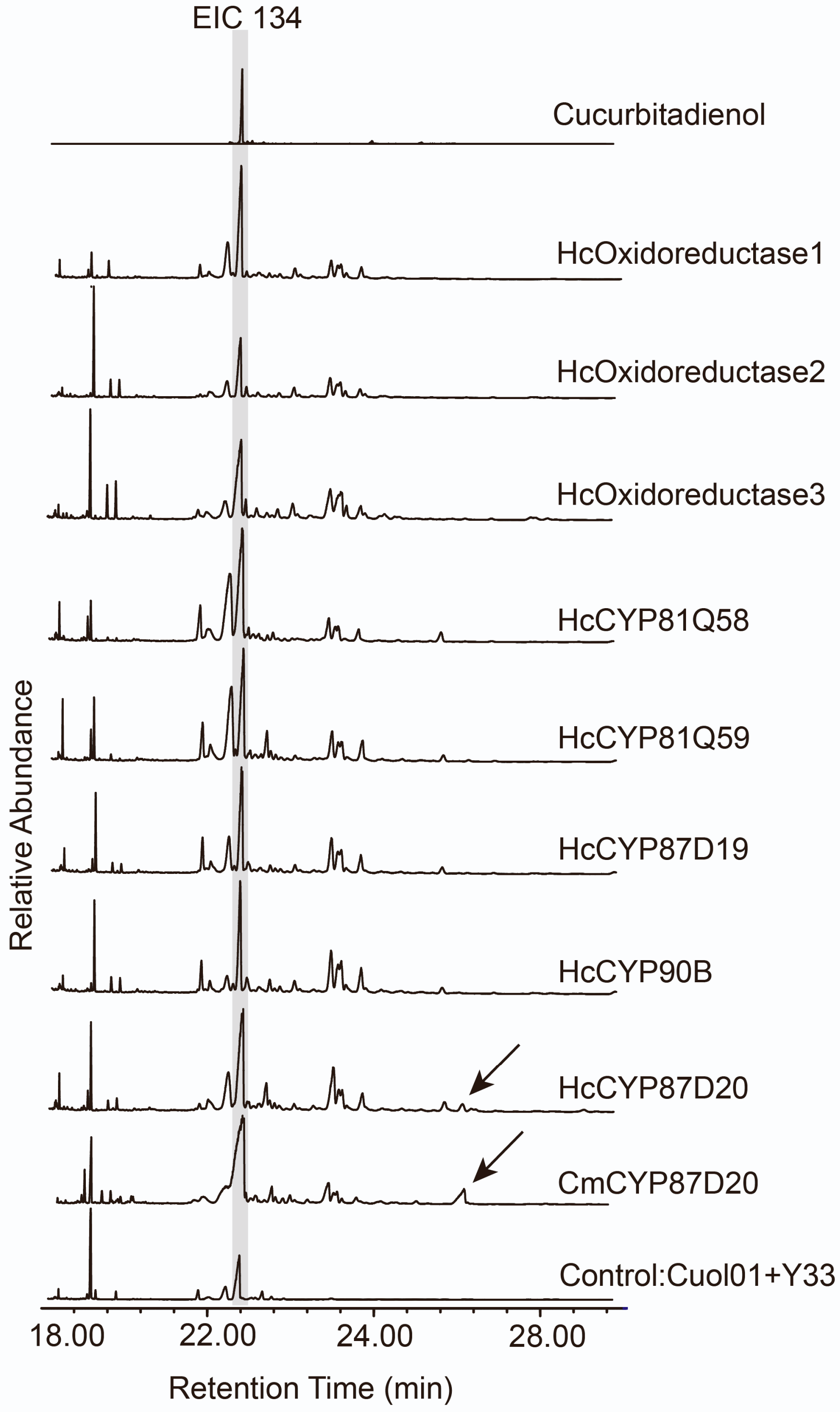

151
152 **Supplemental Figure 18. NMR spectra of 87D20-1 product. GC-MS detection of each CYP**
153 **and each oxidoreductase characterized in yeast Cuol01-1.**

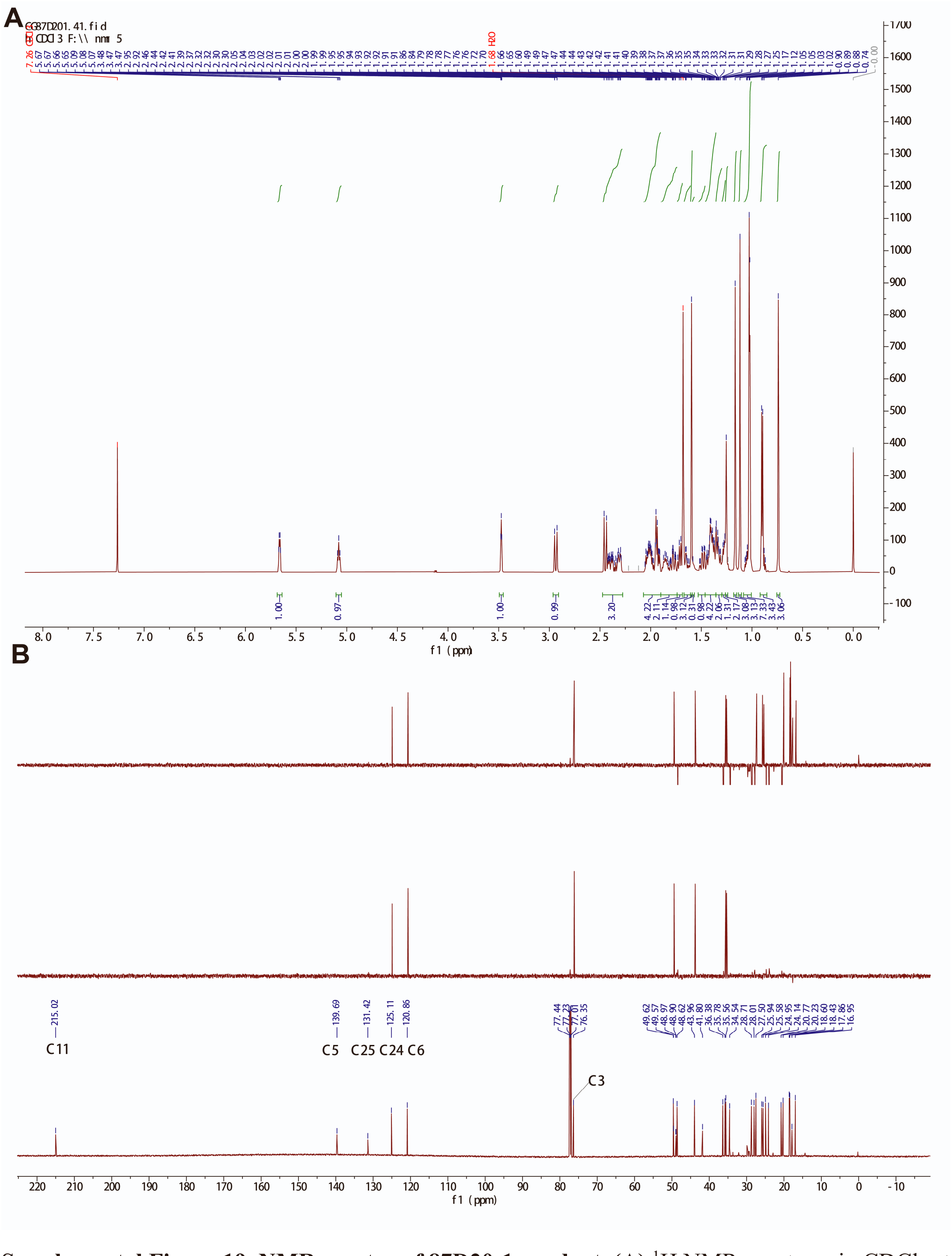

Supplemental Figure 19. NMR spectra of 87D20-1 product. (A) ^{1}H NMR spectrum in $CDCl_3$ at 600 MHz; **(B)** ^{13}C NMR spectrum in $CDCl_3$ at 600 MHz.

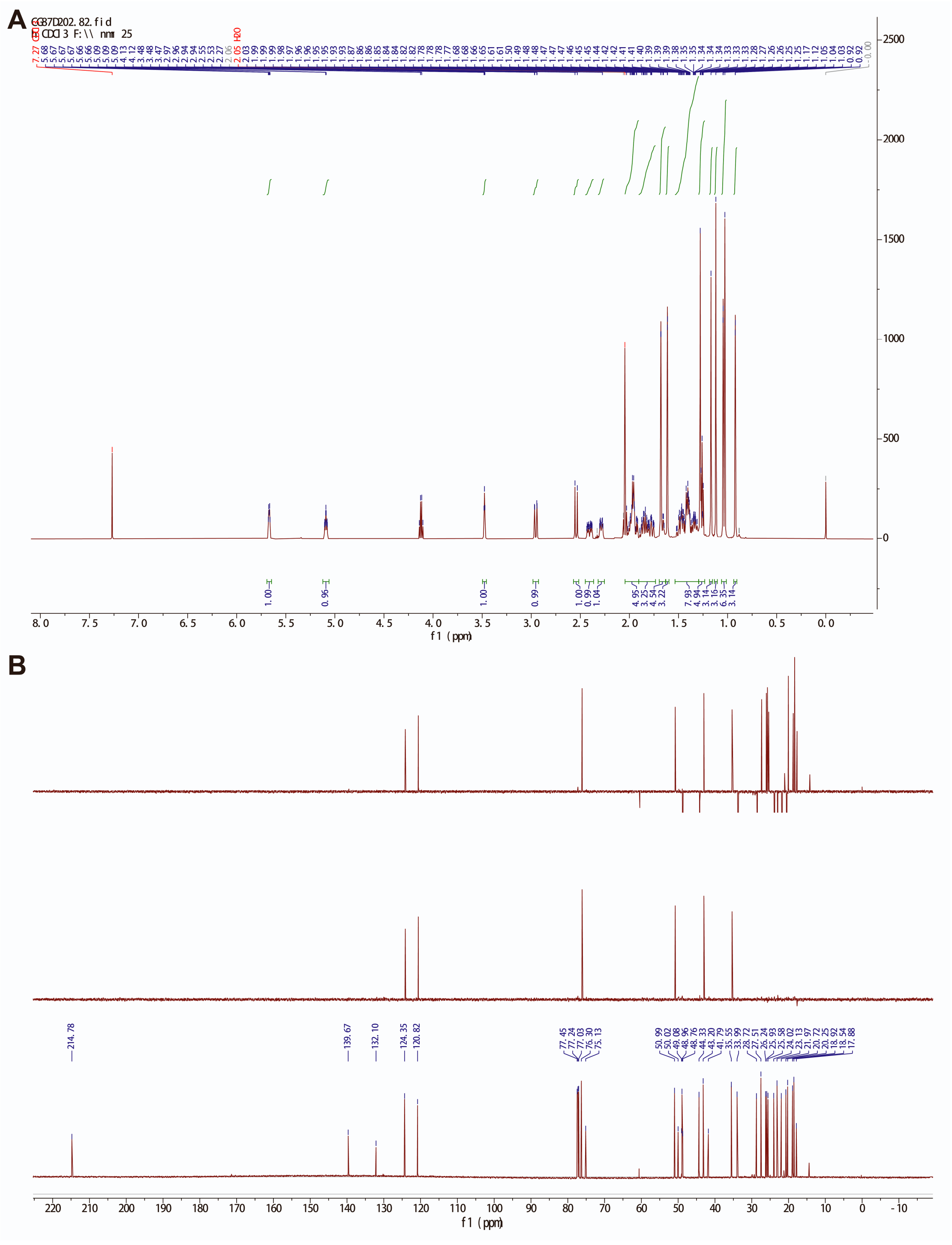

Supplemental Figure 20 NMR spectra of 87D20-2 product. (A) ^{1}H NMR spectrum in $CDCl_3$ at 600 MHz; **(B)** ^{13}C NMR spectrum in $CDCl_3$ at 600 MHz.

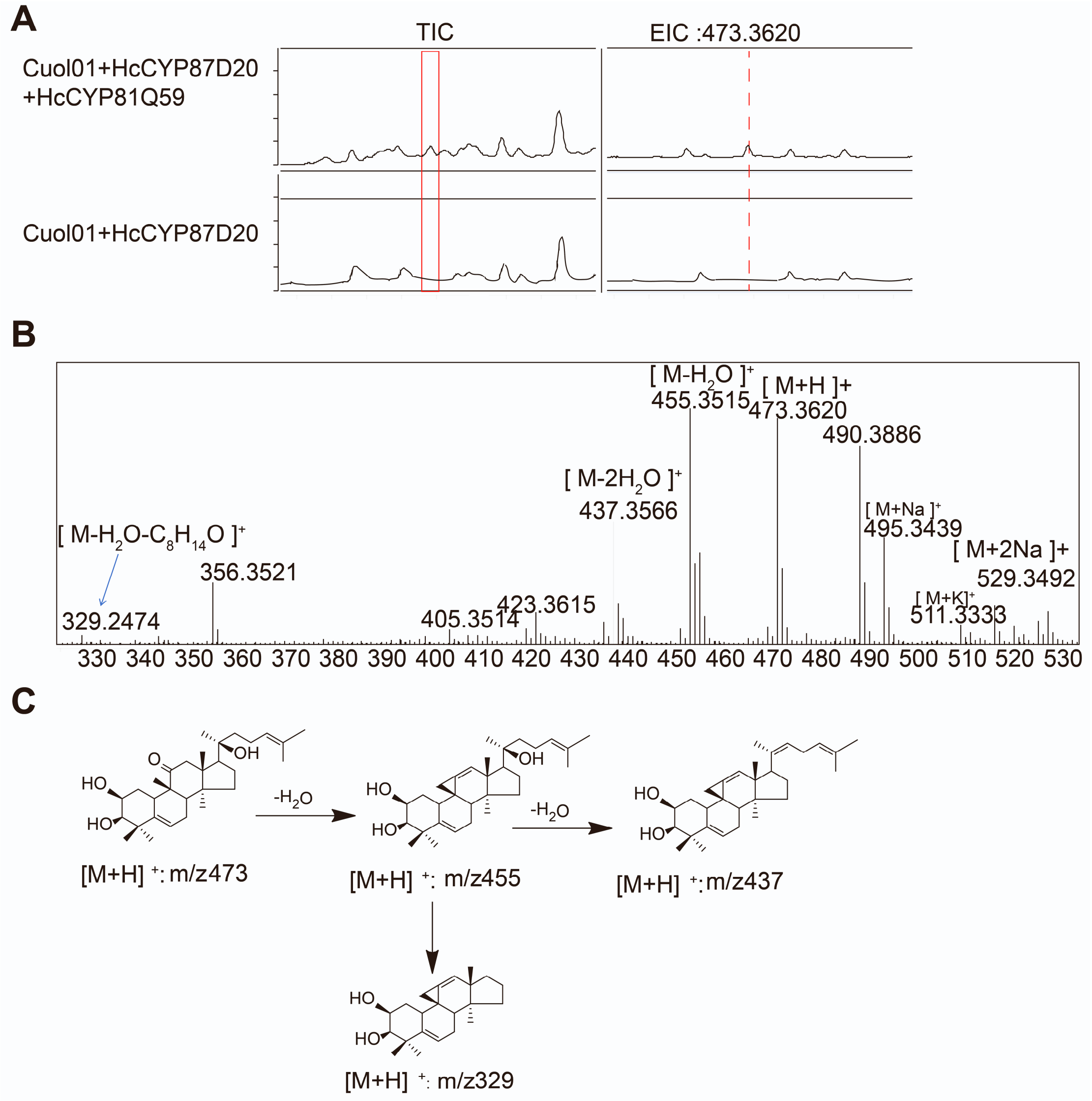

Supplemental Figure 21 UPLC-qTOF-MS analysis of the extracts prepared from yeast accumulating 11-carbonyl-2β,20β-hydroxy-Cuol. (A) One expected product peak (indicated with red line) is generated by the CYP81Q59 enzymes . EIC 473.3620, extracted ion chromatogram of the accurate parent ion at m/z of 473.3620 [M+H], **(B)** Detailed ESI-MS/ on positive ion mode with the CE of 10V, **(C)** Proposed fragmentation pathways of compound 11-carbonyl-2β,20β-hydroxy-Cuol.

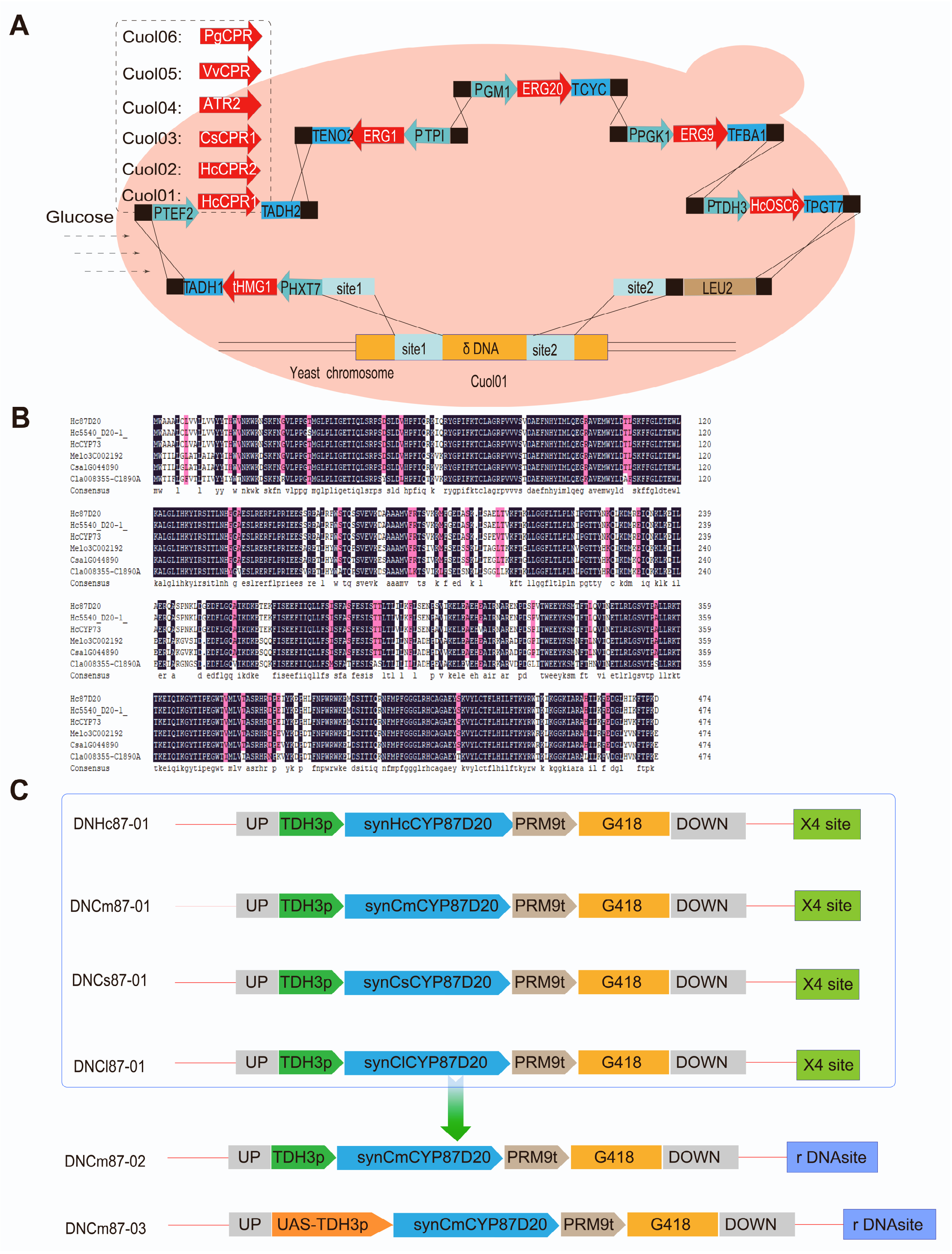

Supplemental Figure 22 Construction of Engineering Yeast Strain Cuol01 to Cuol01-06 and DN01 to DN03. (A) Schematic representation the modular construction of strain Cuol01-06, **(B)** Sequence Comparison of HcCYP87D20 and Cs890, Cm890, Cl890A, **(C)** Schematic representation the modular construction of strain DN01 to DN03.

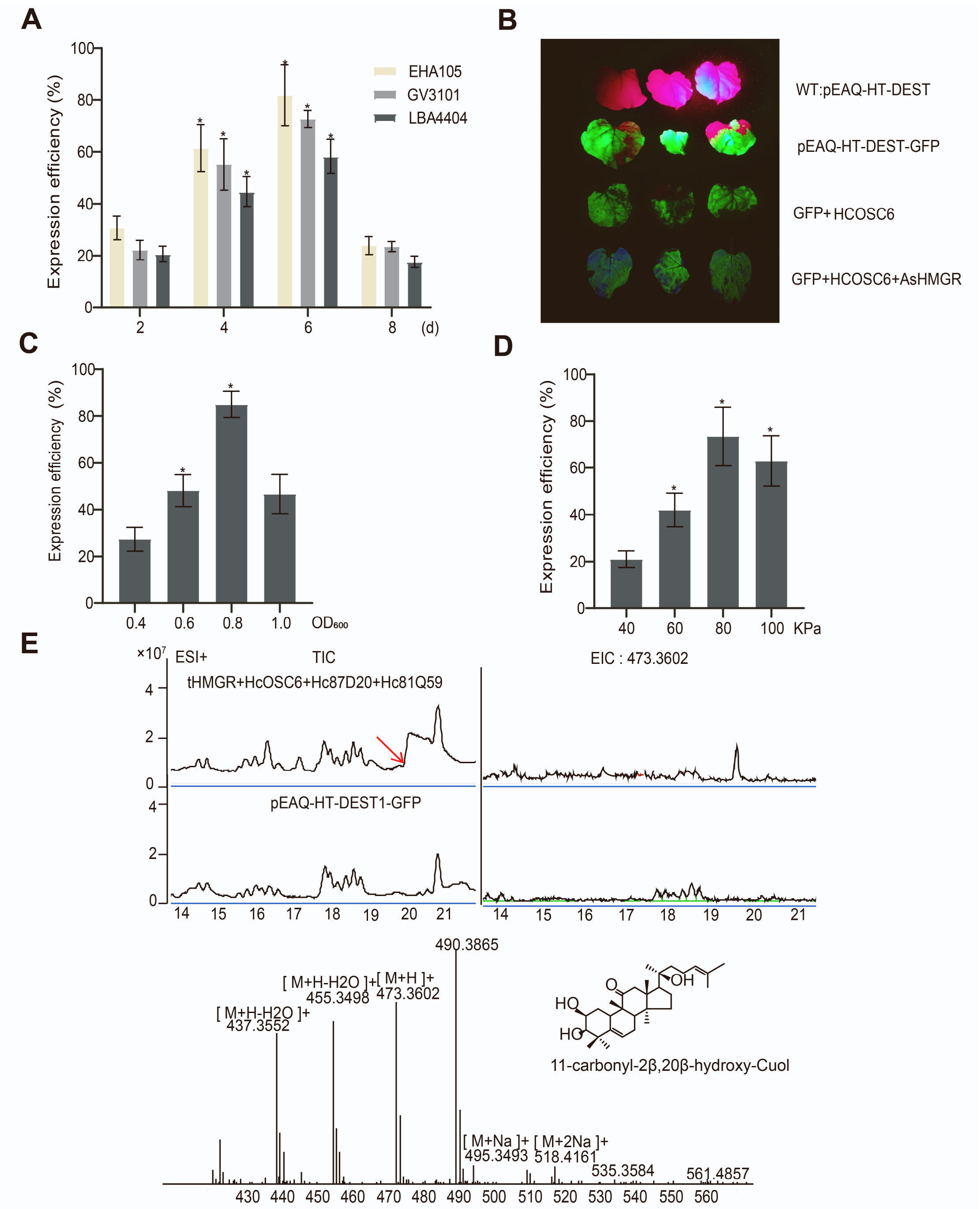

Supplemental Figure 23 Effects of Different Factors of Agrobacterium Infiltration on Transient Expression Efficiency of GFP. (A) Expression efficiency of GFP at different days after different Agrobacterium strains were infiltrated into leaves of *Nicotiana benthamiana*, **(B)** Green fluorescence detection of GFP infiltrated into tobacco expression, **(C)** Efficiency of transient expression of GFP in permeate mediated by Agrobacterium EHA105 at different OD_{600} values, **(D)** Efficiency of transient expression of GFP in permeate mediated by Agrobacterium EHA105 at different Vacuum pressure(MPa), **(E)** LC-qTOF-MS analysis of 11-carbonyl-20β-hydroxy-Cuol products expressed by HcOSC6, Hc87D20 and Hc81Q59 in *Nicotiana benthamiana*

SUPPLEMENTARY TABLES

Supplemental Table 1. Distribution of cucurbitacin IIb, cucurbitacin IIa and oleanolic in *Hemsleya* plans.

Species	tipty	cucurbitacin IIb (mg/g)	cucurbitacin IIa (mg/g)	Free oleanolic (mg/g)	total oleanolic (mg/g)	Source/Location
Hemsleya panlongqi	Wild	0.14±0.011 a	1.43±0.013 a	0.15±0.045 a	7.63±0.025 a	Kun Ming, China (latitude, 102. 1031E; longitude, 25. 0211N)
Hemsleya amabilis	Wild	0.23±0.036 a	2.47±0.372 b	0.31±0.056 a	12.24±0.062 b	
Hemsleya chinensis	Wild	0.46±0.036 b	4.74±0.027 c	0.32±0.094 a	9.51±0.041 a	
Hemsleya carnosiflora	Wild	0.26±0.016 a	2.33±0.347 b	0.20±0.048 a	8.78±0.414 a	
Hemsleya panacis-scandens	Wild	0.15±0.058 a	1.59±0.276 a	0.14±0.031 a	6.24±0.243 c	
Hemsleya macrosperma	Wild	0.23±0.113 a	1.17±0.276 c	0.16±0.013 a	7.89±0.432 a	
Hemsleya mitrata	Wild	ND	ND	1.01±0.302 b	15.53±0.625 b	

Note: ND means not detected, different lowercase letters in the same column indicate significant differences ($P<0.05$).

Supplemental Table 2. Summary of RNA sequencing analysis of five tissue of *H.chinensis*.

Number of totals read use in the assembly	50 ,061 ,733
Q20 percentage	98.01%
GC percentage	40.87%
Number of Unigenes	52,923
N50 of contigs (bp)	1636
Average length of Unigene (bp)	945
Minimum length of Unigene (bp)	201
Maximum length of Unigene (bp)	16,503

Supplemental Table 3. Percentage protein identities of homologs of *H.chinensis*. Sequences and IDs of Homologous genes are from http://www.cucurbitgenomics.org/.

Identified gene	Homologous gene	Percentage protein identities
HcSE1	CsaV32G025870.1	85.96
HcSE1	Cmc03g0085291.1	86.21
HcSE1	ClCG08G016750.1	80.99
HcSE2	CsaV32G025870.1	82.67
HcSE2	Cmc03g0085291.1	82.10
HcSE2	ClCG08G016750.1	76.19
HcOSC6	ClBi (Cla97C06G110880.2)	83.29
HcOSC6	CmBi (MELO3C022374.1)	81.24
HcOSC6	SgCDS (Sgr030492.1)	79.29
HcOSC6	CsBi (CsaV36G007580.1)	81.69
HcOSC6	CpCPQ (Cp4.1LG12g10070.1)	70.96
HcOSC6	McCBS (MC07g0002.1)	82.35
HcAT1	ClACT1(Cla97C06G110890.1)	69.39
HcAT1	CmACT1 (Melo3C022373)	68.49
HcAT1	CsACT1(Cs6G007590.1)	66.59
HcCYP87D20	ClCYP87D20 (Cla008355)	84.21
HcCYP87D20	ClCYP87D20 (MELO3C002192)	84.02
HcCYP87D20	CsCYP87D20 (Csa6G088160)	83.01
HcCPR1	CsCPR	87.72

Supplemental Table 4. ^{13}C & ^{1}H δ assignments for cucurbitadienol (HcOSC6 Product), cycloartenol (HcOSC1 Product), isomultiflorenol(HcOSC5 Product), 87D20-1 Product and 87D20-2 Product.

^{13}C & ^{1}H δ assignments for HcOSC6 Product.

Carbon	$^{\delta}$H (HcOSC6 Product)	$^{\delta}$C (HcOSC6 Product)	$^{\delta}$C (cucurbitadienol)
1	1.64 (m),2.01 (m)	22.29	21.10
2	1.99 (m),2.06 (m)	30.85	28.90
3	3.78 (s, 3H)	76.53	76.60
4	/	42.19	41.40
5	/	143.81	142.20
6	5.49 (d, *J*=5.68 Hz)	119.84	121.40
7	1.82 (m),2.41 (m)	25.13	24.30
8	1.73(m)	44.39	43.60
9	/	35.25	34.40
10	2.41(m)	39.05	37.80
11	1.45 (m),1.72 (m)	33.10	32.30
12	1.29 (m),1.58 (m)	35.51	34.70
13	/	46.94	46.20
14	/	50.00	49.10
15	1.51 (m),1.66 (m)	31.29	30.40
16	1.81 (m),1.95 (m)	28.61	27.90
17	1.58 (m)	51.27	50.40
18	0.87 (s, 3H)	16.06	15.30
19	0.99 (s, 3H)	28.74	28.00
20	1.52 (m)	36.58	35.80
21	1.02(d, *J*=6.30 Hz)	19.39	18.60
22	1.17 (m),1.55 (m)	37.30	36.40
23	1.74 (m),2.02 (m)	25.73	24.80
24	5.28 (t, *J*=7.1 Hz)	126.28	125.20
25	/	131.31	130.90
26	1.73 (s, 3H)	26.87	25.70
27	1.67 (s, 3H)	18.20	17.60
28	1.16 (s, 3H)	28.44	27.20
29	1.45 (s, 3H)	26.31	25.40
30	0.90 (s, 3H)	18.49	17.80

^{13}C & ^{1}H δ assignments for HcOSC1 Product.

Carbon	$^{\delta}$H(HcOSC1 Product)	$^{\delta}$C (HcOSC1 Product)	$^{\delta}$C (cycloartenol)
1	1.24 (m), 1.56 (m)	31.92	32.54
2	1.58 (m), 1.75 (m)	30.38	31.22
3	3.28 (m, 1H)	78.84	78.23
4	/	40.48	41.09
5	1.30 (m, 1H)	47.10	47.64
6	1.58 (m), 1.62 (m)	21.12	21.47
7	1.10 (m), 1.32 (m)	26.01	26.33
8	1.50 (m)	47.98	48.04
9	/	19.99	20.28
10	/	26.06	26.91
11	1.11 (m), 1.99 (m)	26.77	26.93
12	1.25 (m), 1.62 (m)	32.88	33.48
13	/	45.27	45.82
14	/	48.79	49.25
15	1.28 (m), 1.38 (m)	35.57	35.93
16	1.29 (m), 1.89 (m)	28.13	28.43
17	1.58 (m, 1H)	52.28	52.79
18	0.96 (s, 3H)	18.03	18.19
19	0.33 (d, *J*=4.20 Hz,1H), 0.55 (d, *J*=4.00 Hz, 1H)	29.90	29.91
20	1.38 (m, 1H)	35.87	36.23
21	0.89 (s, 3H)	18.22	18.67
22	1.03 (m), 1.47 (m)	36.34	36.82
23	1.86 (m), 2.04 (m)	24.93	25.45
24	5.10 (t, *J*=7.7 Hz)	125.25	125.89
25	/	130.91	130.69
26	1.58 (s, 3H)	17.64	17.67
27	1.68 (s, 3H)	25.73	25.66
28	0.97 (s, 3H)	25.43	26.12
29	0.81 (s, 3H)	14.01	14.66
30	0.88 (d, *J*=6.4 Hz)	19.30	19.62

^{13}C & ^{1} H δ assignments for HcOSC5 Product

Carbon	$^{\delta}$H(HcOSC5 Product)	$^{\delta}$C(HcOSC5 Product)	$^{\delta}$C（isomultiflorenol）
1	1.24(m),1.44(m)	33.47	34.52
2	1.14(m)	26.91	26.46
3	3.51 (dd, *J*=10.8, 5.4Hz)	78.58	78.10
4	/	38.44	38.20
5	1.21(m)	51.66	51.21
6	1.44(m),1.78(m)	21.64	21.18
7	1.27(m),1.93(m)	28.36	27.90
8	/	135.64	135.10
9	/	134.67	134.20
10	/	37.77	37.60
11	1.06(m),1.42(m)	20.13	19.68
12	1.09(m),1.16(m)	31.39	30.95
13	/	41.73	41.40
14	/	39.94	39.50
15	1.93(m),1.99(m)	29.31	28.85
16	1.04(m),1.24(m)	36.07	35.62
17	/	30.46	30.00
18	1.52(m)	44.89	44.43
19	1.01(m),1.44(m)	34.98	34.56
20	/	28.93	28.46
21	0.88(m),1.50(m)	37.37	36.95
22	1.24(m),1.57(m)	37.48	37.02
23	1.11(s, 3H)	29.18	28.71
24	0.84(s, 3H)	17.09	16.60
25	1.01(s, 3H)	19.76	19.28
26	1.14(s, 3H)	25.25	24.81
27	1.04(s, 3H)	20.59	20.13
28	1.14(s, 3H)	32.11	31.65
29	1.06(s, 3H)	35.28	34.77
30	1.09(s, 3H)	32.17	33.03

^{13}C & ^{1}H δ NMR assignments for 87D20-1 Product and ^{13}C of 11-carbonylcucurbitadienol.

Carbon	$^{\delta}$H(87D20-1 Product)	$^{\delta}$C(87D20-1 Product)	$^{\delta}$C（11-carbonyl cucurbitadienol）
1	1.46 (m),1.49 (m)	20.77	20.7
2	1.65 (m),1.78 (m)	28.71	28.6
3	3.47 (m)	76.35	76.3
4	/	41.80	41.7
5	/	139.69	139.6
6	5.66 (d, *J*=5.9 Hz)	120.86	120.8
7	1.90 (m),2.37(m)	24.14	24.1
8	1.94 (d, *J*=7.5 Hz)	43.96	43.9
9	/	48.90	48.8
10	2.29 (d, *J*=12.0 Hz)	35.56	35.5
11	/	215.02	214.9
12	2.43 (d, *J*=14.5Hz),2.92 (d, *J*=14.5Hz)	48.62	48.5
13	/	48.97	48.9
14	/	49.57	49.5
15	1.26 (m),1.35 (m)	34.54	34.5
16	1.85 (m),2.02 (m)	24.95	24.9
17	1.70 (m)	49.62	49.6
18	0.94 (s)	16.95	16.9
19	1.11 (s)	20.23	20.2
20	1.40 (m)	35.78	35.7
21	0.89(d, *J*=6.4 Hz)	18.60	18.5
22	1.05 (m),1.44 (m)	36.38	36.3
23	2.00 (m)	28.01	27.9
24	5.08 (t, *J*=6.5 Hz)	125.11	125
25	/	131.42	131.3
26	1.68 (s)	25.94	25.9
27	1.59 (s)	17.86	17.8
28	1.16 (s)	25.58	25.5
29	1.02 (s)	27.50	27.4
30	1.01 (s)	18.43	18.3

^{13}C & ^{1}H δ assignments for 87D20-2 Product ^{13}C of 11-carbonyl-20β-hydroxycucurbitadienol

Carbon	δH(87D20-2 Product)	δC(87D20-2 Product)	δC（11-carbonyl-20β-hydroxycucurbitadienol）
1	1.40 (m),1.46 (m)	20.72	20.6
2	1.64 (m),1.76 (m)	28.72	28.6
3	3.46 (m)	76.30	76.2
4	/	41.79	41.7
5	/	139.67	139.6
6	5.65 (d, *J*=6.0 Hz)	120.82	120.7
7	1.91 (m),2.37 (m)	24.02	23.9
8	1.94 (d, *J*=8.0 Hz)	43.20	43.1
9	/	49.08	49.0
10	2.26 (d, *J*=12.0 Hz)	35.55	35.5
11	2.52 (d, *J*=13.4 Hz)	214.78	214.7
12	2.93 (d, *J*=14.5Hz)	48.76	48.7
13	/	48.96	48.9
14	/	50.02	49.9
15	1.29 (m),1.37 (m)	33.99	33.9
16	1.90 (m),1.99 (m)	23.13	23.1
17	2.03 (t, *J*=9.3 Hz)	50.99	50.9
18	0.91 (s)	18.92	18.8
19	1.11 (s)	20.25	20.2
20	/	75.13	75.0
21	1.27 (s)	26.24	26.2
22	1.39 (m),1.46 (m)	44.33	44.4
23	1.83 (m)	21.97	21.9
24	5.08 (t, *J*=7.0 Hz)	124.35	124.3
25	/	132.10	132
26	1.67 (s)	25.93	25.8
27	1.60 (s)	17.87	17.8
28	1.16 (s)	25.58	25.5
29	1.02 (s)	27.51	27.4
30	1.03 (s)	18.54	18.5

Supplemental Table 5. 36 genes from RNA-seq analysis of *H.chinensis* highly correlated with *HcOSC6* and *HcAT1* in cytoscape software analysis. The blue font is the bait gene, and the red font is the candidate gene for post-oxidation modifying enzymes.

ID	Symbol or Rename	Description
Key genes involved in the biosynthesis of cucurbitacin and other triterpenes have been characterized		
Unigene0031509	*SQE1*（*HcSE1*）	PREDICTED: squalene monooxygenase [Cucumis sativus]
Unigene0031211	*SQE2*（*HcSE2*）	PREDICTED: squalene monooxygenase-like [Cucumis melo]
Unigene0029607	*SQE3*（*HcSE3*）	PREDICTED: squalene monooxygenase-like [Cucumis sativus]
Unigene0023375	*OSCBPY-1*（*HcOSC2*）	beta-amyrin synthase [Betula platyphylla]
Unigene0037054	*OSCBPY-2*（*HcOSC3*）	PREDICTED: beta-amyrin synthase-like isoform X1 [Cucumis melo]
Unigene0023374	*OSCBPY-3*（*HcOSC4*）	beta-amyrin synthase [Betula platyphylla]
Unigene0021246	*CAS*（*HcOSC1*）	cycloartenol synthase [Siraitia grosvenorii]
Unigene0031773	*IMS1*（*HcOSC5*）	isomultiflorenol synthase [Luffa aegyptiaca]
Unigene0007785	*CPQ-1*（*HcOSC6*）	cucurbitadienol synthase [Siraitia grosvenorii]
Unigene0009883	*CPQ-2*（*HcOSC6*）	cucurbitadienol synthase [Cucumis sativus]
Unigene0022092	*AT1*（*HcAT1*）	PREDICTED: BAHD acyltransferase At5g47980-like [Cucumis melo]
Unigene0027458	*AT2*（*HcAT2*）	PREDICTED: BAHD acyltransferase At5g47980-like [Cucumis sativus]
Unigene0036859	*CPR1*（*HcCPR1*）	PREDICTED: NADPH--cytochrome P450 reductase 2-like [Cucumis sativus]
Proposed as a candidate gene for modification in CuIIa biosynthesis		
Unigene0024047	*CYP81Q58*	C25 hydroxylase for 19-hydroxy-cucurbitadienol [Cucumis sativus]
Unigene0021514	*CYP87D19*	cytochrome P450 [Cucumis sativus]
Unigene0018725	*CYP87D20*	C11 carbonylase and C20 hydroxylase for cucurbitadienol [Cucumis sativus]
Unigene0024806	*CYP81Q59*	C2 hydroxylase for 11-carbonyl-20β-hydroxy-cucurbitadienol [Cucumis melo]
Unigene0022728	*CYP90B1*	PREDICTED: cytochrome P450 90B1 [Cucumis melo]
Unigene0020397	*Oxidoreductase1*	PREDICTED: zerumbone synthase-like, alcohol dehydrogenase [Cucumis sativus]

Unigene0030240	*Oxidoreductase2*	PREDICTED: xanthoxin dehydrogenase [Cucumis melo]
Unigene0027255	*Oxidoreductase3*	PREDICTED: 3beta-hydroxysteroid-dehydrogenase/decarboxylase [Cucumis melo]
Unigene0013479	*CYP720B1*	PREDICTED: abietadienol/abietadienal oxidase [Arachis ipaensis]
Unigene0032303	*CAD-1*	PREDICTED: cinnamoyl-CoA reductase 2-like(CAD) [Cucumis melo]
Unigene0038860	*CAD-2*	PREDICTED: cinnamoyl-CoA reductase 1-like isoform X1(CAD) [Cucumis melo]
Unigene0035592	*CAD-3*	PREDICTED: cinnamoyl-CoA reductase 1-like(CAD) [Juglans regia]
Unigene0049338	*2-ODD1*	PREDICTED: probable 2-oxoglutarate-dependent dioxygenase AOP1 [Cucumis melo]
Unigene0049337	*2-ODD2*	PREDICTED: probable 2-oxoglutarate-dependent dioxygenase AOP1 [Cucumis melo]
Unigene0016819	*Oxidoreductase4*	PREDICTED: short-chain dehydrogenase reductase 3b-like isoform X2 [Lupinus angustifolius]
Unigene0021950	*Oxidoreductase5*	PREDICTED: (-)-isopiperitenol/(-)-carveol dehydrogenase, mitochondrial [Vitis vinifera]
Unigene0015843	*Oxidoreductase6*	PREDICTED: short-chain dehydrogenase reductase 3b-like isoform X2 [Lupinus angustifolius]
Unigene0015842	*Oxidoreductase7*	PREDICTED: short-chain dehydrogenase reductase 3b-like [Citrus sinensis]
Unigene0017247	*Oxidoreductase8*	PREDICTED: secoisolariciresinol dehydrogenase-like [Populus euphratica]
Unigene0016437	*Oxidoreductase9*	PREDICTED: short-chain dehydrogenase/reductase family 42E member 1 [Cucumis melo]
Unigene0007853	*TKPR1*	PREDICTED: tetraketide alpha-pyrone reductase 1 [Ricinus communis]
Unigene0025862	*DFR*	PREDICTED: dihydroflavonol-4-reductase-like [Juglans regia]
Unigene0032407	*DMR6*	PREDICTED: hyoscyamine 6-dioxygenase-like [Cucumis sativus]

Supplemental Table 6. Correlation analysis of oxidoreductase 1 with HcOSC6 and HcAT1.

interaction	correlation	p_value
Unigene0020397（Oxidoreductase1）interacts with Unigene0022092（*HcAT1*）	0.992	5.28E-13
Unigene0020397（Oxidoreductase1）interacts with Unigene0024806（*CYP81Q59*）	0.984	4.83E-11
Unigene0020397（Oxidoreductase1）interacts with Unigene0009883（*CPQ-1*）	0.98	1.99E-10
Unigene0020397（Oxidoreductase1）interacts with Unigene0021514（*CYP87D19*）	0.975	7.00E-10
Unigene0020397（Oxidoreductase1）interacts with Unigene0024047（*CYP81Q58*）	0.974	8.16E-10
Unigene0020397（Oxidoreductase1）interacts with Unigene0018725（*CYP87D20*）	0.972	1.37E-09
Unigene0020397（Oxidoreductase1）interacts with Unigene0007785（*CPQ-2*）	0.963	9.44E-09
Unigene0020397（Oxidoreductase1）interacts with Unigene0027255	0.847	6.80E-05
Unigene0020397（Oxidoreductase1）interacts with Unigene0027458	0.821	1.75E-04
Unigene0020397（Oxidoreductase1）interacts with Unigene0021246	0.379	1.63E-01
Unigene0020397（Oxidoreductase1）interacts with Unigene0036238	0.154	5.85E-01
Unigene0020397（Oxidoreductase1）interacts with Unigene0037054	0.011	9.70E-01
Unigene0020397（Oxidoreductase1）interacts with Unigene0005416	-0.02	9.44E-01
Unigene0020397（Oxidoreductase1）interacts with Unigene0034192	-0.037	8.96E-01
Unigene0020397（Oxidoreductase1）interacts with Unigene0032407	-0.046	8.72E-01
Unigene0020397（Oxidoreductase1）interacts with Unigene0023375	-0.067	8.11E-01
Unigene0020397（Oxidoreductase1）interacts with Unigene0031509	-0.073	7.97E-01
Unigene0020397（Oxidoreductase1）interacts with Unigene0049338	-0.084	7.67E-01
Unigene0020397（Oxidoreductase1）interacts with Unigene0049337	-0.095	7.35E-01
Unigene0020397（Oxidoreductase1）interacts with Unigene0036859	-0.166	5.55E-01
Unigene0020397（Oxidoreductase1）interacts with Unigene0022098	-0.193	4.91E-01
Unigene0020397（Oxidoreductase1）interacts with Unigene0032872	-0.223	4.24E-01
Unigene0020397（Oxidoreductase1）interacts with Unigene0023374	-0.224	4.23E-01
Unigene0020397（Oxidoreductase1）interacts with Unigene0035287	-0.238	3.92E-01
Unigene0020397（Oxidoreductase1）interacts with Unigene0035286	-0.239	3.91E-01
Unigene0020397（Oxidoreductase1）interacts with Unigene0013479	-0.248	3.74E-01
Unigene0020397（Oxidoreductase1）interacts with Unigene0031773	-0.253	3.63E-01
Unigene0020397（Oxidoreductase1）interacts with Unigene0016437	-0.286	3.02E-01
Unigene0020397（Oxidoreductase1）interacts with Unigene0022728	-0.318	2.48E-01

Unigene0020397（Oxidoreductase1）interacts with Unigene0029607	-0.384	1.57E-01
Unigene0020397（Oxidoreductase1）interacts with Unigene0031211	-0.553	3.24E-02

Supplemental Table 7. Fermentation results of engineered strain in shake flasks.

Strains	Cuol (mg/L)	11-carbonyl-Cuol(mg/L)	11-carbonyl-20β-hydroxy-Cuol(mg/L)
Cuol01-1	133.21 ±2.01	-	-
Cuol01-1-PHc87	123.56 ±3.27	4.27 ±0.68	1.52 ±0.08
Cuol01-1-PCm87	120.88 ±1.46	4.83 ±0.89	1.65 ±0.09
DNHc87-01	102.37 ±3.93	10.43 ±0.34	5.58 ±0.10
DNCm87-01	99.42 ±2.76	16.55 ±0.89	8.47 ±0.21
DNCs87-01	116.71 ±3.56	7.21 ±0.58	3.62 ±0.18
DNCl87-01	110.35 ±1.39	6.81 ±0.21	3.77 ±0.07
DNCm87-02	74.19 ±3.83	21.57 ±0.93	28.45 ±0.14
DNCm87-03	64.21 ±2.38	15.85 ±0.62	46.41 ±0.15

All given data in this table representing mean values from three repeats with corresponding standard deviations

Supplemental Table 8. Yeast strains used in this study.

Strains	Genotype or characteristic	Resource
(GIL77)	MA Ta/MA Talpha, his3Δ1/his3Δ1, leu2Δ0/leu2Δ0, lys2Δ0/+met15Δ0/+ura3Δ0/ura3Δ0 ΔERG7(Lanosterol synthase-Deficient yeast)	ATCC
BY4742	MATα, his3Δ1, leu2Δ0, lys2Δ0, ura3Δ0	ATCC
CUOL01-1	Downstream module (*HXT7p-tHMG1-ADH1t, TEF2p-synHcCPR1-TDH2t, TPIIp-ERG1-ENO2t, GPM1p-ERG20-CYC1t, PGK1p-ERG9-FBA1t, TDH3p-synHc0SC6-PGT1t, and LEU2* marker gene) integrated into δ DNA site of BY4742	This study
Cuol01-1-PHc87	*Y33-synHcCYP87D20*	This study
Cuol01-1-PCm87	*Y33-synCmCYP87D20*	This study
Cuol01-1-PHc87+HcQ59	Y33-*synHcCYP87D20+synHcCYP81Q59*	This study
Cuol01-1-PCm87+CmQ59	Y33-synCm*CYP87D20+synCmCYP81Q59*	This study
DNHc87-01	*CUOL01-1-RS (X-4 :: TDH3p-HcCYP87D20-PRM9t)*	This study
DNCm87-01	*CUOL01-1-RS (X-4 :: TDH3p-CmCYP87D20-PRM9t)*	This study
DNCs87-01	*CUOL01-1-RS (X-4 :: TDH3p-CsCYP87D20-PRM9t)*	This study
DNCl87-01	*CUOL01-1-RS (X-4 :: TDH3p-ClCYP87D20-PRM9t)*	This study
DNCm87-02	*CUOL01-1(rDNA :: TDH3p--CmCYP87D20-PRM9t)*	This study
DNCm87-03	*CUOL01-1(rDNA :: UAS-TDH3p--CmCYP87D20-PRM9t)*	This study

Supplemental Table 9. Plasmid used in this study.

plasmid	describe	Resource
YCplac33	Centromeric vector, URA3, Amp	Liu et al
YCplac33-PE	Centromeric vector*, URA3,* Amp, *pPGK1 and tCYC1*	GENEWIZ
pUC57-Kan	Cloning vector ,Cloning site: EcoRV,KanR	GENEWIZ
pEAQ-HT-DEST1	Plant binary transient expression vector,KanR	Reed J et al
pAN580-*GFP*	Construction of subcellular targeting vectors,KanR	Towin Biotechnology
Y33-*synHcCYP87D20*	YCplac33-PE vector,pPGK1-*synHcCYP87D20*-tCYC1	This study
Y33-*synHcCYP81Q59*	YCplac33-PE vector, pPGK1-*synHcCYP81Q59*-tCYC1	This study
Y33-*synCs87D20*	YCplac33-PE vector, pPGK1-*synCs87D20*-tCYC1	This study
Y33-*synMe87D20*	YCplac33-PE vector,PGK1-*synMe87D20*-tCYC1	This study
Y33-*synCl87D20*	YCplac33-PE vector, pPGK1-*synCl87D20*-tCYC1	This study
Y33-*HcOxidoreductase1*	YCplac33-PE vector, pPGK1-*HcOxidoreductase1*-tCYC1	This study
Y33-*HcOxidoreductase2*	YCplac33-PE vector, pPGK1-*HcOxidoreductase2*-tCYC1	This study
Y33-*HcOxidoreductase3*	YCplac33-PE vector, pPGK1-*HcOxidoreductase3*-tCYC1	This study
Y33-*HcCYP87D19*	YCplac33-PE vector, pPGK1-*HcCYP87D19*-tCYC1	This study
Y33-*HcCYP81Q58*	YCplac33-PE vector, pPGK1-*HcCYP81Q58*-tCYC1	This study
Y33-*HcCYP90B*	YCplac33-PE vector, pPGK1- *HcCYP90B*-tCYC1	This study
Y33-*synHcCYP87D20+synHcCYP81Q59*	YCplac33-PE vector, pTDH3-*synHcCYP87D20*-tPFK1+pADH1-*synHcCYP81Q59*-tGPD1	This study
Y33-syn*CmCYP87D20+synCmCYP81Q59*	YCplac33-PE vector, pTDH3-*HcOxidoreductase3*-tPFK1+pADH1-*synMeCYP81Q59*-tGPD1	This study
pEAQ-HT-DEST1-*GFP*	pEAQ-HT-DEST1vector,Gateway entry Clone *GFP*	This study
pEAQ-HT-DEST1-*HcOSC6*	pEAQ-HT-DEST1vector,Gateway entry Clone *HcOSC6*	This study
pEAQ-HT-DEST1-*HcCYP87D20*	pEAQ-HT-DEST1vector,Gateway entry Clone *HcCYP87D20*	This study
pEAQ-HT-DEST1-AstHMGR	pEAQ-HT-DEST1vector,Gateway entry Clone *AstHMGR*	This study
pEAQ-HT-DEST1-HcSQS	pEAQ-HT-DEST1vector,Gateway entry Clone *HcSQS*	This study

pEAQ-HT-DEST1-HcSE1	pEAQ-HT-DEST1vector,Gateway entry Clone *HcSE1*	This study
pEAQ-HT-DEST1-HcSE2	pEAQ-HT-DEST1vector,Gateway entry Clone *HcSE2*	This study
pYES2-HcOSC1	pYES2-URA vector,BamH-HcOSC1	This study
pYES2-HcOSC2	pYES2-URA vector,BamH-HcOSC2	This study
pYES2-HcOSC3	pYES2-URA vector,BamH-HcOSC3	This study
pYES2-HcOSC4	pYES2-URA vector,BamH-HcOSC4	This study
pYES2-HcOSC5	pYES2-URA vector,BamH-HcOSC5	This study
pYES2-HcOSC6	pYES2-URA vector,BamH-HcOSC6	This study

212

Supplemental Table 10. The standard curve of different products.

products	Standard curve	R^2
Squalene	$Y = 1288.4X + 221.563$	0.9991
Ergosterol	$Y = 856.92X + 551.382$	0.9995
Cuol	$Y = 953.07X + 111.85$	0.9999
11-carbonyl-Cuol	$Y= 788.36X + 69.639$	0.9998
11-carbonyl-20β-hydroxy-Cuol	$Y = 475.02X+ 38.665$	0.9999
CuII a	$Y = 2362.6X - 5.8012$	0.9997
CuII b	$Y = 6807.2X + 24.751$	0.9999
Oleanolic	$Y = 365.86X - 4.027$	0.9999

Supplemental Table 11. Primers used in this study.

Gene ID	primers with homology arms(Vector construction: pMAL-c2x)
HcSE1	5'F: aggatttcagaattcggatccATGGCCTGGGGATGGATTT<br>3'R: caggtcgactctagaggatccAGCTATGGGTTTAATACCGGGTGG
HcSE2	5'F: aggatttcagaattcggatccATGCTGGGTCACTGCTGCTTAGG<br>3'R: caggtcgactctagaggatccTCTTCCTCTTTCAGGGGGTGG
HcSE3	5'F: aggatttcagaattcggatccATGGACTTGATTGACTCTGCAAC<br>3'R: caggtcgactctagaggatccCATAATAGGAGGGGCTCTGTAAT
HcCPR1	5'F: ccatggctgatatcggatccATGGAATCGGAATCGAGTTCCA<br>3'R: tgtcgacggagctcgaattcTCACCAGGCTCACGTAGATACC
HcAT1	5'F: ccatggctgatatcggatccATGGAGACTAGATTGAAAGTG<br>3'R: tgtcgacggagctcgaattcCTAAACTTGCTAACATTAGGATTTATGG
HcAT2	5'F: ccatggctgatatcggatccATGAAGATGGAGCATAAAGTTGAAATC<br>3'R: tgtcgacggagctcgaattcCTAGACGACACGGCATGATTGACG
	primers with homology arms(Vector construction: pYES2)
HcOSC1	5'F: ttggtaccgagctcggatcc ATGGCATCTAAAGATTGGAAC<br>3'R: cactggcggccgttactagtTTATACAGCGCATTCTGGCAGGAC
HcOSC2	5'F: ttggtaccgagctcggatccATGTGGAGTAGGATAGATTGC<br>3'R: cactggcggccgttactagtTCAAAAAGAAGCGGAGGTAATTTCTC
HcOSC3	5'F: ttggtaccgagctcggatccATGTGGCGGAATCGAGATTGC<br>3'R: cactggcggccgttactagtTCAAGTAGAAATGCTTGGAGGC
HcOSC4	5'F: ttggtaccgagctcggatccATGTGGAGAGCTGAAGTTGGGAG<br>3'R: cactggcggccgttactagtCTAAATAACGCCTGACACGACAATGC
HcOSC5	5'F: ttggtaccgagctcggatccATGTGGAGGCGGTAGAATAGCAG<br>3'R: cactggcggccgttactagtTCATGGTTGTGAACTAAAGG

HcOSC6	5'F:	ttggtaccgagctcggatccATGTGGAATTAGCGGAGGAG
	3'R:	cactggcggccgttactagtTCAGAAGCATGCGGGGGATG
	primers with homology arms(Vector construction: PEAQ-HT)	
HcSQS	5'F:	ctgcccaaattcgcgaccggtATGGGAAGTTTGGGAGCGATTCTTAG
	3'R:	tgaaaccagagttaaaggcctcgagTCAAAAGATATAAAGATTGAGGACAGTG
HcSE1	5'F:	ctgcccaaattcgcgaccggtATGGCCTGGGGATGGATTT
	3'R:	tgaaaccagagttaaaggcctcgagAGCTATGGGTTTAATACCGGGTGG
HcSE2	5'F:	ctgcccaaattcgcgaccggtATGTGGAAGTTAAAGATAGGAGGAGAG
	3'R:	tgaaaccagagttaaaggcctcgagTCTTCCTCTTTCAGGGGGTGG
HcOSC6	5'F:	ctgcccaaattcgcgaccggtATGTGGAAGTTAAAGATAGGAGGAGAG
	3'R:	tgaaaccagagttaaaggcctcgagTCAGAATAAAGCGGCCGGATG
AstHMGR	5'F:	ctgcccaaattcgcgaccggtCACCATGGCGCCCGAGAAAATGCCCGAG
	3'R:	tgaaaccagagttaaaggcctcgagTCAGCAGGCGATCTTGGACATG
GFP	5'F:	ctgcccaaattcgcgaccggtATGGTGAGCAAGGGCG
	3'R:	tgaaaccagagttaaaggcctcgagGTACAGCTCGTCCATGCC
	Primers for qPCR	
HcSE1-qPCRF	TGGTCGTCGAGTTCATGTGA	
HcSE1-qPCRR	CGGCCATTGTGAAAGCTTCT	
HcSE2-qPCRF	GATCCTTACCCTACACCCGG	
HcSE2-qPCRR	AGATACGTGCTGAGAGTGGG	
HcSE3-qPCRF	GAAGGTTGAGGGAAAAGGCG	
HcSE3-qPCRR	GAGGTTTGCAGAGGTTTCGG	
HcOSC1-qPCRF	CCTCATGCGGATCCAGTTTG	
HcOSC1-qPCRR	ACATCGGACCGCCATAATCT	
HcOSC2-qPCRF	ACCGAACCCCTTCTTACTCG	

HcOSC2-qPCRR	CGTCTTCGGCAACCCATAAG
HcOSC3-qPCRF	CCCAAAACACAGGACCAAGG
HcOSC3-qPCRR	TGGAGAAGGAAGTCGGTAGC
HcOSC4-qPCRF	CATTGTTGGGAAGTTGGGCA
HcOSC4-qPCRR	TTCCATCTTCACCGACCCAT
HcOSC5-qPCRF	ATGGGGTTTATGGACACGGT
HcOSC5-qPCRR	GGCCCTTCACCAAGTAAACG
HcOSC6-qPCRF	CCTGCAGAAACGTTTGGTGA
HcOSC6-qPCRR	CTGCAGCCACCAATCCTTTT
HcACT1-qPCRF	CTGTTTTACCACCACCGCAG
HcACT1-qPCRR	CGTGTTGGCTGCTTTACTGT
HcACT2-qPCRF	AGCTCTTACGGTTCCTTCCC
HcACT2-qPCRR	TGCAGGAATTTTGGTGGCTC
Hc18S1-qPCRF	TCGGGACTTAGTGGGGAAAC
Hc18S1-qPCRR	GCCTTCAATCTTAGTCGCGG
	Primers for Cuol01-1
01RSDN-F	TTTCTTTTTGCTTTTTCTTTTTTTTTCTCTTGAACTCGGCCAGGCGCCTTTATATCATA
01RSDN-R	GACTATAATATTATGCATATAGGA
01PGK-F	CTGAAAACCTTGCTTGAGAAGGTTTTGGGACGCTCGACTACGCACAGATATTATAACATC
01PGK-R	GACCGGATGCAATGCCAATTGTAATAGCTTTCCCATTGTTTTATATTTGTTGTAAAAAGT
01UP-F	GGATATAGGAATCCTCAAAATG
01UP-R	GAGGTCGCTCTTATTGACCACACCTCTACCGGCATGCTGTTGGAATAGAAATCAACTAT
01ADH1-F	ACTAGTTAGTAGATGATAGTTGATTTCTATTCCAACAGCATGCCGGTAGAGGTGTGGTC
01ADH-R	GTTTGAAAGATGGGTCCGTCACCTGCATTAAATCCTAAAGTTATAAAAAAAATAAGTGT
01HMG1-F	ACTTTAAAATTTGTATACACTTATTTTTTTATAACTTTAGGATTTAATGCAGGTGACG

01HNG1-R	CACAAAAACAAAAAGTTTTTTTAATTTTAATCAAAAAATGGCTGCAGACCAATTGGTGA
01HXT7-F	GGTGACTTCAGTTTTCACCAATTGGTCTGCAGCCATTTTTTGATTAAAATTAAAAAAAC
01HXT7-R	CTTCTCGTAGGAACAATTTCG
01TEF2-F-2	GGGGCCGTATACTTACATATAG
01TEF2-R-2	ATGGAGACAACTTCATAGAGGAGGATTCAGATTCCATGTTTAGTTAATTATAGTTCGTT
01synHcCPR1-F	TTAGAATATACGGTCAACGAACTATAATTAACTAAACATGGAATCTGAATCCTCCTCTA
01synHcCPR1-R	AACTAAATCATTAAAGTAACTTAAGGAGTTAAATTTATTACCAAACATCTCTCAAGTAT
01TDH2-F2	GCAAATGAACGGTAGATACTTGAGAGATGTTTGGTAATAAATTTAACTCCTTAAGTTAC
01TDH2-R	CACAGTGATATGCATATGGGAGATGGAGATGATACCTGCGAAAAGCCAATTAGTGTGAT
01ENO2-F	GATAAAGCACTTAGTATCACACTAATTGGCTTTTCGCAGGTATCATCTCCATCTCCCAT
01ENO2-R	ATTCACCCCATTTTTGTTTGGTGAGTTGATTGGTTAATAAAGTGCTTTTAACTAAGAAT
01ERG1-F	CAGAAAAGACTAATAATTCTTAGTTAAAAGCACTTTATTAACCAATCAACTCACCAAAC
01ERG1-R	AATCTATAACTACAAAAAACACATACATAAACTAAAAAATGTCTGCTGTTAACGTTGCA
01TPI-F	TTAATCAATTCAGGTGCAACGTTAACAGCAGACATTTTTTAGTTTATGTATGTGTTTT
01TPI-R	CTGCTCACAAATCTTAAAGTCATACATTGCACGACTATATATCTAGGAACCCATCAGGT
01GPM-F	GGTAATCTTCCACCAACCTGATGGGTTCCTAGATATATAGTCGTGCAATGTATGAC
01GPM-R	CAAGAATCTCTCTCTCCTAATTTCTTTTTCTGAAGCCATTTATTGTAATATGTGTGTTTG
01ERG20-F	TCTTAATAATCCAAACAAACACACATATTACAATAAATGGCTTCAGAAAAAGAAATTAG
01ERG20-R	TCAGGTTGTCTAACTCCTTCCTTTTCGGTTAGAGCGGATCTATTTGCTTCTCTTGTAAAC
01CYC-F	ACTGCGTTCTTGAACAAAGTTTACAAGAGAAGCAAATAGATCCGCTCTAACCGAAAAGG
01CYC-R	GCAAATGCCTATTATGCAGATGTTATAATATCTGTGCGTAGTCGAGCGTCCCAAAACCTT
01PGK-F	CTGAAAACCTTGCTTGAGAAGGTTTTGGGACGCTCGACTACGCACAGATATTATAACATC
01PGK-R	GACCGGATGCAATGCCAATTGTAATAGCTTTCCCATTGTTTTATATTTGTTGTAAAAAGT
01ERG9-F	GGAAGTAATTATCTACTTTTTACAACAAATATAAAACAATGGGAAAGCTATTACAATTG
01ERG9-R	ACTCATTAAAAAACTATATCAATTAATTTGAATTAACTTCACGCTCTGTGTAAAGTGTAT

01FBA-F	TTGGGTTTTATTATATATACACTTTACACAGAGCGTGAAGTTAATTCAAATTAATTGATA
01FBA-R	ACTTCTTGTTGTTGACGCTAACATTCAACGCTAGTATAGTAAGCTACTATGAAAGACTT
01TDH-R	TTCGAGTTCTTTGTAAAGTCTTTCATAGTAGCTTACTATACTAGCGTTGAATGTTAGCG
01TDH-F	TGGATCGTTACCTTGAGCAACCTTCAACTTCCACATTTTTGTTTGTTTATGTGTGTTTA
01synHcOSC6-F	AACTTAGTTTCGAATAAACACACATAAACAAACAAAAATGTGGAAGTTGAAGATTGGTG
01synHcOSC6-R	TAATGTTCTTTAGGTATATATTTAAGAGCGATTTGTTTTAGAACAAGGCAGCTGGATGT
01PGT-R2	TGTTTTGAATGAACAACATCCAGCTGCCTTGTTCTAAAACAAATCGCTCTTAAATATAT
01PGT-F2	CTCTTGCATCTTACGATACCTGAGTATTCCCACAGTTGGTATACTGGAGGCTTCATGAG
01LEU2-F5	GCGCGAAGGACATAACTCATGAAGCCTCCAGTATACCAACTGTGGGAATACTCAGGTAT
01LEU2-R5	AAGGCTAACTCTCAACAGACAACAACACCTGCTTCATTTAAGCAAGGATTTTCTTAACT
01DN-F2	TGCTGTCGCCGAAGAAGTTAAGAAAATCCTTGCTTAA ATGAAGCAGGTGTTGTTGTCTG
01DN-R	AGGAGAACTTCTAGTATATTC
02HcCPR2-F	AATATACGGTCAACGAACTATAATTAACTAAACATGGAATCTGAATCCTCCTCTA
02HcCPR2-R	AATCATTAAAGTAACTTAAGGAGTTAAATTTATTACCAAACATCTCTCAAGTAT
03AtCPR-F	AATATACGGTCAACGAACTATAATTAACTAAACATGTCCTCCTCTTCTTCATCATCCAC
03AtCPR-R	AATCATTAAAGTAACTTAAGGAGTTAAATTTATCACCAGACATCTCTCAAGTATCTACC
04CsCPR-F	ATATACGGTCAACGAACTATAATTAACTAAACATGCAATCGGAATCCAGTTCTATGAAG
04CsCPR-R	AACTAAATCATTAAAGTAACTTAAGGAGTTAAATTTATCACCACACATCACGCAGATAC
05VvCPR-F	AATATACGGTCAACGAACTATAATTAACTAAACATGCAGTCCTCTTCAGTTAAGGTTTC
05VvCPR-R	AAATCATTAAAGTAACTTAAGGAGTTAAATTTATCAGACATCCCTCAAGTATCTACCAG
06PgCPR-F	AGAATATACGGTCAACGAACTATAATTAACTAAACATGGCTGCTATGCCAACTTCTTTG
06PgCPR-R	ATTAAAGTAACTTAAGGAGTTAAATTTATTACCAAACATCTCTCAAGTATCTACCAGTC
	primers with homology arms(Vector construction: for YCplac33-PE
HcCYP87D20-F	cagtcgacctcgaatctagaATCATCCTTTGGTGTGAATTTGATG
HcCYP87D20-R	acatgatgcggccctctagaCAAACAAAATGTGGGCTGCTGCTC

HcCYP81Q59-F	cagtcgacctcgaatctagaATGGAAGTCGCTCAAAGTCAC
HcCYP81Q59-R	acatgatgcggccctctagaATAAATTCAATCATCATCGTAAGCAACGG
HcCYP81Q58-F	cagtcgacctcgaatctagaATGGTTGACTCTAACAGTACCC
HcCYP81Q58-R	acatgatgcggccctctagaTCAAAAGGTTGAGGAAATGGCC
HcCYP87D19-F	cagtcgacctcgaatctagaATGTTGACGGTGGGATTAGCG
HcCYP87D19-R	acatgatgcggccctctagaTTAAGCTTTTTGTGAGAAAGTGATGTG
HcCYP90B-F	cagtcgacctcgaatctagaATGTTTCATCCAGAACTAGTACTC
HcCYP90B-R	acatgatgcggccctctagaTTATTGGTCGTTATCATGCTCGG
Hcoxidoreductases1-F	cagtcgacctcgaatctagaATGGCTGCTGCAGTCTCAG
Hcoxidoreductases1-R	acatgatgcggccctctagaTCATCTAAAGGTACGAAGTGAGTG
Hcoxidoreductases2-F	cagtcgacctcgaatctagaATGAGTTCCAATGGTCAATCTCC
Hcoxidoreductases2-R	acatgatgcggccctctagaTCATTGCCCTAAAGTTTTGGC
Hcoxidoreductases3-F	cagtcgacctcgaatctagaATGAACGAATCGTCGTCTACC
Hcoxidoreductases3-R	acatgatgcggccctctagaTCACACCGACGATAACTTACTAAC
CsCYP87D20-F	cagtcgacctcgaatctagaATGGAATTGATCATGAACGTCTGTG
CsCYP87D20-R	acatgatgcggccctctagaTCATTGGGAGACCCTGACAATACG
MeCYP87D20-F	cagtcgacctcgaatctagaATGTGGACCATCTTGTTGGGTTTA
MeCYP87D20-R	acatgatgcggccctctagaTCATTCTTTTGGGGTGAAGTTAA
ClCYP87D20-F	cagtcgacctcgaatctagaATGTGGACTATCTTTCTCGGTTTCGTGACGCTG
ClCYP87D20-F	acatgatgcggccctctagaGATGGGCTACATGTGAACTTCACTCCCAAGGAATGA
	Primers for DNHc87 , DNCl87, DNCs87and DNCm87
X4-Up-F	TCTGGTGAGGATTTACGGTATGATCATG
X4-UP-R	agactgtcaaggagggtattctgggcctccatgtcgctgAACAGGCATGGGAAGATTCG
KANMX-F	AATTCAAAAAAAAAAAGCGAATCTTCCCATGCCTGTTcagcgacatggaggcccagaat
KANMX-R	CATTATGCAACGCTTCGGAAAATACGATGTTGAAAAttcgacactggatggcggcgtta

PRM9-F	ctgtcgattcgatactaacgccgccatccagtgtcgaaTTTTCAACATCGTATTTTCCG
PRM9-Hc87D20-R	TGGGCTACATATTAAGTTCACTCCTAAGGATGATTGAACAGAAGACGGGAGACACTAGC
PRM9-Cm87D20-R	AGATGGTCTGTACGTTAACTTCACCCCAAAAGAATGAACAGAAGACGGGAGACACTAGC
PRM9-Cs87D20-R	AGATGGGTTATATGTGAACTTCACTCCCAAGGAATGAACAGAAGACGGGAGACACTAGC
PRM9-Cl87D20-R	TGATGGTTTGCATGTTAATTTCACTCCTAAAGAATAAACAGAAGACGGGAGACACTAGC
Hc87D20-F	GTGCTAGTGTCTCCCGTCTTCTGTTCAATCATCCTTAGGAGTGAACTTAATATGTAGCC
Hc87D20-R	TAGAAAGAAAGCATAGCAATCTAATCTAAGTTTTAATTACAAAATGTGGGCGGCGGCGC
Cm87D20-F	GTAAAGTTGTGTGCTAGTGTCTCCCGTCTTCTGTTCATTCTTTTGGGGTGAAGTTAACG
Cm87D20-R	AGCATAGCAATCTAATCTAAGTTTTAATTACAAAATGTGGACCATCTTGTTGGGTTTAG
Cs87D20-F	GGTAAAGTTGTGTGCTAGTGTCTCCCGTCTTCTGTTCATTCCTTGGGAGTGAAGTTCAC
Cs87D20-R	AAGAAAGCATAGCAATCTAATCTAAGTTTTAATTACAAAATGTGGACGATCTTGCTCGG
Cl87D20-F	GTGTGCTAGTGTCTCCCGTCTTCTGTTTATTCTTTAGGAGTGAAATTAACATGCAAACC
Cl87D20-R	TAGCAATCTAATCTAAGTTTTAATTACAAAATGTGGACCATTTTTTTGGGTTTTGTTAC
Hc87D20-TEF1-F	ACCAAACAAAGCGCCGCCGCCCACATTTTGTAATTAAAACTTAGATTAGATTGCTATGC
Cm87D20-TEF1-F	GCTAAACCCAACAAGATGGTCCACATTTTGTAATTAAAACTTAGATTAGATTGCTATGC
Cs87D20-TEF1-F	GCCAAACCGAGCAAGATCGTCCACATTTTGTAATTAAAACTTAGATTAGATTGCTATGC
Cl87D20-TEF1-F	ACAAAACCCAAAAAAATGGTCCACATTTTGTAATTAAAACTTAGATTAGATTGCTATGC
TEF1-R	AACACTGGGGCAATAGGCTGTCGCCATTCAAGAGCAGAGTGATCCCCCACACACCATAG
X4-DOWM-F	GTAGAAACATTTTGAAGCTATGGTGTGTGGGGGATCACTCTGCTCTTGAATGGCGACAG
X4-DOWM-R	CCCAAAGCTAAGAGTCCCAT
rDNA-UP-F	GAACTGGGTTACCCGGGGCACCTGTC
rDNA-UP-R	GAGTAAAAAAGGAGTAGAAACATTTTGAAGCTATTTCCTCTAATCAGGTTCCACCAAAC
UASTDH3-F	CGGGGTATCTGTTTGGTGGAACCTGATTAGAGGAAATAGCTTCAAAATGTTTCTACTCC
UASTDH3-R	CAAAGTAGCTAAACCCAACAAGATGGTCCACATTTTGTTTGTTTATGTGTGTTTATTCG
CmCYP87D20-F	AGAACTTAGTTTCGAATAAACACACATAAACAAACAAAATGTGGACCATCTTGTTGGG

CmCYP87D20-R	AAAAAAAAAATCTTTGACTATTCAATCATTGCGCTCATTCTTTTGGGGTGAAGTTAACG
CPS1t-F	GATGGTCTGTACGTTAACTTCACCCCAAAAGAATGAGCGCAATGATTGAATAGTCAAAG
CPS1t-R	GATTCGATACTAACGCCGCCATCCAGTGTCGAATTTGACACTTGATTTGACACTTCTTT
G418-F	CATAAATAAAAAAAAAAAGAAGTGTCAAATCAAGTGTCAAATTCGACACTGGATGGCGG
G418-R	CTACTATAAAACAACCTTTAGACTTACGTTTGCTACTCTCATCAGCGACATGGAGGCCC
rDNA-DOWN-F	ACTGTCAAGGAGGGTATTCTGGGCCTCCATGTCGCTGATGAGAGTAGCAAACGTAAGTC
rDNA-DOWN-R	CTCACTATTTTTTACTGCGGAAGCGG

Supplemental Table 12. Sequence information of candidate genes and accession numbers in this study.

Gene name	Amino acid sequence	NCBI accession numbers
HcSE1	MIVTGRDRRRDCDLFHGGSEAKEW*SISFGGMREERFHEKWRMHI*RWRRRRYYRRSRRCWFGSCSHARQGWSSSSCD*KRLDRA**NCW*IITTWRLPQINRVRT*RLC*GN*CTKGVWLCPFQGWEEYSTLLPFGKISL*CLWEKLSQWPLHTENEREGCFTSQCEIGARHSYFTA*RKGNNQRCAV*V*KR*RKNSKCTSDHCL*WLLLKLAPVSLHPYG*CALLFCGIGSGELQSSFCKSWACCAWRSISYAVLSH*QH*GPLFGRCSWSESSFDIKR*NGEIFEDCNSSSGSSSNL*FLYGCY*QG*HKDNAKQKHACSSSPDTRCLING*RIQYAPPSYRWRNDCGIV*YCGTSKPPQASERLE*FINSLQVS*IILHSPQACGFHHKHIGRIIIQGFLCITRSS*EGNATSLF*LFEPRRNVLKWTRLFTFRIKSSTVKFGSPFLCRCDIWSWSLVTSISFTKTHLDWS*IDP*CSRNYISDHKG*RS*TDVLPCNCSCLL*KSTRFRTHFL	OR474384
HcSE2	MPNQFNLGWILASVLGAASLYLFFGKKNFRVSREPRRDSLKNIATTNGECKSTNSDGDIIIVGAGVAGSALAFTLGKDGRRVHVIERDLTEPDRIVGELLQPGGYLKLTELGLEDCVDDIDAQRVYGYALFKDGKDTRLSYPLEKFHSDVAGRSFHNGRFVQRMREKAATLPNVQLEQGTVTSLLEENGVIKGVQYKSKTGQEMTAYAPLTIVCDGCFSNLRRSLCNPKVDVPSCFVGLVLEDCELPYANHGHVILADPSPILFYPISSTEIRCLVDVPGQKVPSISNGEMANYLKSVVAPQIPCQLYDAFIAAIDKGNIRTMPNRSMPADPYPTPGALLMGDAFNMRHPLTGGGMTVALSDIVVLRDLLKPLRDLHDAPTLSTYLEAFYTLRKPVASTINTLAGALYKVFCASPDQARREMREACFDYLSLGGVFSNGPVSLLSGLNPRPLSLVLHFFAVAIYGVGRLMIPFPSPKRVWIGAKLISGASGIIFPIIKAEGVRQMFFPATLPAYYRAPPVRTEL*	OR474385
HcCPR1	MESESSSMKLSPLEFMTSIIKGKSDPSNSSFNSSGEVSSIVFENRELIAILTSSIAVLIGCFVLLVWRRSNSQKAKTVELPKPLIVKEIEPEVEDGKKKVTIFFGTQTGTAEGFAKALADEAKARYEKATFKVVDLDDYAGEDDEYEKLNKESFALFFLATYGDGEPTDNAARFYKWFTEGKERGEWLQNLHYGVFGLGNRQYEHFNKIAKVVDELFEEQGGKRLVKVGLGDDDQCIEDDFTAWRESLWPELDQLLRDEDDATTVATPYTATVLEYRVVFNDPADAAAEDESWNVANGHAVHDAQHPYRSNVAVRKELHSPASDRSCTHLEFDISGSALTYETGDHVGVYCENLTETVEEALNLFGLSPETYFSIHTDNEDGTPIGGGSLPPPFASCTLRVALTRYADLLNSPKKSALLALAAHASNPIEADRLRYLASPAGKDEYSQSVVGVQKSLLEVMAEFPSAKPPLGVFFAAVAPRLQPRYYSISSSSRMAPSRIHVTCALVYDKMPTGRTHKGVCSTWMKNSVSVEKSHECSWAPIFVRQSNFKLPADSKVPIIMIGPGTGLAPFRGFLQERLALKESGVEMGPSMLFFGCRNRRTDFIYEDELNNFVETGALSELVIAFSREGPTKEYVQHKMAEKASEIWNLISNGAYLYVCGDAKGMARDVHRTLHTIVQEQGSLDSSKAESMVKNLQMNGRYLRDVW*	OR474394
HcAT1	MALPKVKIISEERIKPSSPTPESQKYLNFSLLDQLALPVYVPLLLFYVGGGDCEDANERSRSLKVSLSDELTRFYPLAGRVKEDNESIFCNDEGAIYVEAKANCLLSDFLNQLEIDSLNDFLPFDSAKGCILLVQITSFECGGMAIGLLMSHKISDASSISAFIKSWTATSRGCKLSESELPKFIGASVLPPPQDFPISTPTSDSGIHAKGVTKRLVFEASKIIELKAKATSATVKQPTRVEAVTGLIWKCAIAASKSTSGISKPSVVGQAVNLRKRLVPTLPDTSIGNLLGFITPETKTEAGEIELQGLVGLLREGITEFNENGFKKYQDTEAYLTYFKTLMNPDGPYGGDKNFYLCSSWSRFQFYEADFGWGCPVWFIGGISMFSNFFLLMDTKDGRGIEALVTLSEEDMALFQRDEDLLAYGSINPKSIRL*	OR474392

HcOSC1	MWQLKIGTDTVPAAADPSNGGGWLSTLNDHVGRQIWHFNPEAGTPEELQQIQNARQRFFDNRFEKTHSDDLLMRIQFAKESSSFVNLPQLKVK<br>DKEDVNDESVIRTLRRAINFYSTIQAHDGHWPGDYGGPMFLIPGLVITLSITGALNAVLSTEHQREICRYIYNHQNKDGGWGLHIEGPSTMFGSV<br>LNYVTLRLLGEEIEDGQGAMDKAQKWILDHGSATAITSWGKMWLSVLGVYEWAGNNPLPPEVWLCSYLLPCHPGRMWCHCRMVYLPMCYL<br>YGKRFVGPITPIIRSLRRELYHIPYHEVDWNEARNQCAKEDLYYPHPLVQDVIWASLHYVYEPIFMHWPAKRLREKALQSVMQHIHYEDENTRY<br>ICIGPVNKVLNMLCCWVEDPHSEAFKLHIPRIFDYLWIAEDGMKMQGYNGSQLWDTAFAVQAIISTNLAEEYGETVRKAHKYIKDSQVLEDCP<br>GDLQFWYRHISKGAWPFSTADHGWPISDCTAEGLKAILLLSELPSEIVGKSIDEERIYNAVNVILSLQNAHGGFATYELTRSYNWLELINPAETFG<br>DIVIDYPYVECTSAAIQALAMFKKLHPNHRKDEIDNCIAKAAKFIESIQATDGSWYGSWGVCFTYGGWFGIRGLVAAGRTYGNCSSIRKACDFL<br>LSKELATGGWGESYLSCQNKVV*	OR474386
HcOSC2	MWRLKIADDGGGNDPYLYSTNNFVGRQTWEFDPQAGTPQEREQVEQARLHFYQNRYQVKPSSDLLWRMQFVREKKFKQEIGAVKIEEDEEIS<br>EEKASDALRRAVKFYSAMQASDGHWPAENAGPLFFLPPLVMCVYITGHLDQVFPSEYKKEILRYIYYHQNEDGGWGLHIEGHSTMFCTALSYI<br>CIRILGQGPHGGLNNACSRARTWILDHGGVTYIPSWGKTWLSIFGVYDWSGSNPMPPEFWMLPSFLPMHPAKMWCYCRMVYMPMSYLYGKR<br>FVCEITPLIQQLRQELHTQPFDEINWKKTRHLCAQEDLYYPHPLIQDLLWDSLYICTEPLLTRWPFNKLVRDKALQLTMKHIHYEDENSRYITIGC<br>VEKVLCMLACWVEDPNGDYFKKHLARIPDYLWVAEDGMKMQSFGSQQWDTGFAIQALLAADMADEIGPTLAKGHDFIKKSQVKDNPSGDFK<br>SMYRHISKGSWTFSDQDHGWQVSDCTAEGFKCCLLFSMMPPELVGEKMEPQRLYDSVNILLSLQSKNGGLAAWEPATGHDWLEMLNPTEFFA<br>DIVIEHEYVECTGSAIQSLILFKKLHPGHRKKEIDNLIKKAVGYLEHIQNEDGSWYGNWGVCFTYGCWFAIGGLVASGKTFNNCASIRKGVHFLL<br>NIQMPDGGWGESYLCCPNKEYVPLEGNRSNLVHTAWAMMALIHSGQAERDPTPLHRSAKLIINSQMEDGDFPQQEITGVFMKNCMLHYAAYR<br>NIYPLWALAEYRRRVKLPSTSF*	OR474387
HcOSC3	MWRIKIADGGNNPYIFTTNNFVGRQIWEFDPEAGTQEERQQVEEARLNFYQNRHKVKPSSDLLWRMQLLKEKNFKQEIPPIKVEEGEEMSREKVSG<br>ALRRAVHFYSALQASDGHWPGENAGGLYFLPSLVICLYSIGHMEKVFGSEQEKKEILRYIYCHQNKDGGWGLYIVGQSSMLGTVFNYICMRLLGE<br>DTDGGHNNACTKARNWILDHGGATHIPTWGKTVLSLLGVYEWAGHNPVPPELFLLPSFFPFHPAKLWCYCRSLYLPTSYLYSKRFVGKITPLIQELR<br>QEIYTQPYHEINWKKARHLCSKEDIYYPHPFFQDLLWDGLYFLTEPLLTHWPFNKLIREKAIELTMDYIHYDEENTRYITMCCLSKALGIVACWVED<br>PNGEAFKNHLARIPDFIWVGEDGMKMQTSGSQEWDASLAVQALLAADMADEIGHTLASGHDFIKKSQVRNNPSHDFRRMCRDISKGAWTFSDQD<br>NGLPVSDCTAEALKCCLLFSWMPPEIVGEKMEAERLYDAVNFLLYLQSKNGGLTAWEKGGNYLWLEKLNPTDIFGVMMIEHETLECTSSGIQALIL<br>FKKLHPKHRTKEVETFITNAVGYILDSQLSDGSWYGNWGICFIYGTWFAIKALVTSGKSYDNCIAIRKATDFLLQIQRPDGGWGESYLSSPKMKYIPL<br>DGDDESNLVQTAWAMMSLIHAGQAERDPTAIHRAARLIINSQLEDGDFPQQGITGVSLGNCFLHYSNYRNIFPLWALAEYRRSLQFSST*	OR474388
HcOSC4	MWRLKLGEGANDPYLFTTNNFVGRQTWEFETQADDATLLSRAQVEDARNNFYQNRFNIKPSSDLLWKFQFLEEKNFKQAIPKVKVEEEKGQGI<br>TIKAETATIALRRATAFFAALQSSHGHWPAENSGPLCYFPPLVFALYITGHLNTIFSEEHRKEIIRYTYCHQNEDGGWGLDIAAESCMLCTVLNYV<br>QLRILGVEEGADNIDACGRARKWILDHGGALYIPSWGKIWLAILGVYEWGGTIPMPPEVWMIGHNVPLNPGSLLCYCRLTYLPMSYLYGKRFV<br>GPLTPLILQLRQEIYRQLYHEIKWSPARHYCAKEDACFKRPLLQKLAWDVVYYVGEPLLGSWAFKTLRNRALQVTNQIMDYEDHASRYITIGC<br>VEKPLLMVAKWVEDPNGEAYKKHLARVKDYIWVGEDGIKMQSFGSQSWDVAFAMQAMLATNLHREFSDTLKKGHDFIKKSQIRENPPGDFK	OR474389

	SMYRHISKGAWTFSDRDHGWQVSDSTAENLECCLKLSTLPSDLVGDPMEPECFFDAVNILLSLQVKNGGLGAWEPTAPAIRPWIEQLNPVEFLE<br>YSVMEIETVECVSSSIQALAVFMKVFPRHRRKEIESFIRRGVKYIEENQKEDGSWYGNWGICYLYGTFFGLKGLEAAGKTYENCMAVRRGVQFL<br>LSKENEGGGWGESHESCTKEKQYKPLLQPNLVQTSWALMALIHAQQAERDPSPLHRAARLLINSQLEDGDYPQQELTAVFMKNCMLHYALYR<br>NVFPLWALAEYRNLVALSSLGVI*	
HcOS<br>C5	MWRLKIADGGNDPYIYSMNNFVGRQIWEFDPEAGTAEERAEVEKVMSNFTKNRYKGFPSADLLWRMQLLREKNFKQSIPKVKVKDGEEISYE<br>MASDAMKRGAYFLSALQASDGHWPSETSGPLFYICPLIICMHIMGFMDTVFSWEHKKEILRYIYNHQNEDGGWGLHVGGHSNMFCTTFNYISM<br>RLLGEGPNGGLNDAVSRARNWIHNHGGVTSIPSWGKTWLSILNVFDWPASNPMPPEYWMLPTWIPIHPSNMMCYTRITYMPMSYLYGKRFQA<br>PLTPLILQLREELHTQPYHQINWKKVRHMCATEDLYFPHPFVQDLLWDTLYLMSEPLMTRWPFNKLVRQKALQQTMKHIHYEDENSRYITIGC<br>VEKPLCMLACWIDDPNSDYVKKHLARIPDYLWMAEDGMKMQSFGSQSWDAALAMQALLACNIPHDIQITPTLNNGHNFIKNSQVRNNPPGDY<br>KSMFRYMSKGSWTFSDCDHGWQVSDCTAENLKCCLLLSLLPSESVGEKMKPQRFYDALNVILNMQSKNGGLPAWEPASSNYWMEWLNPVEF<br>LEDLIIEHQHVECTSSALQAILLFRKQYPSHRRKEINNFINKAVQFLQDIQLPNGSWYGNWGICYTYGTWFALKALSMAGKSYQNCEAMRKGA<br>HFLLKIQNAEGGFGESYLSCPYKRYIPLDGKRSNLVQTAWGLMGLISAGQADVDPAPIHGAAKLLINSQTEEGDFPQEEITGEFFKNCTLHFAAF<br>REVFPVMALGEYRNKVPLPSSKQP*	OR47439<br>0
HcOS<br>C6	MWKLKIGGESVGKNDERLLKTVNNHLGRQVWEFSNESDSDSDSDSHHFQIDEARNTFYHNRFHQKQSSDLFIRIQYGKEISSGGKIGGIKLKESE<br>EVSGEAVKTTLERALNFYSAIQTSDGNWASDLGGPMFLLPGLIISLYVTGVLNSVLSKQHRQEICRYIYNHQNEDGGWGLHIEGPSTMFCSVLN<br>YVALRLLRKDPYEDSMPKARLWILDHGGATAITSWGKLWLSVLGVYEWSGNNPLPPEFWILPYFLPFHPGRMWCHCRMVYLPMSYLYGKRF<br>VGPITPTVLSLRKELYTVPYHEIDWNKSRNTCAKEDLYYPHPKMQDILWGSIHHVYEPFFTRWPGKRLREKALDAAMQHIHYEDENTRYICLGP<br>VNKVLNMLCCWVEDPHSEAFKLHLERVHDYLWVAEDGMKMQGYNGSQLWDTAFSVQAIISTKLTDNFGPTLRKAHDFIKNSQIRQDCPGDPN<br>IWYRHIHKGAWPFSTADHGWLISDCTAEGLKAALLLSKLSSETVGEPLERNRLYDAVNVLLSLQNENGGIASYELTRSYPWLELINPAETFGDIV<br>IDYPYVECTSASIEALALFKKLHPGHRTKEIENAVAKAAKFLEDMQREDGSWYGCWGVCFTYAGWFGIKGLVAAGRKYNNCPTIRKACNFLLS<br>KELPGGGWGESYLSCQNKVYTNLEGNRPHLVNTAWALMALIEAGQYEKDPTPLHRAARLLINSQLENGDFPQEEIMGVFNKNCMITYAAYRNI<br>FPIWALGEYCHHVLNEQHPAALF*	OR47439<br>1
HcCY<br>P87D<br>20	MWAAALCLVVLLVVYYTHWVNKWKNSKFNGVLPPGTMGLPLIGETIQLSRPSDSLDVHPFIQRKIQRYGPIFKTCLAGRPVVVSVDAEFNHYI<br>MLQEGRAVEMWYLDTLSKFFGLDTEWLKALGLIHKYIRSITLNHFGAESLRERFLPRIEESSREALRFWSTQSSVEVKDAAAAMVFRTSVKKMF<br>GEDASKLSAELTVKFTKLLGGFLTLPLNIPGTTYNKCLKDMREIQNKLKEILAERQASPNKLDGEDFLGQAIKDKETEKFISEEFIIQLLFSISFASF<br>ESISTTLTLILKFLSENPSVIKELEAEHEAIRNARENPDSPVTWEEYKSMTFTLQVINETLRLGSVTPALLRKTTKEIQIKGYTIPEGWTVMLVTASR<br>HRDPEIYKEPHLFNPWRWKEMDSITIQRNFMPFGGGLRHCAGAEYSKVYLCTFLHILFTKYRWTKIKGGKIARAHILKFEDGLHIKFTPKDD	OR47439<br>3

Supplemental Table 13. NCBI identification numbers of BAHD-AT sequences used in the phylogenetic analysis.

AT Name	NCBI Accession ID	Species
SmaAT1	EFJ35925	*Selaginella moellendorffii*
SmaAT2	EFJ34857	*Selaginella moellendorffii*
CcAT1*	AXB26761	*Crocosmia x crocosmiiflora*
CcAT2*	AXB26762	*Crocosmia x crocosmiiflora*
At3AT1	NP_171890	*Arabidopsis thaliana*
Gt5AT*	BAA74428	*Gentiana triflora*
Dv3MAT*	AAO12206	*Dahlia variabilis*
Pc3MaT*	AAO38058	*Pericallis cruenta*
NtMAT1	BAD93691	*Nicotiana tabacum*
ZmGlossy2*	CAA61258	*Zea mays*
AtCER2*	CAA61258	*Arabidopsis thaliana*
Ss5MaT2	AAR26385	*Salvia splendens*
FvVAAT*	CAC09062	*Fragaria vesca*
PsSalAT*	AAK73661	*Papaver somniferum*
CaPun1*	AAV66311	*Capsicum annum*
HvACT*	AAO73071	*Hordeum vulgare*
AtHCT*	NP_199704	*Arabidopsis thaliana*
AtCHAT*	AAN09797	*Arabidopsis thaliana*
TcTAT*	AAF34254	*Taxus cuspidata*
TpHCT1A	ACI16630	*Trifolium pratense*
CsaAT1*	NP_001295855	*Cucumis sativus*
CsaAT2*	NP_001295806	*Cucumis sativus*
CmAT1*	XP_008459937	*Cucumis melo*
